# Planar-bilayer activities of linear oligoester bolaamphiphiles

**DOI:** 10.3762/bjoc.7.184

**Published:** 2011-11-22

**Authors:** Jonathan K W Chui, Thomas M Fyles, Horace Luong

**Affiliations:** 1 Department of Chemistry, University of Victoria, Victoria BC, Canada

**Keywords:** activity grid, single-molecule studies, synthetic ion channels, voltage clamp experiment

## Abstract

Voltage-clamp experiments of eight oligoester bolaamphiphiles in two subclasses are described. Syntheses of three new terephthalate-based compounds were achieved in three linear steps. Together with five previously described, related compounds, the ion transport activity was assessed by means of the voltage-clamp technique. All of the compounds show multiple types of conductance behavior in planar bilayers, a subset of which was exponentially voltage-dependent. The varied and irregular activities were summarized with the aid of a recently developed “activity-grid” method.

## Introduction

Ion channels modulate conductance in cell membranes, and form the basic elements from which action potentials and other complex electrophysiological behaviors are constructed [1]. With recent technological breakthroughs, such as stochastic sensing [2] and the construction of ionic networks [3–4], the control and understanding of ion-channel attributes at a single-molecule level takes on a new urgency. By being able to tailor molecular features through synthesis, and potentially being accessible in far larger quantity than membrane proteins, de novo channels promise to be useful entities for structure–activity studies as well as practical “ionic components” of more complex systems. Since the pioneering report by Tabushi [5] some three decades ago, hundreds of disparate synthetic compounds have been shown to behave as ion channels, by ensemble and/or single-molecule techniques [6–7]. Ensemble techniques refer to the many variations of vesicle-based assays [8]; the results obtained are comparable under identical conditions, but often incommensurate with different variants of the method.

The voltage-clamp technique, on the other hand, reports absolute flux under defined conditions [9]. In this method, a planar bilayer is formed in an aperture between two electrically connected aqueous compartments; this forms an insulator between the electrodes, and no current flows despite an applied potential across the electrodes. Upon addition of the compound forming the active channel, a current on the order of pA can be detected corresponding to the activity of single or small groups of molecules, and this current can be monitored with millisecond time resolution or faster. Thus, this method can report not only the flux but also the structural dimensions and dynamic properties of the channel with high sensitivity; however, the voltage-clamp experiment suffers from a need for specialized equipment, as well as the daunting task of making sense of all but the most regular of activities.

We have recently developed an empirical classification scheme for summarizing the range of voltage-clamp activities [10]. This “activity grid” represents the conductance of an event as a vertical position on a logarithmic scale in half log-unit steps from 3 to 3000 pS, the duration of these events as horizontal position on a logarithmic scale from 10 ms to 100 sec, and the pattern of activity as a different color shading (green for discrete “square-top” behavior, yellow for “flickering”, blue for discrete transitions with irregular opening, red for short duration spikes, purple for “erratic” activities from which no pattern was discerned, and grey for regions of the grid where the experimental setup precludes detection of events). A systematic evaluation of published synthetic-ion-channel conductance records revealed an underlying commonality over the wide range of structural types. However, published records are typically a brief excerpt of full activities and we wondered how the methodology would fare in the real world. How would the method apply to full sets of unabridged records? And are there phenomena that are beyond the scope of this representation?

**Scheme 1:**
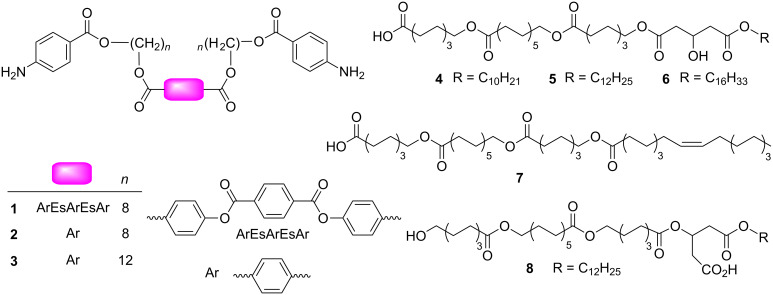
Compounds studied with the voltage-clamp experiment.

To provide a suitably focused yet sufficiently diverse suite of molecules in order to answer these questions, two classes of bolaamphiphiles were selected (Scheme 1). Both the phthalate and solid-phase derived alkyl compounds derive from the de-macrocyclization of an earlier active channel [11]. Structurally they similarly consist of a linear topology linked by internal esters (Es); the classes differ by the presence of multiple aromatic rings in the phthalates, and the lack of centrosymmetry in the alkyl compounds. These were expected to be active in voltage-clamp experiments, as close analogues to the phthalate compounds were found to be active in vesicles and planar bilayers [12], and the activities of the solid-phase compounds in vesicle experiments were previously established [13].

## Results and Discussion

### Synthesis of new compounds

**Scheme 2:**
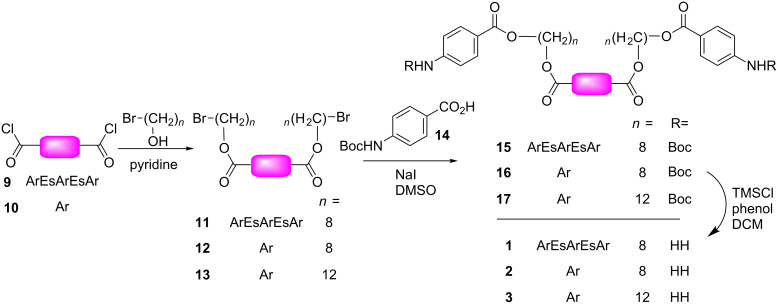
Synthesis of phthalate compounds.

The solid-phase synthesis of the alkyl oligoesters was previously reported [14]. The phthalate compounds were prepared by a modular methodology (Scheme 2), beginning by reacting terephthaloyl chloride or compound **9** (prepared by a method adapted from [15]) with α,ω-bromoalcohols prepared by mono-brominating the corresponding diols as described [16]. This gave the bis-bromides, which were subsequently displaced by the protected carboxylate anion of BOC-protected aniline **14** to give precursors **15**, **16** and **17**.

Deprotection of the carbamate protecting groups under various acidic conditions also resulted in cleavage of internal esters in **15**. Selective cleavage was ultimately achieved by use of a TMSCl–phenol reagent [17] in dichloromethane to give the desired targets in three linear steps with modest overall yields. All three products were white solids that are freely soluble in chloroform; compounds **2** and **3** are soluble in methanol whereas **1** is only sparingly soluble. All three compounds precipitate from water at micromolar concentration or above.

### Voltage-clamp results

Voltage-clamp studies were performed as previously described [12]. The method of introducing the compounds is limited by their solubility: Water-soluble compounds (**4**–**8**) were generally introduced as a solution to the bathing electrolyte, sparingly soluble **2** and **3** by injection close to the proximity of the bilayer, and the insoluble compound **1** was added by physical transfer from a brush. Physical transfer often results in membrane rupture, and the integrity of the membrane cannot be guaranteed; nonetheless, an analysis of different methods of compound introduction showed no substantial differences in compound activity [18].

**Figure 1:**
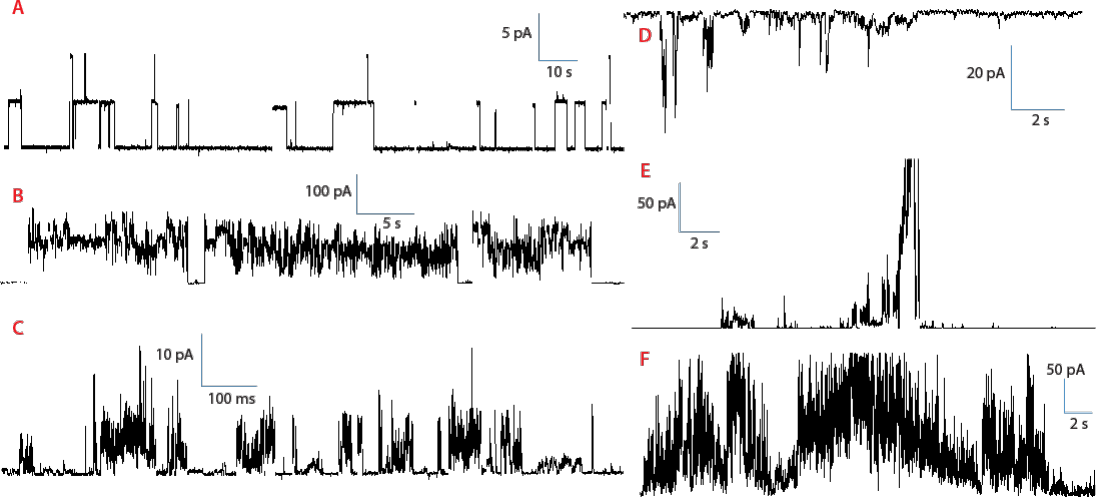
Channel activities of compound **1**. **A**: Regular “square-top” activity. Conditions: 1 M KCl buffered to pH 4.5, +200 mV; **B**: Large semiregular activity. Conditions: 1 M CsCl unbuffered, +130 mV; **C**: Short-lived semiregular openings. Conditions: 1 M KCl unbuffered, +100 mV; **D**: Short-lived, low-conductance bursts. Conditions: 1 M CsCl unbuffered, −50 mV; **E**: High-conductance bursts. Conditions: 1 M CsCl unbuffered, +120 mV; **F**: Continuous erratic activity. Conditions: 1 M CsCl unbuffered, +160 mV.

**Figure 2:**
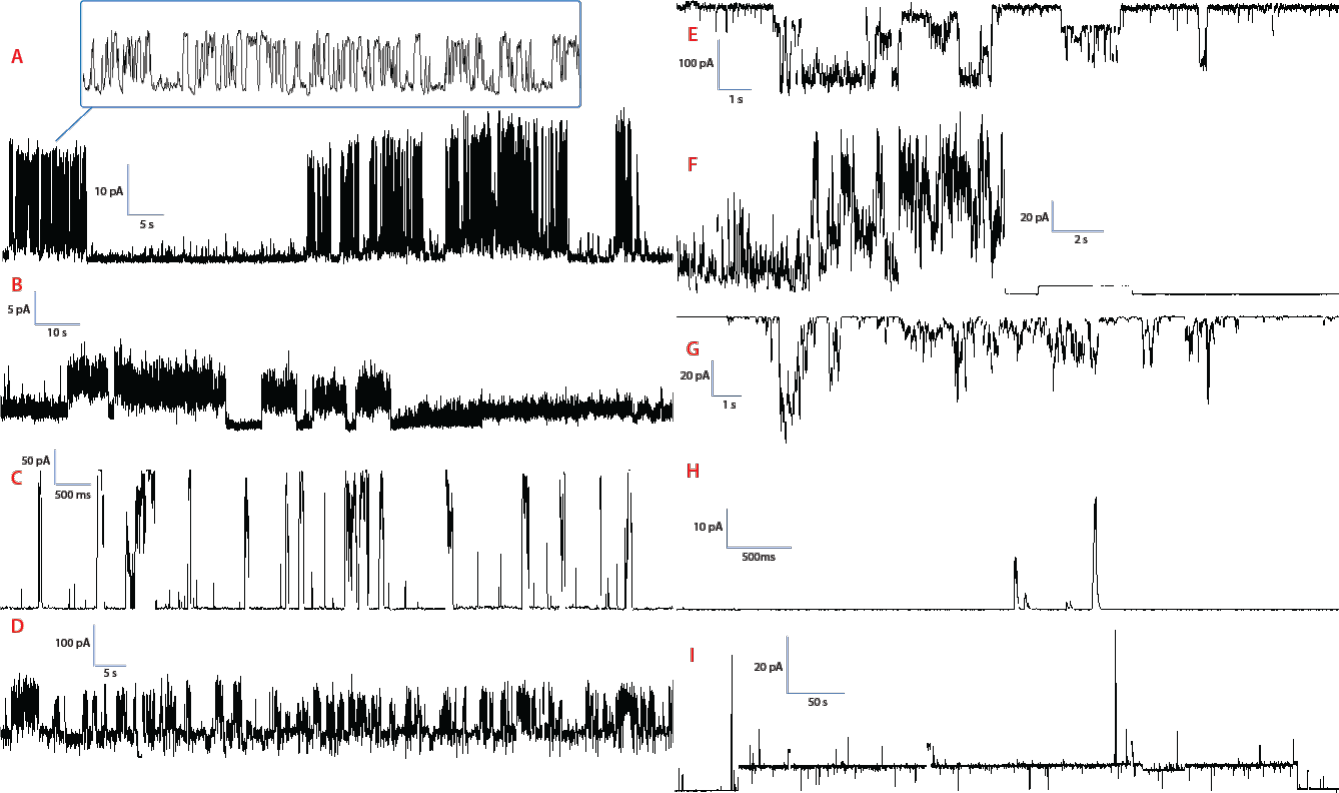
Channel activities of compound **2**. **A**: Rapid flickering. Conditions: 1 M CsCl, +100 mV; **B**: Semiregular openings. Conditions: 1 M CsCl, +100 mV; **C**: Regular high-conductance bursts. Conditions: 1 M CsCl, +120 mV; **D**: Long-lived opening punctuated by regular bursts. Conditions: 1 M CsCl, +100 mV; **E**: High-conductance, quasiregular activity. Conditions: 1 M CsCl, −120 mV; **F**: Abrupt closure following sustained bursts. Conditions: 1 M CsCl, pH 7, +120 mV; **G**: Short-lived, high-conductance bursts. Conditions: 1 M CsCl, pH 7, −120 mV; **H**: Brief spikes. Conditions: 1 M CsCl, pH 7, +180 mV; **I**: Long-lived regular opening, with conductance sublevels. Conditions: 1 M CsCl, pH 7, +170 mV.

Excerpts of experimental data are shown in Figure 1 and Figure 2 for compounds **1** and **2**, respectively. These two figures establish that a great variety of activities coexist for these two compounds, and that the regular “square top” behavior is a minority-type among many different types of irregular behavior. Complete experimental summaries are given in the Supporting Information File 1, from which it is clear that we could have made similar representations as Figure 1 and Figure 2 with any other pair of compounds. Erratic behaviors may be aesthetically unappealing but should come as no surprise: The hydrocarbon chains that are present in every compound have significant conformational freedom; a variety of aggregation states are accessible, and an anisotropic membrane presents different local environments and other time-dependent degrees of freedom, which can be aptly collected under the umbrella term “dynamic supramolecular polymorphism” [8].

A theoretical framework and tools for extracting mechanistic information from the modeling of ideal “square-top” opening/closing events is well-developed, and statistical approaches may be appropriate when large numbers of events (> 10^4^) are at hand [19]. The majority of the observations made in this study conform to neither of these criteria, and we were unable to visually discern any obvious patterns; for this reason we turned to the summary activity grids as a (potentially) model-independent method for recording diverse behaviors.

**Figure 3:**
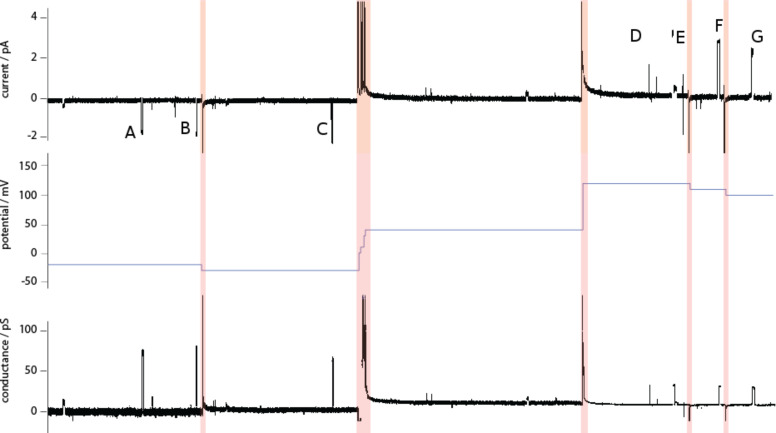
Conversion of a current–time profile of compound **5** (top) into conductance–time profile (bottom) by use of the applied potential (middle). (Red bands indicate changes of the potential; the spikes appearing in these periods are the capacitive currents from the instrument. The conductance is undefined when the applied potential is 0 mV, and thus this results in a break in the centre of the conductance–time profile.)

Preparing the “activity grid” from empirical current–time traces was facilitated by a suite of custom software, the development of which was described elsewhere [18]. There are two steps involved. The first involves the systematic conversion of current–time profiles, such as those given in Figure 1 and Figure 2, into conductance–time profiles from the known applied potentials. A simple example is given in Figure 3 which, when viewed from the experimental current–time perspective (top panel), shows (i) negative currents initially and positive currents over time, that (ii) events **A** and **B** are visually of lower magnitude than events **D**–**G**, and (iii) events **D**–**G** are of decreasing magnitude. Correcting for changes in the applied potential (middle panel) allows direct comparisons to be made, and it is clear from the bottom panel of Figure 3 that events **A** and **B** have a conductance at least twice as large as events **D**–**G**, and that the unit conductance of events **D**–**G** are in fact identical. Simple cases such as this could be resolved by a skilled observer, but more complex cases require full conversion to a conductance–time representation.

**Figure 4:**
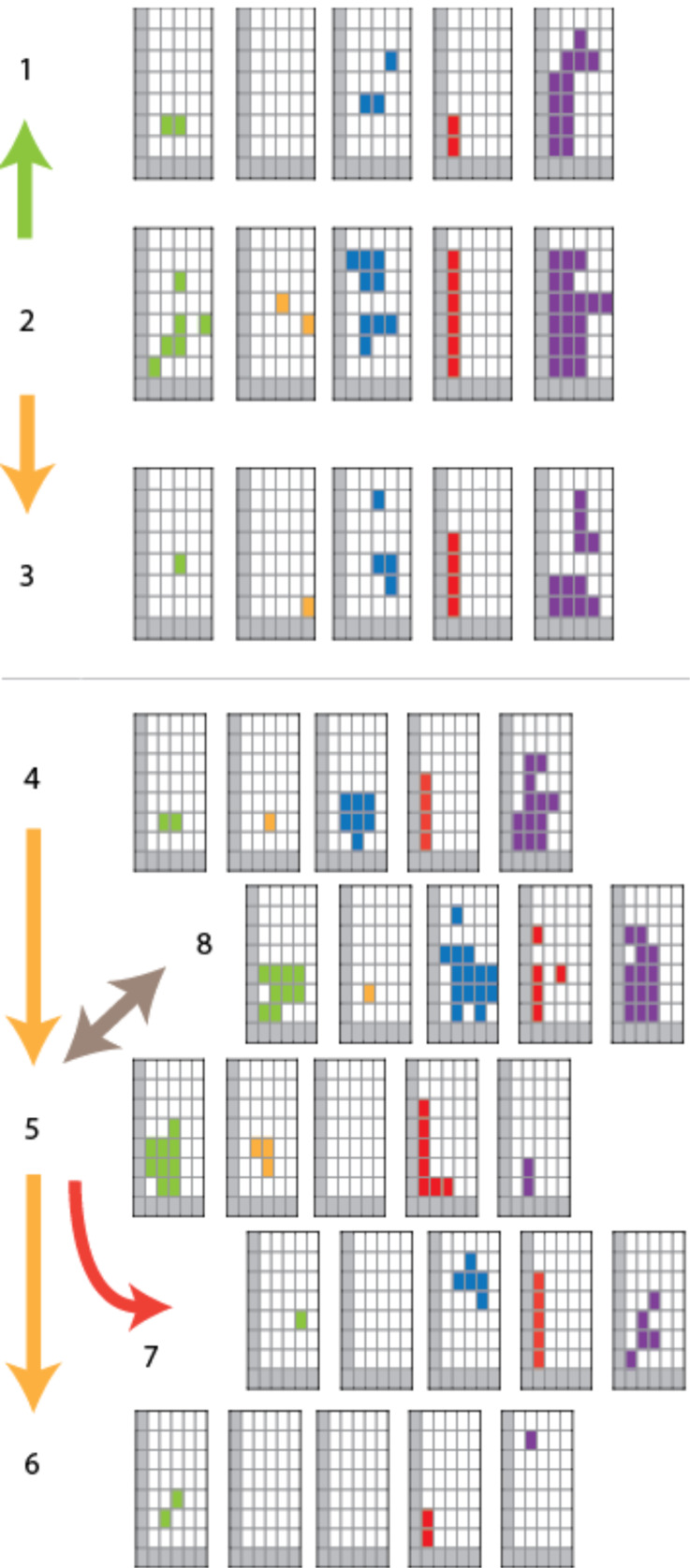
Single-molecule structure–activity relationships for compounds **1**–**8**. Grids are given in the following sequence in each case: Square-top (green); flicker (yellow); multiple-opening (blue); spike (red); erratic (purple). Yellow arrows indicate hydrocarbon homologs; green arrows indicate expansion of aromatic units; red arrows indicate the removal of a polar head group; the beige arrow indicates the reversal of chain polarity.

The second step involves a systematic inspection over several orders of magnitude in time and conductance in order to recognize and classify the conductance events that are encoded on the activity grid of each record. A single experiment consists of several records from a single day. Experiments were conducted over a period of time, with varying conditions of electrolyte, sequence of applied potentials, etc. The entire dataset of activity grids for a given compound was then summarized in a single set of grids. This procedure answers the question: Was a particular behavior of a particular conductance–duration ever observed? It does not provide information on the frequency of any particular type of activity or conductance–duration. This results in an activity profile for each compound, representative of the types of activity that were observed and their magnitude, from which a coarse-grained structure–activity can be mapped out (Figure 4).

The derived profiles are certainly consistent with our observations that almost every compound exhibits multiple types of activity. In this form it lacks a metric for the frequency of observation and is thus susceptible to both false positives (e.g., instrumental artifacts) as well as false negatives (i.e., absence of evidence is not evidence of absence). Nonetheless, the profiles reveal some interesting features when scrutinized through pairwise comparisons, and then from a “bird’s eye view”.

Specific pairwise comparisons are revealing in two senses. The first is that trends that were not obvious upon browsing through current–time traces stand out: For example, the comparison of the activity grids between the chain-reversed pair **5** and **8** shows that they are capable of forming “square-tops” of similar duration and conductance, but the latter also has other modes of irregular or erratic activity that are simply not observed in the former. The second is that, despite being able to point out empirical activity differences, these are almost impossible to rationalize in any simple way as the activities themselves are multifacetted and do not admit simple explanations.

In our systematic application of the “activity-grid” method to published records we found that, from the great number of unique architectures, there is an overall clustering characteristic for the type of activity [10]. What we find here is preliminary evidence that these relatively flexible molecules each individually also give rise to clusters of activity types. The extent of the clusters, as well as their magnitude in terms of duration and conductance, seem to be conserved between different linear bolaamphiphiles and correspond to the influence of each unique architecture on the overall system behavior. This is experimental support for the proposal [10] that there is some underlying energetic landscape that is as much a property of the system (lipid, water, and compound) as it is a property of the compounds we prepare and introduce.

### Voltage dependence

**Figure 5:**
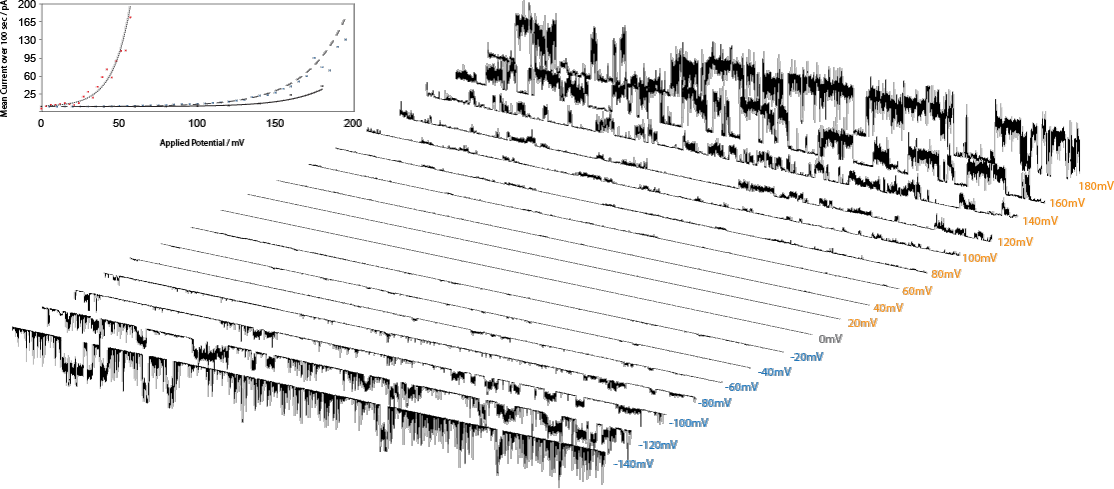
Voltage-dependent opening of compound **1**. Inset: Exponential dependence of the mean current on the potential. The solid line fits the trace shown in the figure.

Two types of voltage dependence have been reported previously. The first is a rectifying behavior, where ionic current passes preferentially for one polarity of the applied potential in comparison to the other polarity [20–21]. The second is a nonlinear current response to the applied potential, such as the exponential dependence seen in alamethicin [22–23] or in a synthetic channel [24]. Two of the compounds in the terephthalate series, **1** and **2**, exhibit voltage dependence in the second sense (Figure 5).

The current–time behavior is irregular, and therefore the current was averaged over a 200 second interval. A plot of average current as a function of applied potential appeared symmetrical about zero, so the two branches for a given experiment were combined as the average current as a function of the absolute applied potential. Fits to a single exponential were adequate given the erratic activity (r^2^ > 0.9), but both pre-exponential and exponential terms varied considerably between different experiments (Table 1). The exponent term of the fits can be used to derive an apparent gating charge, corresponding in an idealized model to the number of elementary charges required to move in response to the applied potential [25]. The observed values lie in the range from 1.2 to 3.5, with a cluster around 1.5. A physical interpretation of these values requires a detailed model, but it is clear that several charged species, presumed to be the protonated anilinium head groups, must move in response to the applied potential through some significant portion of the bilayer thickness. Neither the pre-exponential nor the exponent appears to be a characteristic solely of the compound. Concentration of the compound in the membrane presumably plays a large role, but the general lack of suitable solubility precludes additional studies.

**Table 1:** Voltage-dependent current (averaged over 200 s) for compounds **1** and **2**. **A** is the pre-exponential of the fitting, whereas **b** is the exponent.

Compound	**A**	**b**	r^2^	Apparent gating charge

**1**	0.0285	0.0645	0.9795	2.5
**1**	0.2062	0.0386	0.9671	1.5
**1**	0.4802	0.030	0.9702	1.2
**1**	0.0170	0.0422	0.9499	1.6
**2**	1.1919	0.0896	0.9199	3.5
**2**	0.0983	0.0382	0.9709	1.5
**2**	0.1655	0.032	0.9562	1.2

Qualitatively, both the open probability as well as unit conductance (of the somewhat regular “blue” multilevel openings) appears to be enhanced at higher potentials. What could be the underlying mechanism? One line of possibilities relates to the underlying “system property” mentioned earlier. It is known that pure lipids near their phase transitions can show single-channel conductance activities; while the specific mode of action depends on the identity of the lipid, discrete conductance events indistinguishable from channels are possible [26]. It was recently reported that an applied potential can effect lipid phase transitions [27], one consequence of which is the creation of voltage-dependent lipid ion channels at the phase-transition temperature. Pure diphytanoyl phosphatidylcholine is normally stable to potentials exceeding 400 mV and does not show any detectible phase transitions [28–29]. It is conceivable that our introduction of “impurities” creates local disorder akin to domain separation at the melting transition and that fluctuations at the domain boundaries would give rise to voltage-dependent channel activity.

## Conclusion

Two subclasses of linear oligoester bolaamphiphiles, comprising a total of eight compounds, were tested for conductance activity in diphytanoyl phosphatidylcholine planar bilayers. All compounds were active; most of them showed discrete “square-top” activity considered as a benchmark for the formation of single-channels. One of these compounds **5** has “square-top” activity as its predominant mode of activity, and two compounds in the terephthalate subclass show an exponential potential dependence of the ionic currents. Aside from the potential-dependent activity, the other types of conductance events can be documented in a standardized fashion by means of the “activity grid” method. From these individual profiles a (sparse) structure–activity map was constructed, and from this map two conclusions were drawn.

Firstly, the observations of clustered profiles match those that emerged from our systematic literature survey [10]. Note however, that the statistical support for this conclusion is very weak due to the very small number of observations (fewer than 200 on each side). Moreover, the irregular potential-dependence of the ion channels observed is similar in characteristic to the voltage-dependent lipid-only channels. These two parallels suggest that these simple bolaamphiphile compounds are not acting as channels on their own but rather in concert with a loosely defined “system” comprising lipid, compound, water, and electrolyte. A more focused investigation along these lines will be necessary to advance our understanding of how these simple channels “work”.

The second conclusion concerns the “what compounds ought we to think about” question. Many synthetic ion channels require heroic synthetic efforts, and one of the enduring motivations in various research groups is to find structures that are synthetically accessible such that structural variations are possible. This often translates into smaller “minimalist” channel molecules, and our suite of compounds was built along these lines. This makes sense only if there is a defined and simple activity, such as the observed “square-top” events. In this study, we provided evidence that, although regular activities are not rare, their presence does not exclude other types of activity. The frequent presence of other types of activity calls into question our ability to draw simple structure–function correlations. Rather, our attention should be focused on the entire range of different activities, and the activity profiles give us a means to do that. However, no obvious trend emerged from the pairwise comparisons of this study. One possible reason is that, as compounds become smaller, more individual molecules must come together for active self-interactions or interactions with the lipid, and site-specific changes are less able to target only a single molecular parameter (rather than a confounding host of them). If this is true, then it suggests that “as small as possible” is not the optimal size for future investigations, and a larger study involving species with higher molecular weights may thus lead to both more unique behaviors (with less dependence on “system” properties) and deeper structure–activity insights.

## Supporting Information

File 1 Synthesis procedures, spectroscopic characterization of new compounds and voltage-clamp summary activity records
