# Supplementary material for: Planar-bilayer activities of linear oligoester bolaamphiphiles
Source: Beilstein J Org Chem. 2011 Nov 22;7:1562–9. doi: 10.3762/bjoc.7.184 (PMC3252859; doi:10.3762/bjoc.7.184)
Supplement: File 1 — Synthesis procedures, spectroscopic characterization of new compounds and voltage-clamp summary activity records [file Beilstein_J_Org_Chem-07-1562-s001.pdf]

Supporting Information

for

Planar-bilayer activities of linear oligoester bolaamphiphiles

Jonathan K. W. Chui, Thomas M. Fyles* and Horace Luong§

Address: Department of Chemistry, University of Victoria, Victoria BC, Canada

Email: Thomas M. Fyles* - tmf@uvic.ca; Jonathan K. W. Chui - jkwchui@uvic.ca

* Corresponding author

§ Current address: Department of Chemistry, University of Manitoba, Winnipeg MB, Canada

Synthesis procedures, spectroscopic characterization of new compounds and voltage-clamp summary activity records

General information

Reagents and general chemicals were purchased from Aldrich. Unless otherwise specified, all solvents were used as supplied without further purification. Analytical thin-layer chromatography (TLC) was performed on E. Merck aluminium-backed silica gel (Silica Gel F254); compounds were identified by charring with a solution of *p*-anisaldehyde in aqueous sulfuric acid and ethanol. NMR spectra were recorded with either (i) a Bruker AMX spectrometer operating at 300 MHz for ^{1}H nuclei, 75 MHz for ^{13}C nuclei, and 282 MHz for ^{19}F nuclei, or (ii) a Bruker AMX spectrometer operating at 500 MHz for ^{1}H nuclei, and 126 MHz for ^{13}C nuclei. Low resolution mass spectra (accurate to 10^{-1} amu) were recorded with a Q-TOF II (MicroMass/Waters, Milford MA) with 4000 *m*/*z* max quadrupole. Samples were prepared as 1 mg/mL solutions in methanol:water, and diluted by a factor of 10, followed by 0.1% trifluoroacetic acid which was added to generate more ions. High resolution mass spectra (accurate to 0.5 ppm) were obtained on an LTQ Orbitrap Velos from Thermo Scientific with

200–2000 mass range and 300 nL/min liquid infusion. Samples were prepared as 10 ng/μL solutions in methanol.

The syntheses of compounds **4**–**8** were previously reported [1]. *t*-BOC masking of *p*-aminobenzoic acid was achieved by procedures previously described [2].

Synthesis of 9

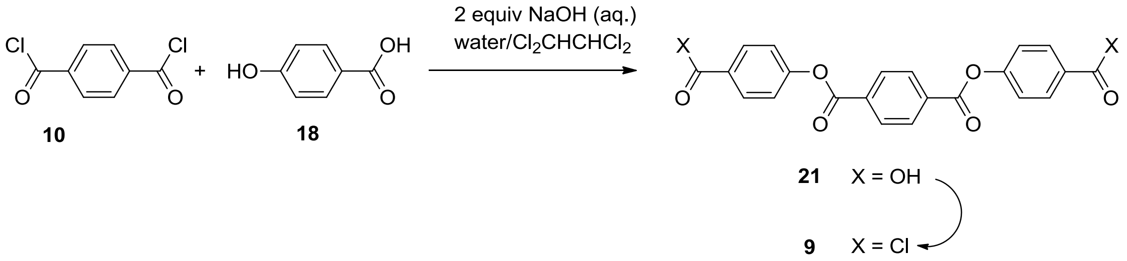

4-Hydroxybenzoic acid was weighed out as a solid (5.78 g, 41.9 mmol, 3.0 equiv) in a 250 mL beaker and dissolved in 58 mL of 1 M NaOH (4.1 equiv) to give a clear, homogenous solution. This was then transferred to a two-neck 250 mL round-bottom flask and cooled to 5 °C in an ice bath. Terephthaloyl chloride (2.89 g, 14.2 mmol, 1.0 equiv) was dissolved in $Cl_2CHCHCl_2$ (30 mL), and poured into a dropping funnel attached to the reaction flask. The solution was added dropwise over 1 h while stirring rapidly. The reaction mixture became white and heterogeneous and was stirred at rt. After 4 h, the reaction was quenched by acidifying to pH2 with concentrated HCl, and then filtered. The precipitate was washed sequentially with water, Et_2O, and EtOH before being dried at 60 °C overnight. Starting material 4-hydroxybenzoic acid was shown to be present by ^{1}H NMR spectroscopy, and was removed by grinding the solid in acetone and then refiltered, yielding 5.3 g of a white powder (92%; compound **21**). The product was sparingly soluble in common organic solvents and was purified only as the acid chloride.

To convert the bis-acid **21** to the bis-acyl chloride **9**, the acid (0.70 g, 1.7 mmol, 1.0 equiv) was refluxed for 4 h in 9 mL of $SOCl_2$ to give a yellow slurry. Excess $SOCl_2$ was removed under vacuum, and the product was washed with Et_2O to give 763 mg of **9** as white flakes (87% yield). This material is very moisture-sensitive and should be used immediately after its isolation. IR (KBR): 1782, 1741, 1598, 1497, 1409, 1263, 1214, 1165, 1076, 1014, 886, 816, 725, 712, 642 cm^{-1}. ^{1}H NMR (250 MHz, $CDCl_3$) δ 8.37 (s, 4H), 8.2 (d, *J* = 8, 4H), 7.41 (d, *J* = 8, 4H) ppm.

Coupling of membrane anchor to the triaromatic core 9

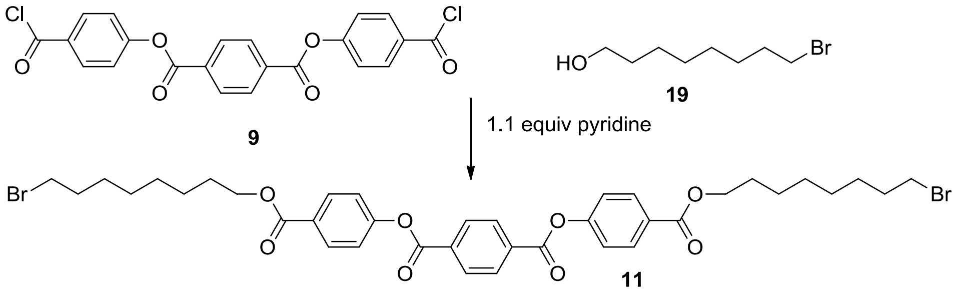

The general procedure is illustrated with compound **11**. A 50 mL round-bottom flask equipped with a stirring bar was flame dried. Upon cooling under a N_2 atmosphere, acyl chloride **9** (211 mg, 0.476 mmol, 1.0 equiv) was suspended in 8 mL of dry CH_2Cl_2. This heterogeneous solution was cooled in an alcohol–ice bath to −4 °C before 8-bromo-1-octanol (199 mg, 0.952 mmol, 2.0 equiv) and 69 μL pyridine (0.95 mmol, 2.0 equiv) were added in that order. The reaction was stirred at 0 °C for 1 h and then allowed to warm to rt. The reaction was diluted with 25 mL of CH_2Cl_2 and washed with brine. The organic solution was dried over $MgSO_4$, filtered, and concentrated. Column chromatography (silica gel, with 1:4 EtOAc:hexanes as eluent) yielded 149 mg of a white powder (40%). ESI–MS (*m*/*z*) calculated for $[C_{38}H_{44}Br_2O_8+Na]^+$ 809.13; found 809.25; ^{1}H NMR (300 MHz, $CDCl_3$) δ 8.28 (s, 4H), 8.08 (d, *J* = 8.9, 4H), 7.27 (d, *J* = 8.9, 4H), 4.27 (t, *J* = 6.6, 4H), 3.35 (t, *J* = 6.8, 4H), 1.82–1.68 (m, 8H), 1.39–1.30 (m, 16H); ^{13}C NMR (75 MHz, $CDCl_3$) δ 163.5, 161.4, 152.1, 131.4, 128.9, 128.3, 126.2, 119.4, 62.8, 31.8, 30.4, 26.9, 26.51, 26.41, 25.7, 23.6.

Esterification by iodide-catalyzed nucleophilic displacement

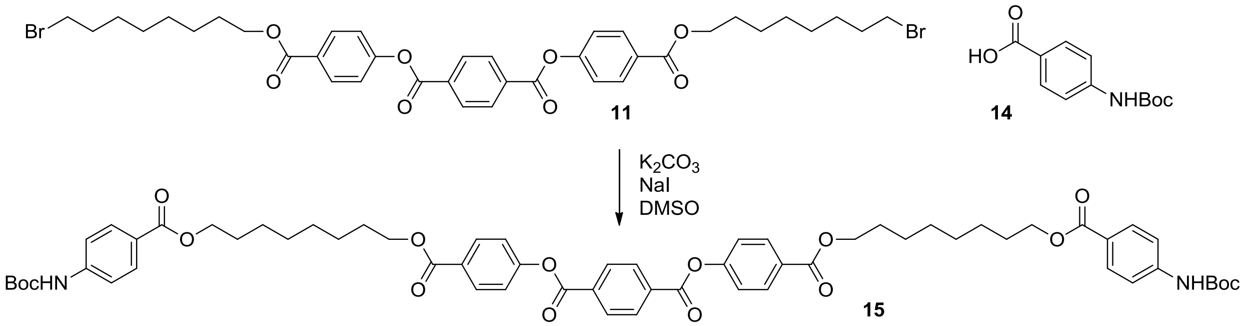

The general procedure is illustrated with compound **15**. In a 50 mL round-bottom flask equipped with a stirring bar, dibromide **11** (646 mg, 0.819 mmol, 1.0 equiv), protected aniline **14** (447 mg, 1.884 mmol, 2.3 equiv), and sodium iodide (25 mg, 0.164 mmol, 0.2 equiv) were dissolved in DMSO with vigorous stirring. Tetramethylammonium hydroxide (341 mg, 1.884 mmol, 2.3 equiv) was then added, and the cloudy white solution was heated to 60 °C. The reaction was shown by TLC (silica gel, 1:1 EtOAc:hexanes as eluent, UV-254 nm detection) to be complete in 2 h (disappearance of compound **11** at R_f = 0.80). Upon completion of the reaction, the reaction mixture was diluted with 30 mL CH_2Cl_2 and washed with 80 mL water. The aqueous phase was backwashed with 25 mL of CH_2Cl_2, and the combined organic layers were dried over $MgSO_4$ and filtered to give a clear colorless solution. The solvent was evaporated and solids crystallized from 20 mL of hot CH_2Cl_2-EtOAc to give 740 mg of **15** as a white powder (82%). ESI–MS (*m*/*z*) calculated for $[C_{62}H_{72}N_2O_{16}+Na]^+$ = 1123.48; found 1123.51; ^{1}H NMR (300 MHz, $CDCl_3$) δ 8.28 (s, 4H), 8.08 (d, *J* = 8.8, 4H), 7.89 (d, *J* = 8.8, 4H), 7.35 (d, *J* = 8.8, 4H), 7.26 (d, *J* = 8.8, 4H), 6.70 (s, 2H), 4.30–4.18 (m, 8H), 1.75–1.63 (m, 8H), 1.45 (s, 18H), 1.40–1.34 (m, 16H); ^{13}C NMR (75 MHz, $CDCl_3$) δ 166.3, 165.9, 163.8, 154.3, 152.2, 142.7, 133.8, 131.4, 130.9, 130.5, 128.5, 124.6, 121.7, 117.3, 81.3, 65.4, 64.9, 29.2, 28.8, 28.3, 26.1.

Compound 1 by deprotection of 15

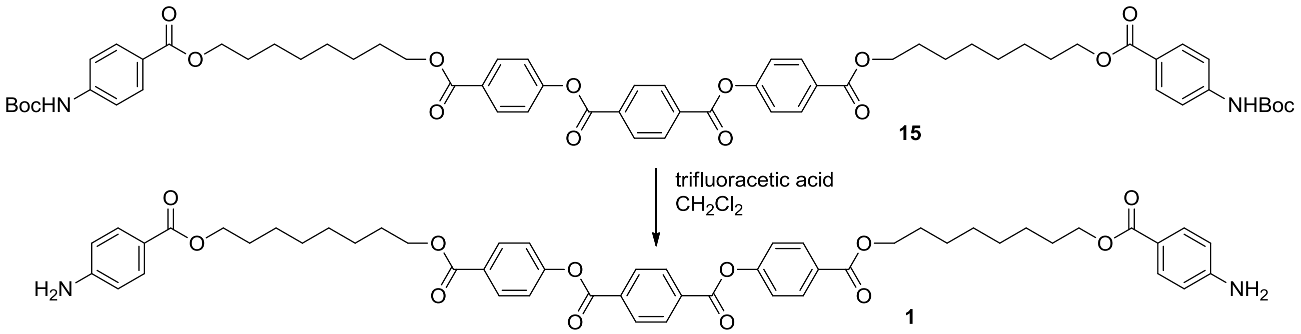

The ester-linked tri-aromatic core is acid sensitive, and this cleavage condition [3] of the *tert*-butyl carbamate was necessary to preserve the core. The reagent was prepared in a 10 mL batch beforehand: Trimethylsilyl chloride (1.09 g, 10 mmol) was weighed into a 10 mL graduated cylinder and diluted to the 5 mL mark with CH_2Cl_2. Phenol (2.82 g, 30 mmol) was added as a solid, and the solution diluted to the 10 mL mark. In a flame-dried 5 mL round-bottom flask

equipped with a stirring bar, protected aniline **24** (95 mg, 0.086 mmol) was added. To this solid, 2.5 mL of the cleavage reagent (described above) was added, upon which a homogenous solution was obtained. The flask was then capped, sealed with Teflon tape, and stirred for 14 h. The reaction slowly turned cloudy over time. The reaction was quenched by addition of an aqueous solution of saturated K_2CO_3; the pH of this solution was around 12 at this point. Repeated extractions with chloroform, followed by drying of the organic layer over $MgSO_4$, filtering, and drying gives a white solid. Column chromatography (silica gel, 1% MeOH in chloroform) gave the product contaminated with phenol. This material was dissolved in 3 mL of chloroform and precipitated by 25 mL of ice-cold hexanes, followed by filtration giving 40 mg of **1** as a white powder (51%). HRMS (*m*/*z*) calculated for $[C_{52}H_{56}N_2O_{12}+H]^+$ = 901.3908; found 901.3899; ^{1}H NMR (300 MHz, $CDCl_3$) δ 8.33 (s, 4H), 8.13 (d, *J* = 8.9, 4H), 7.83 (d, *J* = 8.8, 4H), 7.32 (d, *J* = 8.8, 4H), 6.61 (d, *J* = 8.8, 4H), 4.32 (t, *J* = 6.7, 4H), 4.24 (t, J = 6.5, 4H), 4.00 (s,br, 4H), 1.77–1.70 (m, 7H), 1.43–1.23 (m, 27H); ^{13}C NMR (126 MHz, $CDCl_3$) δ 131.8, 131.5, 130.7, 121.8, 114.0, 65.6, 64.7, 29.4, 29.04, 28.95, 26.27, 26.22. Insufficient amount of sample to observe signals from quaternary carbons.

Coupling of membrane anchors to the monoaromatic core

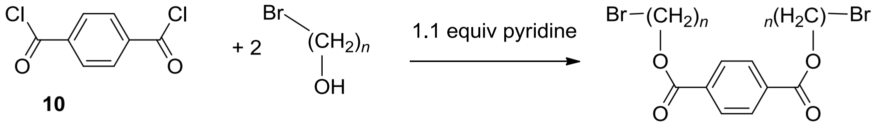

10

19 *n* = 8

20 *n* = 12

12 *n* = 8

13 *n* = 12

The general procedure is illustrated with compound **13**. In a flame-dried 25 mL round-bottom flask, terephthaloyl chloride (250 mg, 1.23 mmol, 1.0 equiv) was dissolved in 5 mL of dry CH_2Cl_2. 12-Bromo-1-dodecanol (650 mg, 2.46 mmol, 2.0 equiv) was added, and the solution cooled in an ice-bath. Pyridine (198 μL, 2.46 mmol, 2.0 equiv) was then added by syringe, and the solution became slightly cloudy over 10 min. The reaction was allowed to warm to rt and stirred for 2 h. To quench the reaction, it was first diluted with 20 mL of CH_2Cl_2 before the addition of 30 mL of water. The aqueous portion was washed twice with 20 mL of CH_2Cl_2, and the combined organic layers were dried over $MgSO_4$, filtered, and dried. This solid was

dissolved in a small volume of EtOAc for loading onto a column; column chromatography (silica gel, using 1:9 EtOAc:hexanes as eluent) gave 666 mg of **13** as a white powder (87%).

Characterization for **12**. ESI–MS (*m*/*z*) calculated for $[C_{24}H_{36}Br_2O_4+H]^+$ = 549.10; found 550.71 $([M+H]^+)$. ^{1}H NMR (300 MHz, $CDCl_3$) δ 8.03 (s, 4H), 4.27 (t, *J* = 6.7, 4H), 3.33 (t, *J* = 6.9, 4H), 1.83–1.68 (m, *J* = 14.2, 7.2, 8H), 1.37–1.28 (m, 12H); ^{13}C NMR (75 MHz, $CDCl_3$) δ 165.0, 133.3, 128.6, 64.6, 33.2, 28.70, 28.59, 28.40, 27.96, 27.86, 27.3.

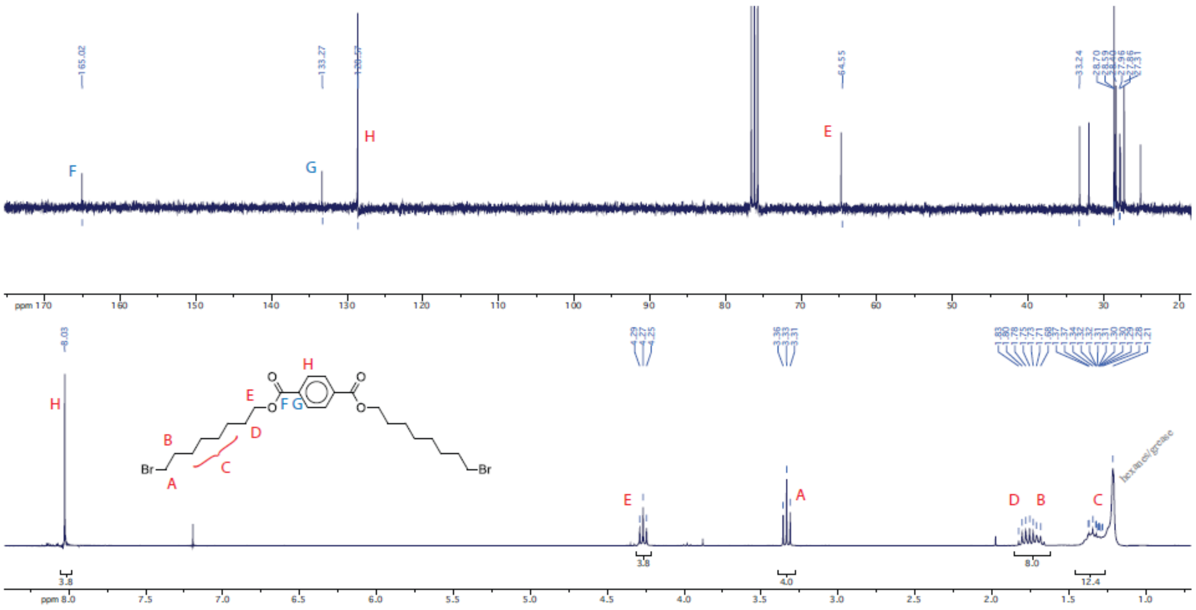

Figure S1: NMR spectra of **12**.

Characterization for **13**. ESI–MS (*m*/*z*) calculated for $[C_{32}H_{52}Br_2O_4+H]^+$ = 661.229; found 661.32 ($[M+H]^+$). ^1H NMR (300 MHz, $CDCl_3$) δ 8.02 (s, 4H), 4.26 (t, *J* = 6.7, 4H), 3.33 (t, *J* = 6.9, 4H), 1.80–1.70 (m, *J* = 7.0, 8H), 1.37–1.21 (m, 32H); ^{13}C NMR (126 MHz, $CDCl_3$) δ 165.9, 134.2, 129.5, 65.6, 34.0, 32.8, 29.49, 29.41, 29.25, 28.75, 28.65, 28.2, 26.0.

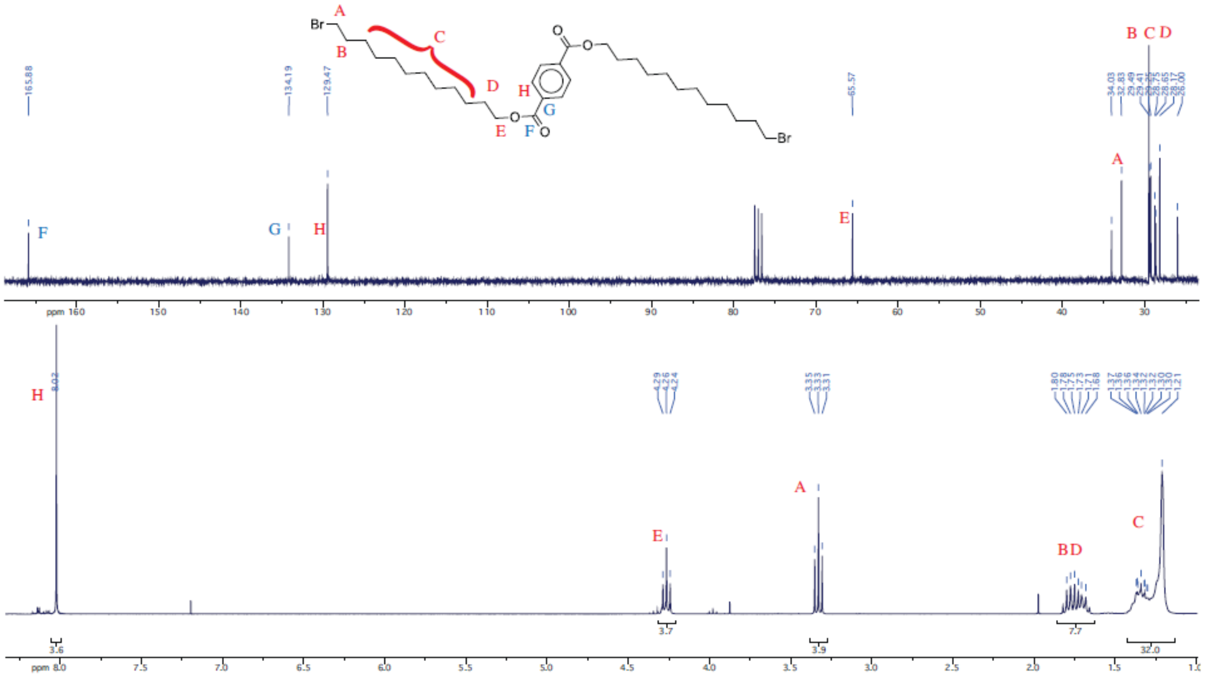

Figure S2: NMR spectra of **13**.

Iodide-catalyzed nucleophilic displacement to give 16 and 17

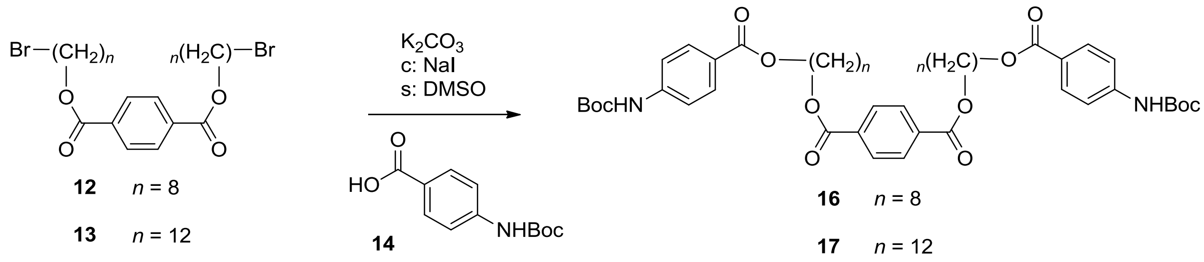

General procedure illustrated with compound **16**. In a 10 mL round-bottom flask, dibromide **12** (503 mg, 0.937 mmol, 1.0 equiv), OC-protected *p*-aminocarboxylic acid **14** (478 mg, 2.02 mmol, 2.2 equiv), and NaI (28 mg, 0.19 mmol, 0.2 equiv) was dissolved in DMSO. Tetramethylammonium hydroxide (367 mg, 2.02 mmol, 2.2 equiv) was added, and the reaction was stirred at 70 °C for 1 h, after which TLC (silica gel, 1:1 EtOAc:hexanes, UV-254 nm visualization) indicated that the reaction was completed. The reaction was quenched in 120 mL of distilled water, extracted thrice with 50 mL of chloroform. (Addition of brine was necessary in some cases for clear separation of the organic and aqueous layers.) The combined organic layers were washed with 30 mL water, dried over $MgSO_4$, filtered, and evaporated to afford a yellow oil. This yellow oil was chromatographed (silica gel, with chloroform as eluent) to give 593 mg of a white solid (66%).

Characterization for **16**. ESI–MS (*m*/*z*) calculated for $[C_{48}H_{64}N_2O_{12}+Na]^+$ = 883.4358; found 883.6. ^{1}H NMR (300 MHz, $CDCl_3$) δ 8.02 (s, 4H), 7.89 (d, *J* = 8.8, 4H), 7.36 (d, *J* = 8.8, 4H), 6.71 (s, 2H), 4.26 (t, *J* = 6.7, 4H), 4.21 (t, *J* = 6.7, 4H), 1.73–1.66 (m, 8H), 1.45 (s, 18H), 1.36–1.18 (m, 16H); ^{13}C NMR (126 MHz, $CDCl_3$) δ 166.4, 166.0, 152.2, 142.7, 134.1, 130.8, 129.5, 124.6, 117.3, 81.2, 65.6, 64.9, 29.2, 28.80, 28.68, 28.3, 26.1.

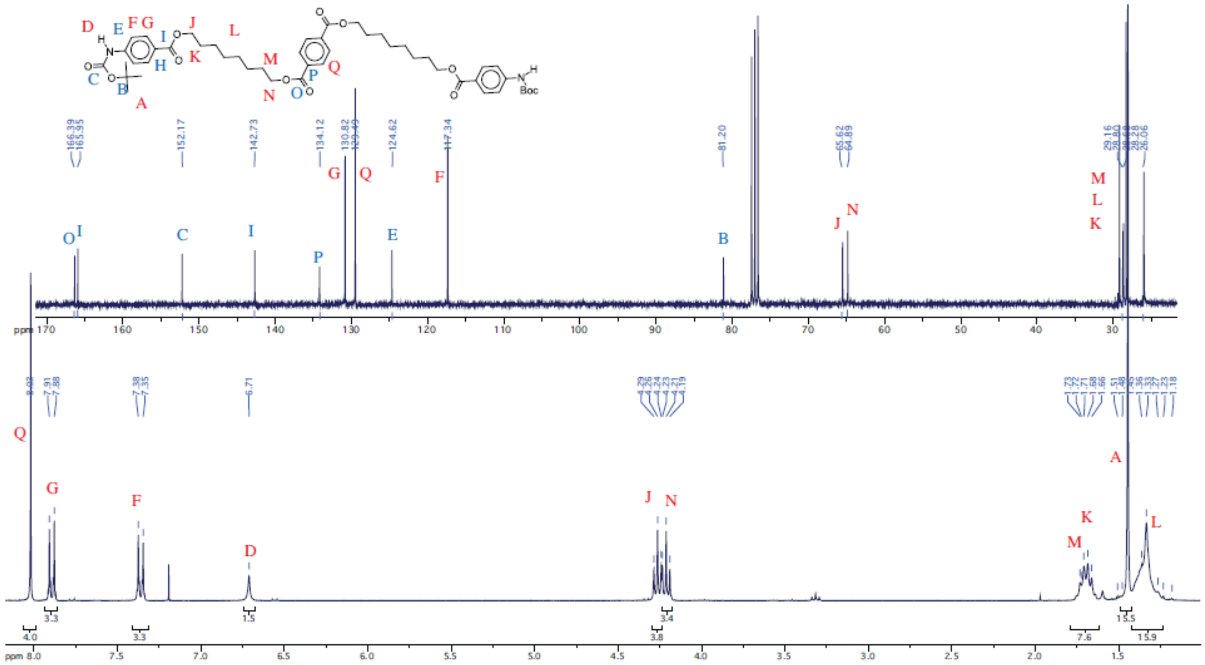

Figure S3: NMR spectra of **16**.

Characterization for **17**. ^{1}H NMR (300 MHz, CDCl3) δ 8.02 (s, 4H), 7.89 (d, *J* = 8.8, 4H), 7.36 (d, *J* = 8.8, 4H), 6.71 (s, 2H), 4.26 (t, *J* = 6.7, 4H), 4.21 (t, *J* = 6.7, 4H), 1.73–1.66 (m, 8H), 1.45 (s, 18H), 1.36–1.18 (m, 32H).

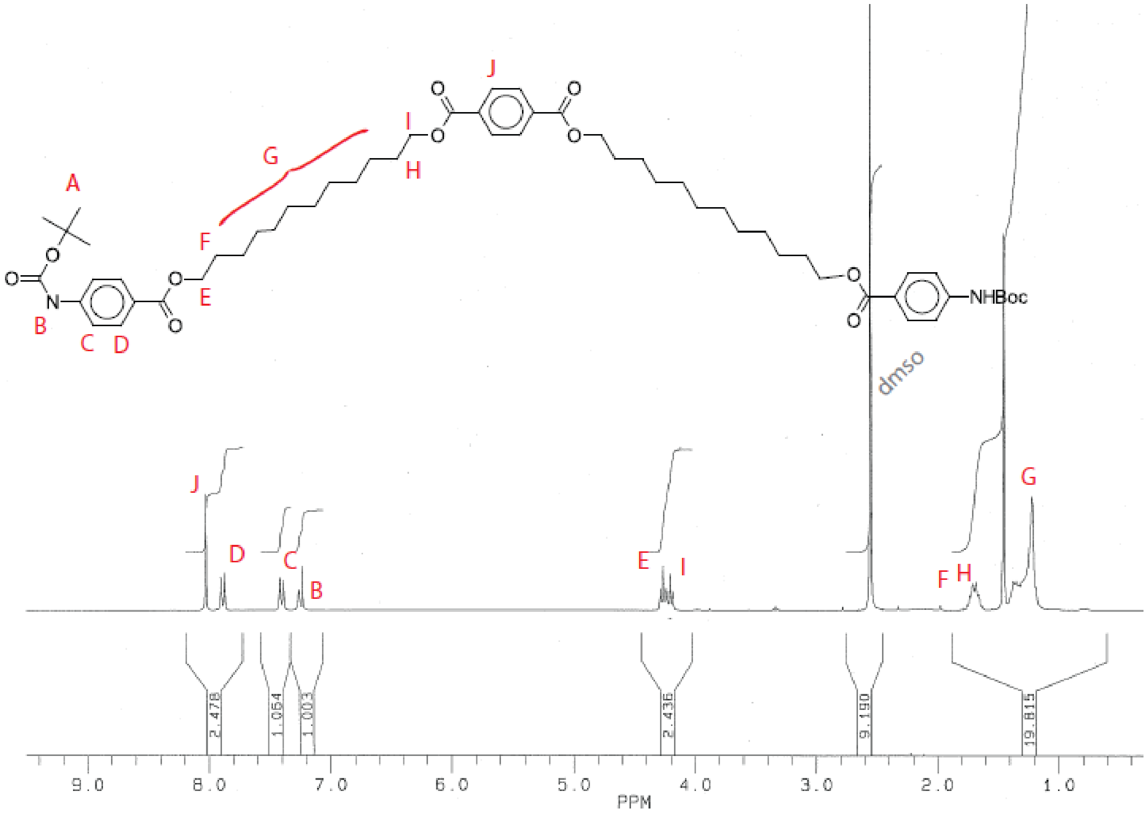

Figure S4: ^{1}H NMR spectrum of **17.**

Deprotection to give bolaamphiphiles 2 and 3

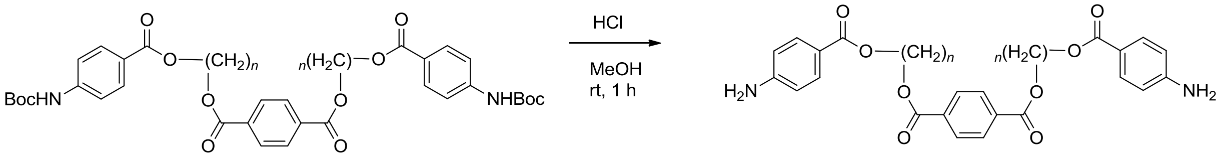

16 $n = 8$

17 $n = 12$

2 $n = 8$

3 $n = 12$

Deprotection of the suite of mono-aromatic compounds proceeded with the same TMSCl/phenol reagent as reported above for the deprotection of compound **1**.

Characterization for **2**. HRMS (*m*/*z*) calculated for $[C_{38}H_{48}N_2O_8+H]^+$ = 661.349; found 661.3485. ^{1}H NMR (300 MHz, $CDCl_3$) δ 8.02 (s, 4H), 7.80–7.75 (m, 4H), 6.58–6.54 (m, 4H), 4.26 (t, *J* = 6.6, 4H), 4.18 (t, *J* = 6.6, 4H), 3.97 (s, br, 4H), 1.71–1.67 (m, 8H), 1.39–1.33 (m, 16H); ^{13}C NMR (126 MHz, $CDCl_3$) δ 167.0, 165.5, 150.0, 134.7, 131.5, 129.4, 119.7, 113.7, 66.2, 64.4, 29.3, 28.1, 26.4.

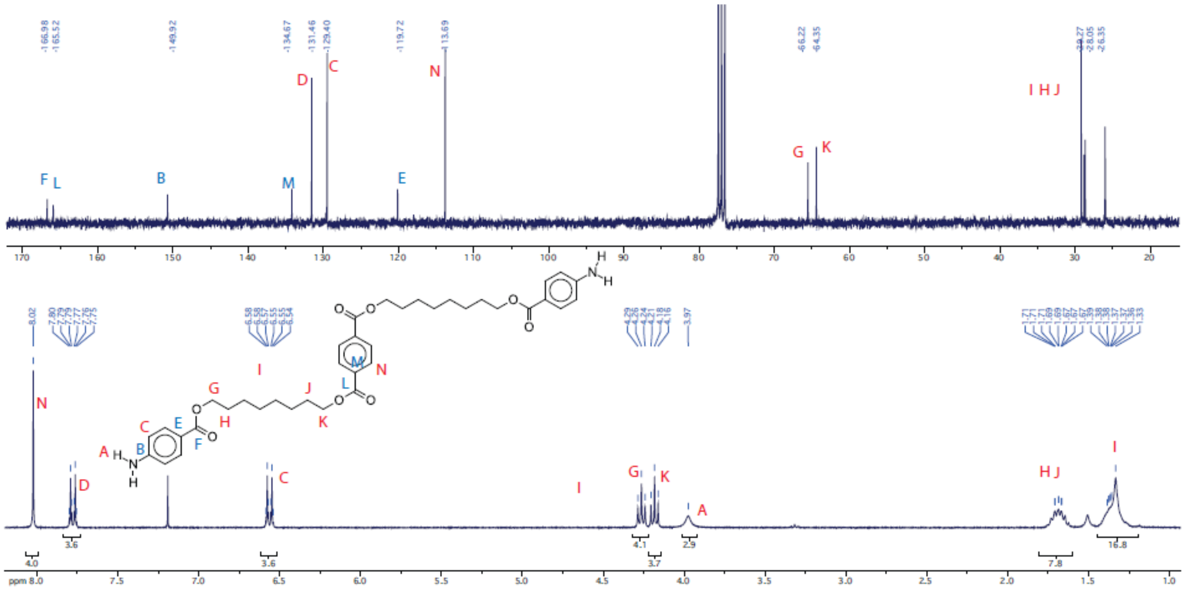

Figure S5: NMR spectra of **2**.

Characterization for **3**. HRMS (*m*/*z*) calculated for $[C_{46}H_{64}N_2O_8+H]^+$ = 773.474; found 773.4720. ^{1}H NMR (300 MHz, $CDCl_3$) δ 8.03 (s, 4H), 7.78 (d, *J* = 8.8, 4H), 6.57 (d, *J* = 8.8, 4H), 4.26 (t, *J* = 6.7, 4H), 4.18 (t, *J* = 6.7, 4H), 3.98 (s,br, 4H), 1.75–1.61 (m, 8H), 1.37–1.22 (m, 32H); ^{13}C NMR (75 MHz; $CDCl_3$) δ 166.8, 165.9, 150.8, 134.2, 131.6, 129.6, 120.1, 113.8, 65.7, 64.5, 29.6, 29.3, 28.9, 28.6, 26.13, 25.96.

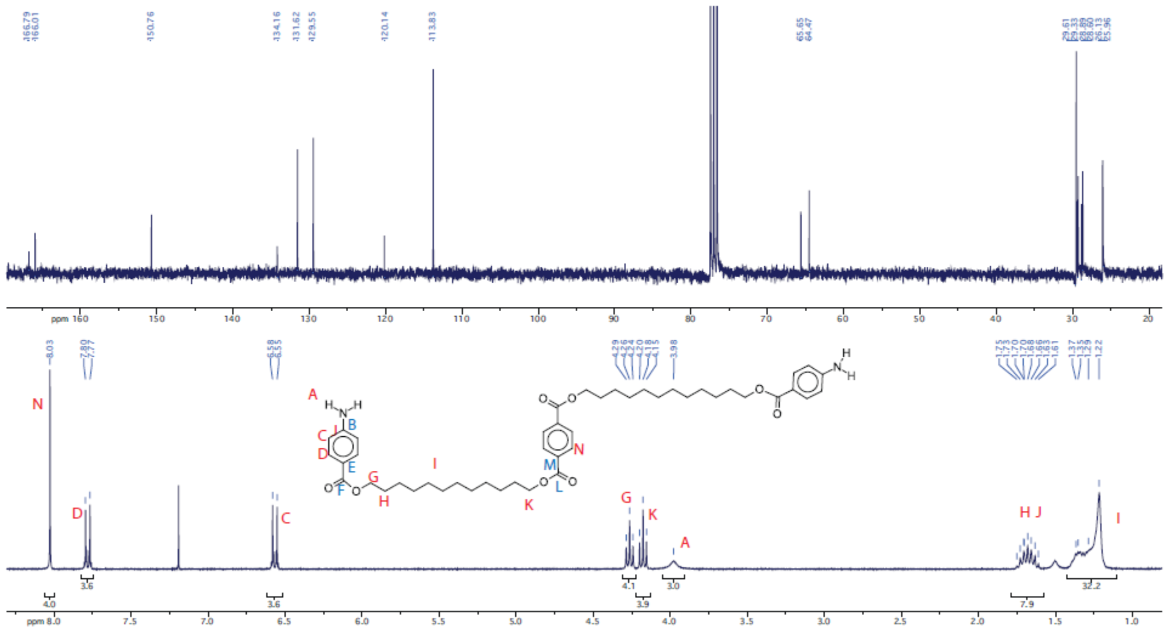

Figure S5: NMR spectra of **3**.

Voltage-clamp experiment

A model BC-525A bilayer clamp (Warner Instrument Corp.) was used for planar bilayer experiments. The analogue output was filtered with an 8-pole bessel filter (Frequency Devices, model 902) and digitized with a 333 kHz digitizer (Axon Instruments, Digidata 1200A). Data acquisition was controlled by the pClamp8 software package (Axon Instruments). Data were collected at 10 kHz, analogue filtered at 1 kHz, and digitally filtered at 50 Hz. The headstage and the bilayer chamber (3 mL polystyrene cuvette with 250 μm diameter aperture held in a 5 mL PVC holder) were placed on a floating table and electrically shielded by a grounded aluminum Faraday cage. Agar salt bridges (2 M KNO_3 in 1% Agar) were used to stabilize junction potentials and were employed between the electrolyte in each well of the cell and Ag/AgCl electrodes. Electrolyte solutions were prepared from high purity salts and nanopure water.

A stock solution of diphytanoyl phosphatidylcholine (diPhyPC) in chloroform (Avanti Polar Lipids; shipped on dry ice) was divided into sealed glass vials under an argon atmosphere and stored at −12 °C. For use in an experiment, a stream of dry nitrogen was passed through the vial for 1 h. The dried lipid was diluted with decane to give a solution concentration of 25 mg/mL lipid.

Bilayers were formed by either brushing or dipping: After lipid in decane was introduced by brushing, a lipid/decane film formed on the surface of the electrolyte, and bilayers could then be formed by withdrawal of 2–3 mL of electrolyte from the cell holder by syringe to expose one face of the aperture to the air–water interface held in the cell holder, followed by reintroduction of the electrolyte to oppose monolayers across the aperture of the cuvette. Bilayer quality was monitored through the capacitance and stability under the applied potential, with respect to the criteria previously described [4]. The measured voltage was applied with respect to the trans (cuvette) side of the bilayer, making the trans side the relative ground. Digitized data files were analyzed with the pClamp10 suite of programs.

The compounds are introduced to the membrane in different ways depending on the solubility of the compound. Solid-phase derived oligoesters were introduced by the electrolyte-mixing method, where compounds were premixed into a small volume of electrolyte, and this homogenous doped electrolyte solution was introduced to the bilayer. With the direct-injection method, aliquots (1–5 μL) of transporter solutions in MeOH were injected with a microliter syringe as close as possible to the bilayer in the free well of the cuvette holder (cis side), and gently stirred with a stream of nitrogen for 5 min. Experiments with both the electrolyte-mixing or direct-injection methods utilized bilayers that were apparently stable at 100 mV for periods of 20 min or more. In the physical transfer method, 1 mol % of compound (in $CDCl_3$ or MeOH-d_4) was added to the diPhyPC/$CHCl_3$ solution, the solvent removed with a stream of N_2, and the bilayer membrane prepared by brushing/dipping as described above. Most of the bilayers formed with this method gave bilayers with good quality.

Voltage-clamp records of linear bolaamphiphiles

In the following sections, annotated activity grids, as well as full conductance records (and expansions where appropriate), are provided for every compound studied. These are arranged

first by compounds, then individual experiments. Within each experiment, the first page(s) summarizes the experimental conditions as well as activity grids charted; subsequent pages show the full conductance record as the top panel, with expansions indicated by the corresponding letters. For the aromatic bolaamphiphiles, traces were acquired prior to establishing a protocol for the automated acquisition of potential—time records, and the data analysis were performed by hand.

References

1. Luong, H.; Fyles, T. M. *Org. Biomol. Chem.* **2009,** *7,* 725–732. doi:10.1039/B816648J
2. Norman, M. H.; Rigdon, G. C.; Hall, W. R.; Navas, F. *J. Med. Chem.* **1996,** *39,* 1172–1188. doi:10.1021/jm950551d
3. Kaiser, E., Sr.; Picart, F.; Kubiak, T.; Tam, J. P.; Merrifield, R. B. *J. Org. Chem.* **1993,** *58,* 5167–5175. doi:10.1021/jo00071a028
4. Fyles, T. M.; Knoy, R.; Müllen, K.; Sieffert, M. *Langmuir* **2001,** *17,* 6669–6674. doi:10.1021/la0105937

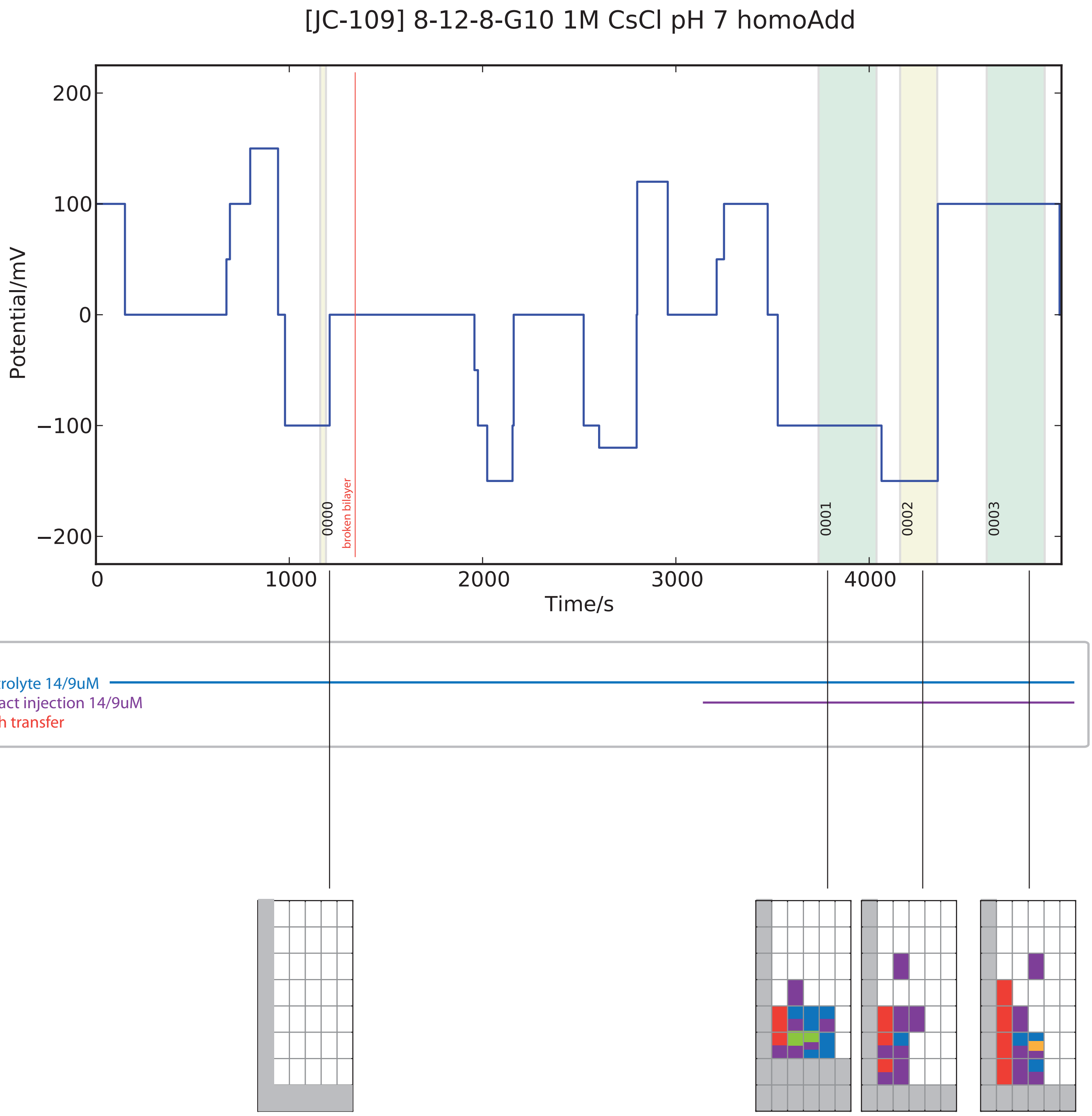

baseline drift 10-18pS;
no activity otherwise

109-0000

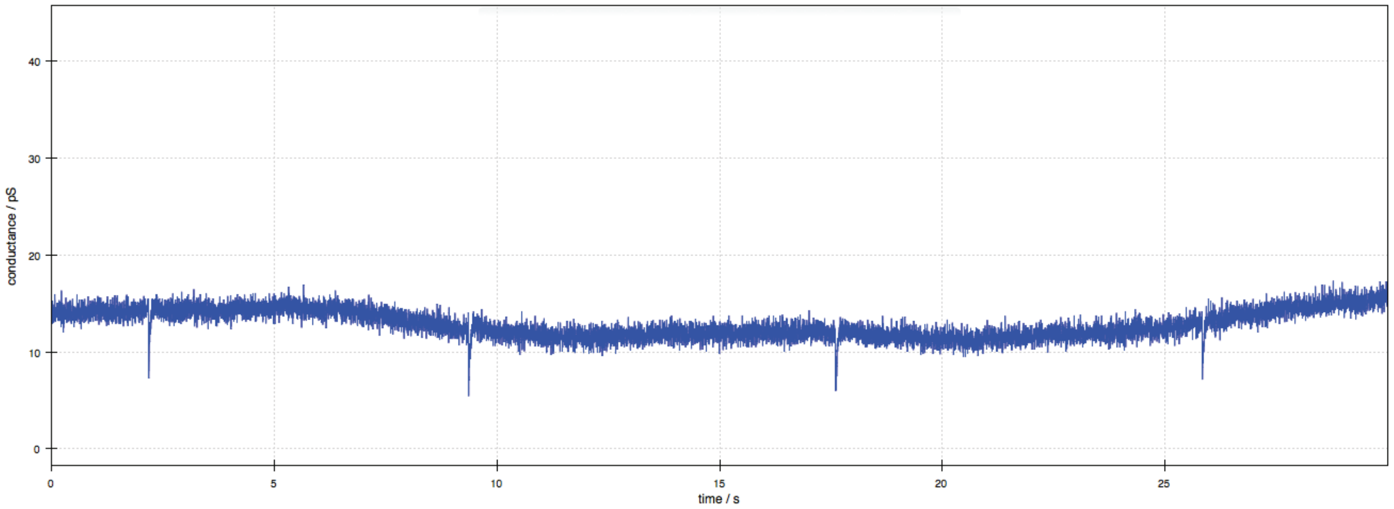

109-0001

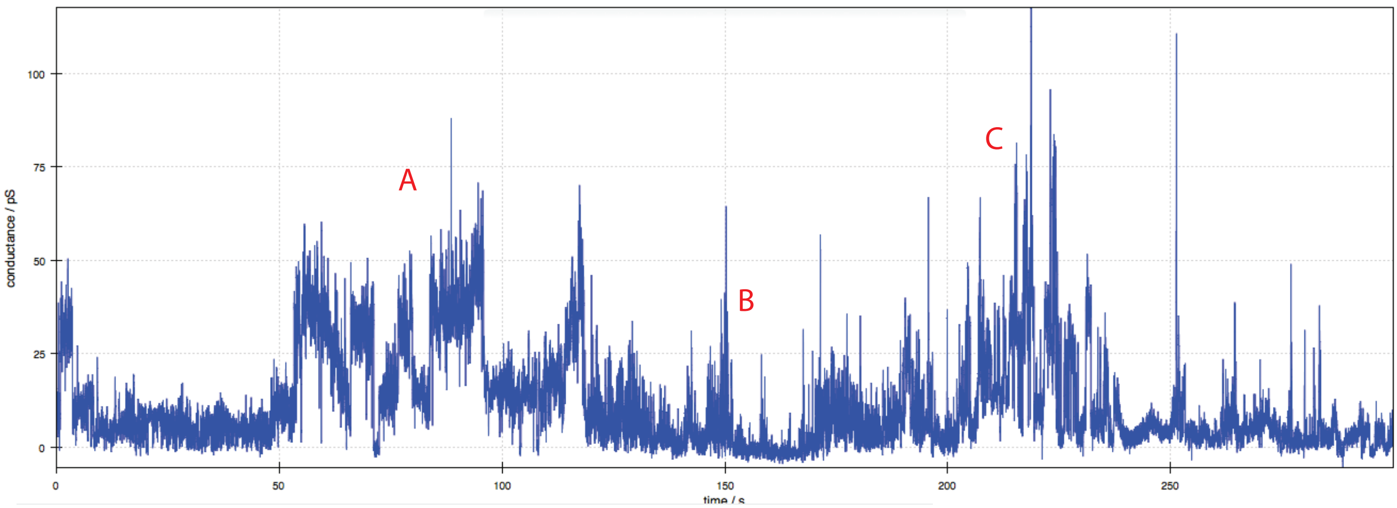

conductance / pS
time / s
A
B
C

A

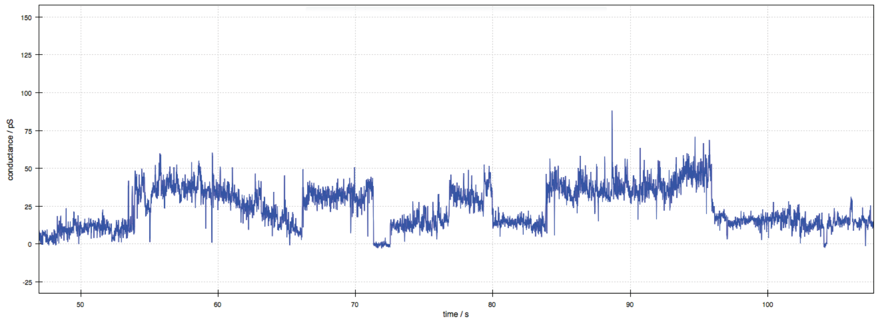

conductance / pS
time / s

B

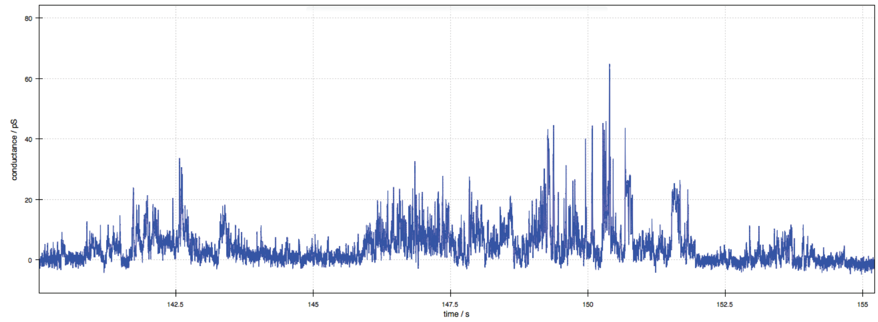

conductance / pS
time / s

C

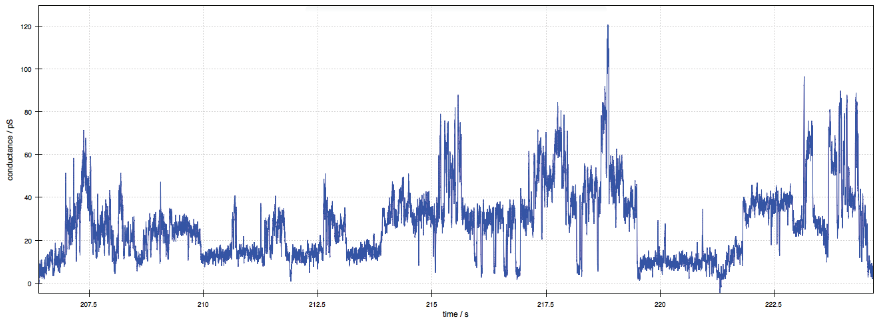

conductance / pS
time / s

109-0002

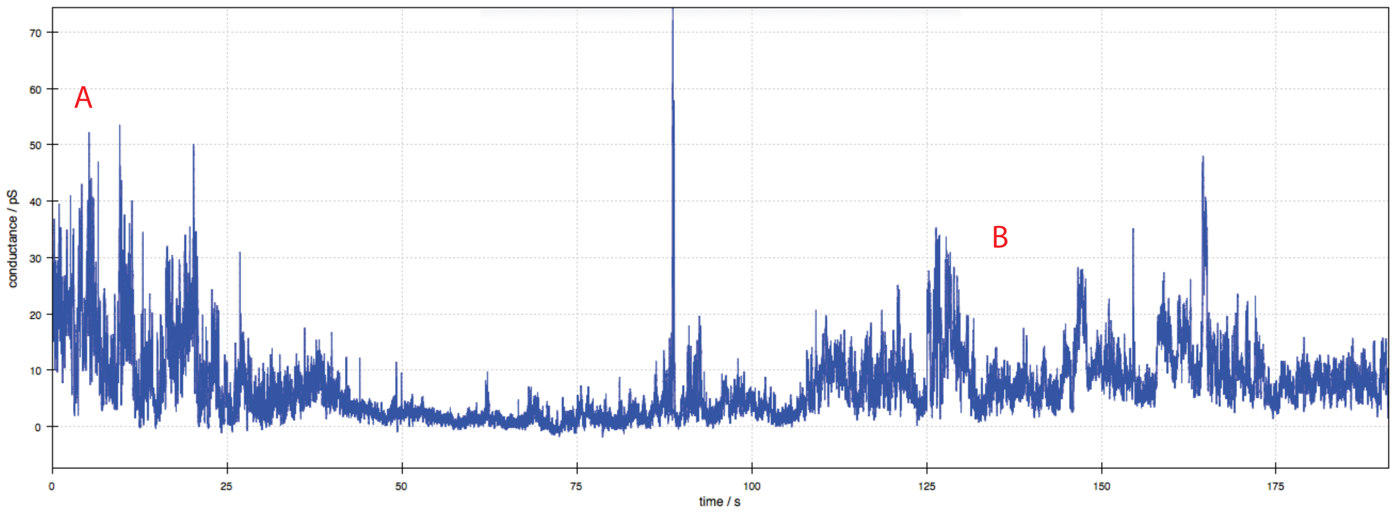

A
B
conductance / pS
time / s

A

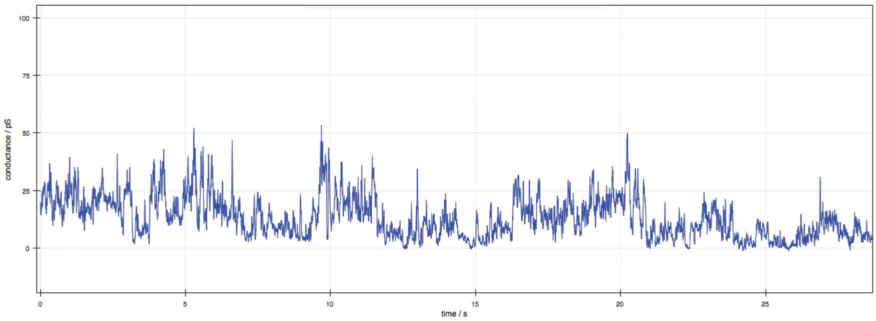

conductance / pS
time / s

B

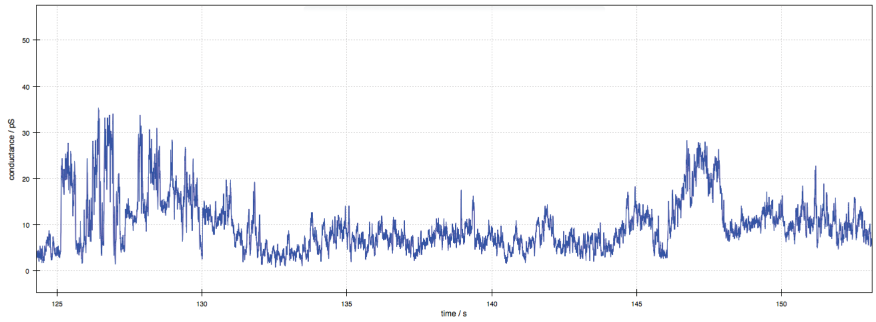

conductance / pS
time / s

109-0003

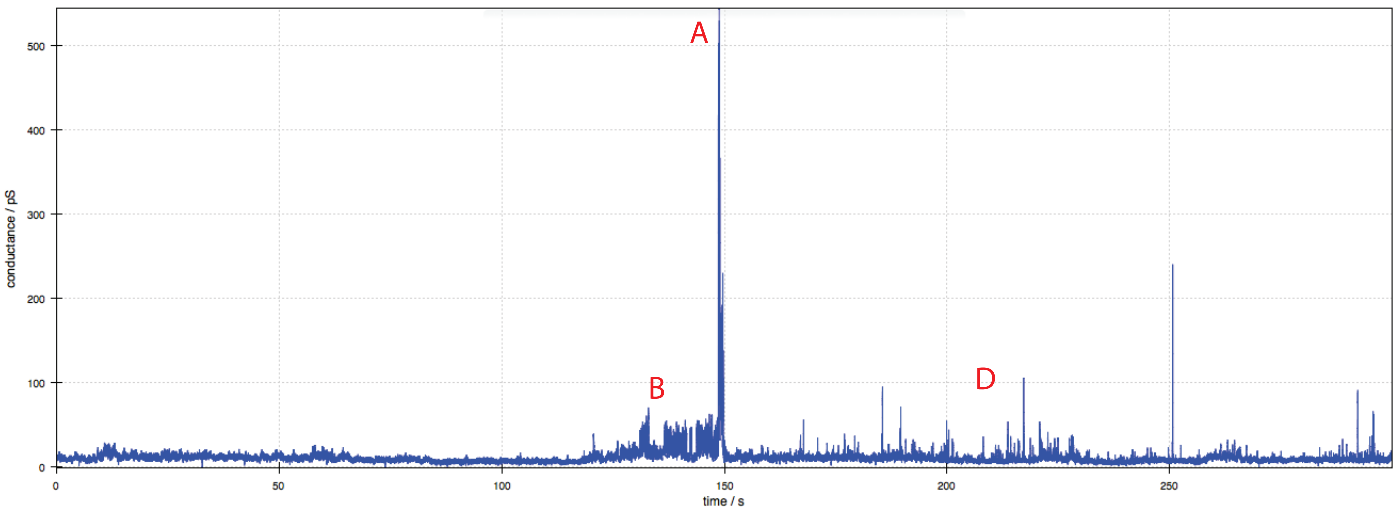

A

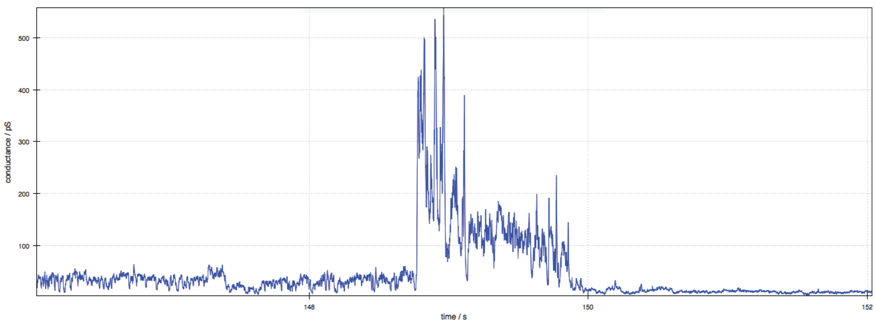

B

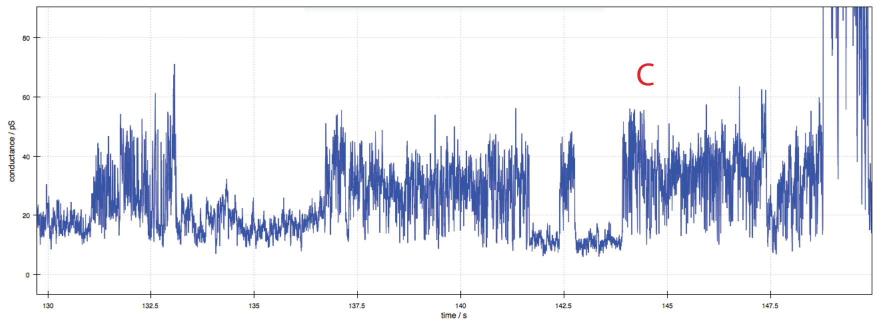

C

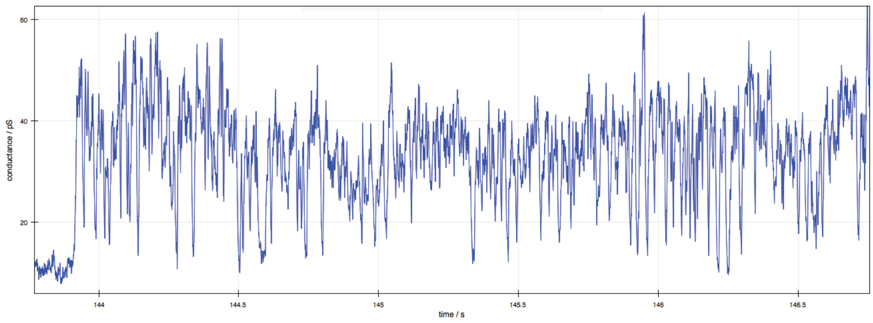

D

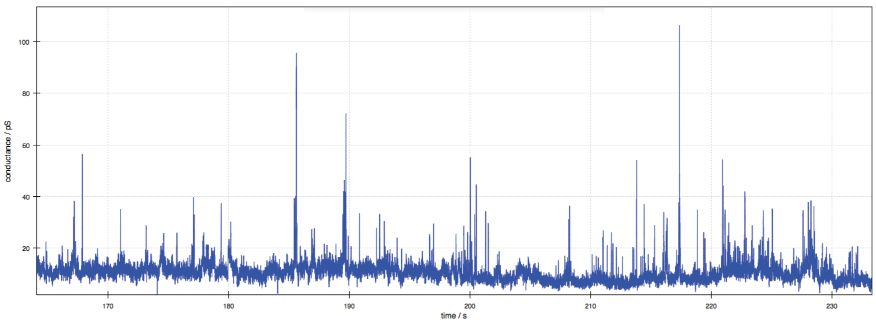

[JC-98] 8-12-8-G 1M CsCl pH 7 homoAdd

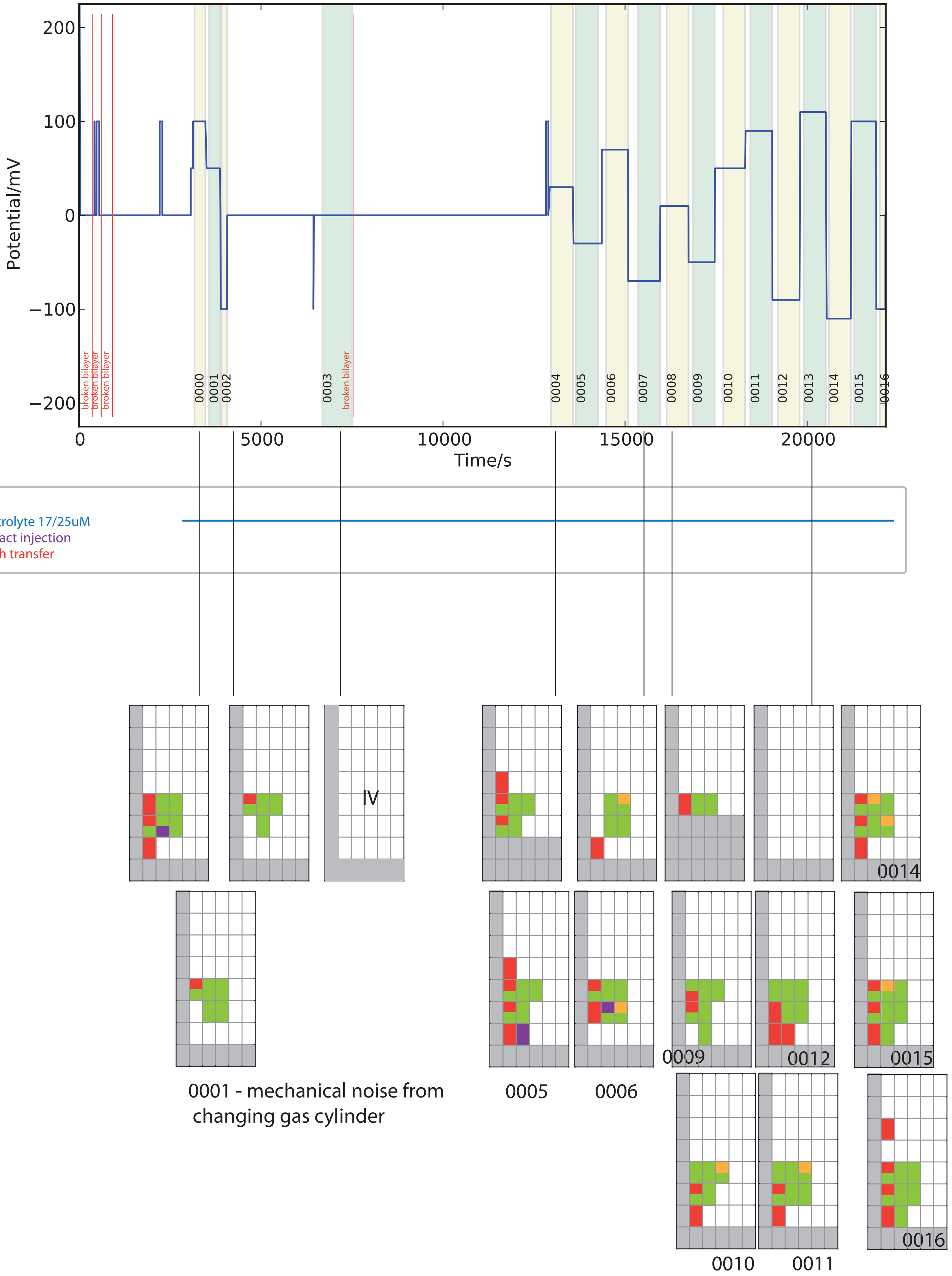

98-0000

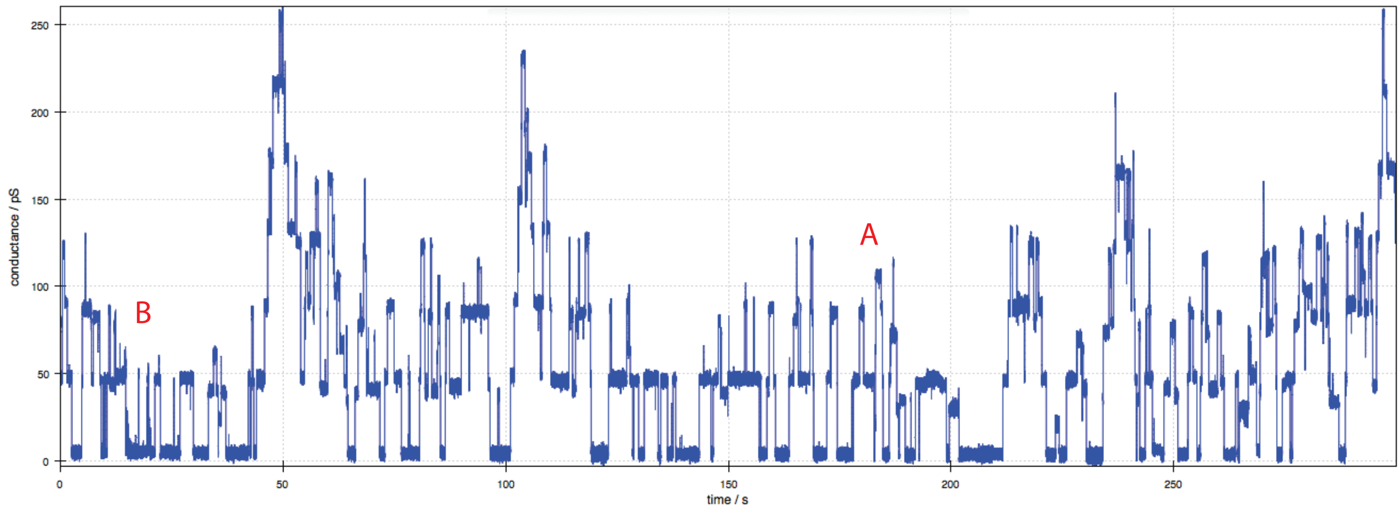

A

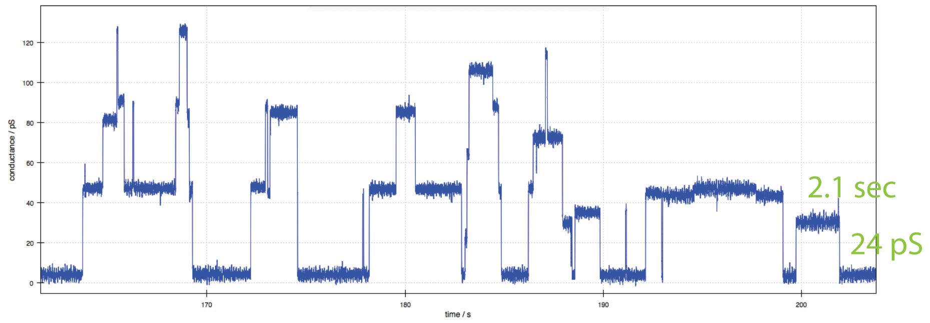

B

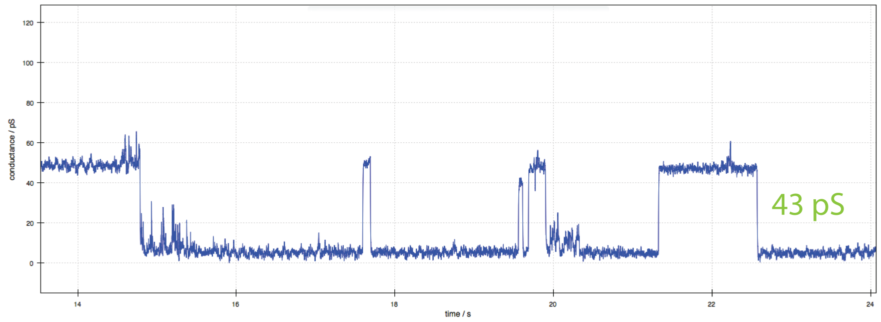

98-0001

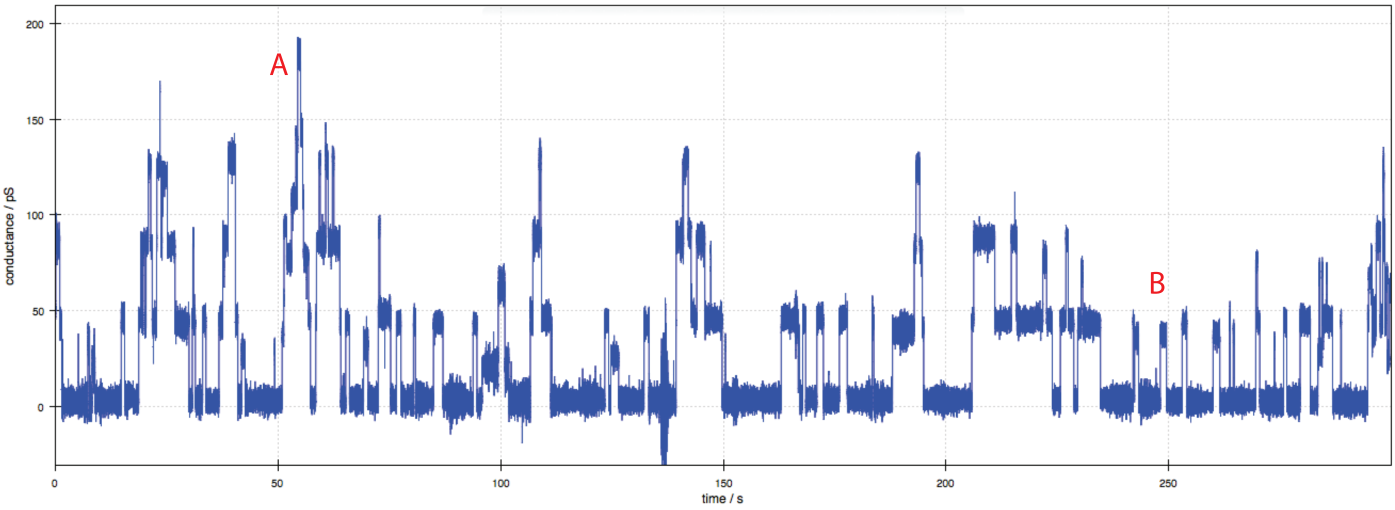

A
B
conductance / pS
time / s
200
150
100
50
0
0
50
100
150
200
250

A

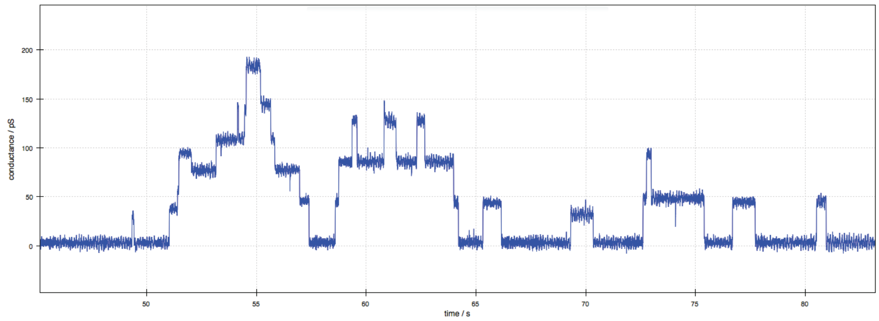

conductance / pS
time / s
200
150
100
50
0
50
55
60
65
70
75
80

B

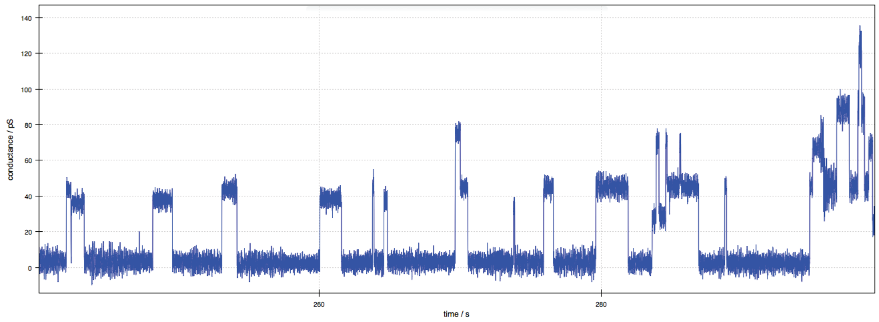

conductance / pS
time / s
140
120
100
80
60
40
20
0
260
280

98-0002

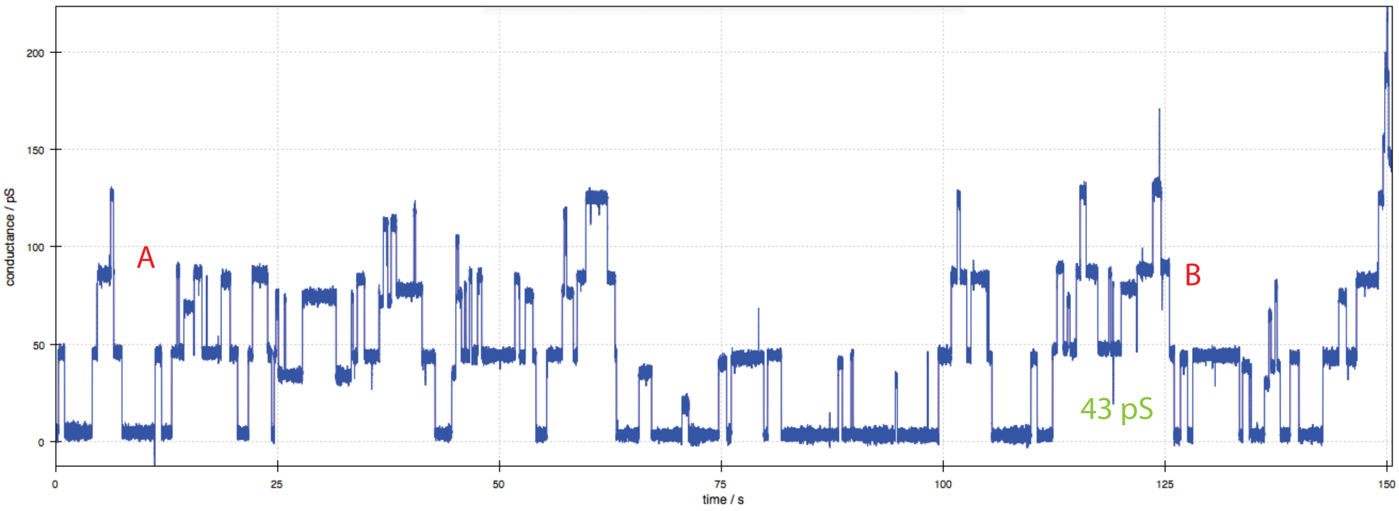

A
B
43 pS
conductance / pS
time / s

A

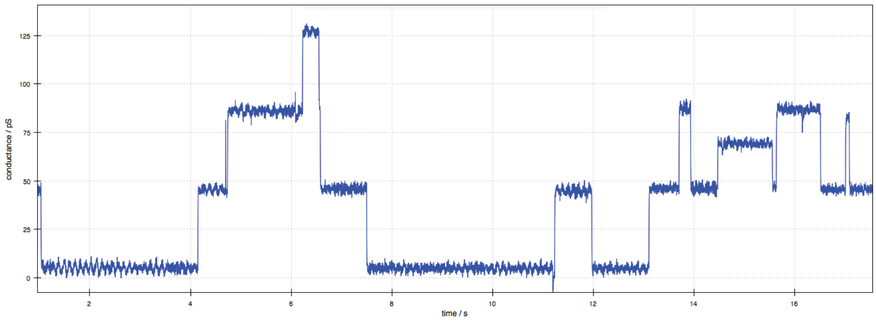

conductance / pS
time / s

B

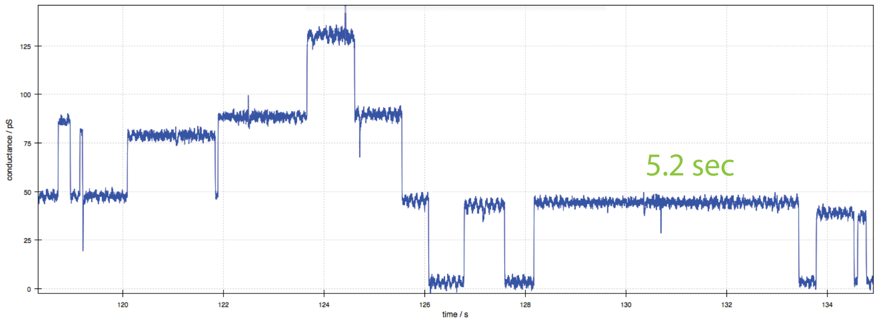

5.2 sec
conductance / pS
time / s

98-0004

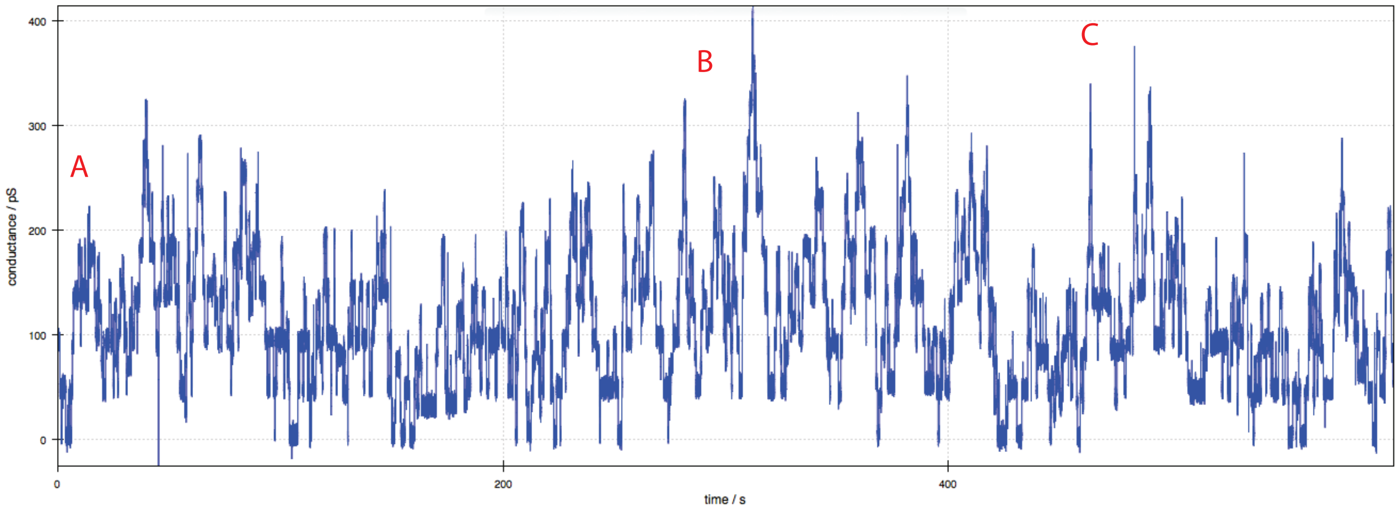
A
B
C
conductance / pS
400
300
200
100
0
0
200
400
time / s

A

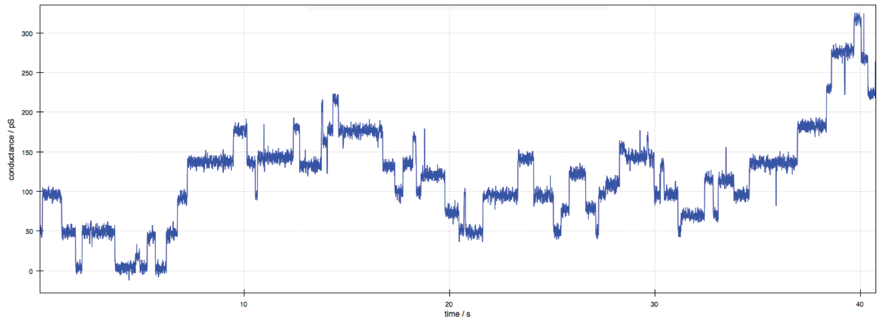
conductance / pS
300
250
200
150
100
50
0
10
20
30
40
time / s

B

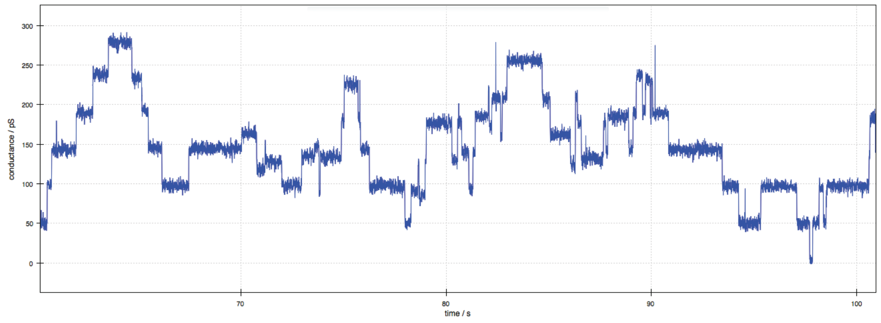
conductance / pS
300
250
200
150
100
50
0
70
80
90
100
time / s

C

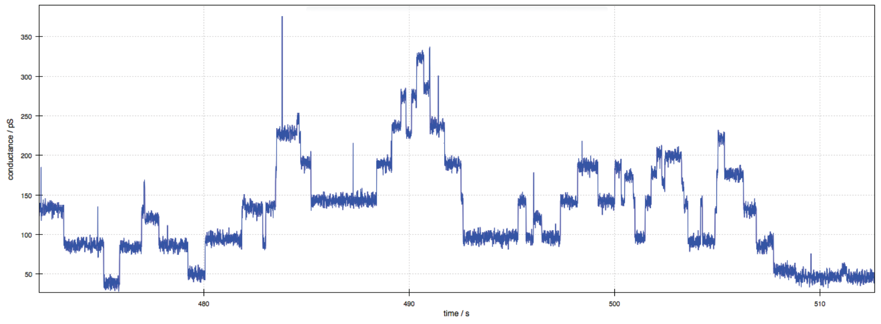
conductance / pS
350
300
250
200
150
100
50
480
490
500
510
time / s

98-0005

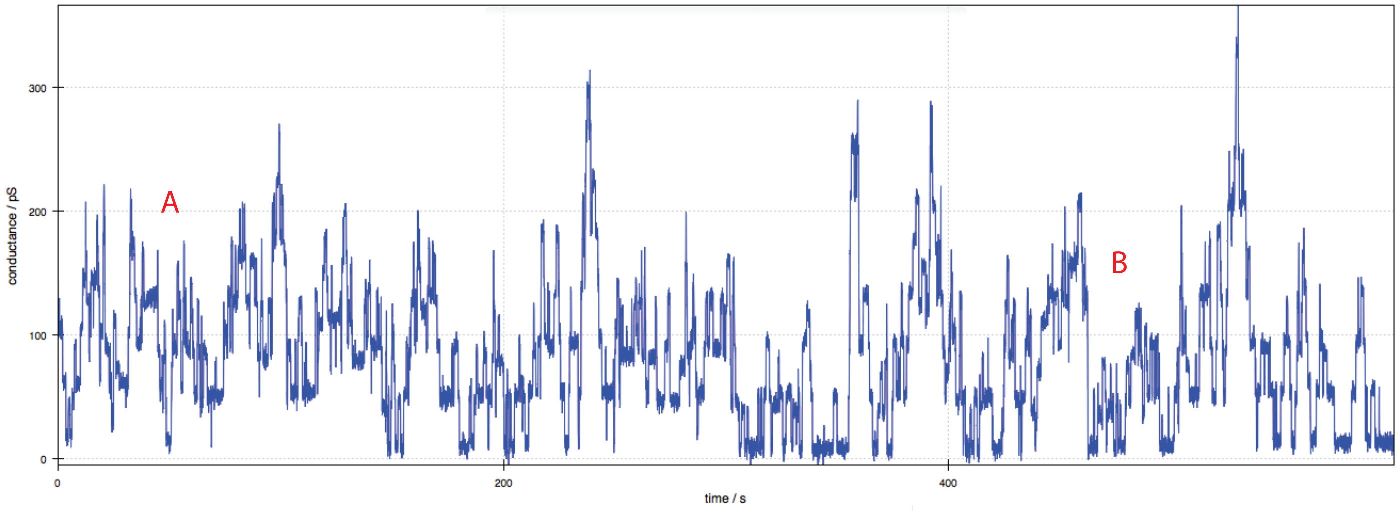
A
B
conductance / pS
300
200
100
0
0
200
400
time / s

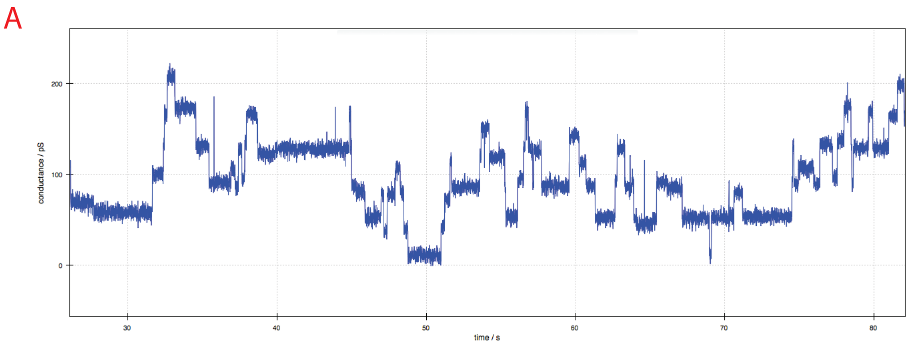
A
conductance / pS
200
100
0
30
40
50
60
70
80
time / s

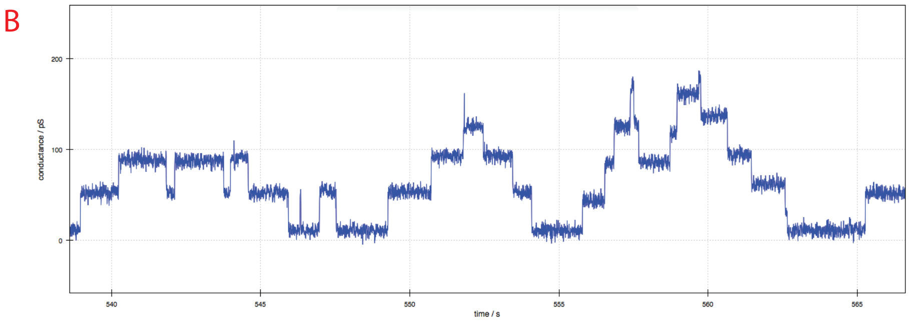
B
conductance / pS
200
100
0
540
545
550
555
560
565
time / s

98-0006

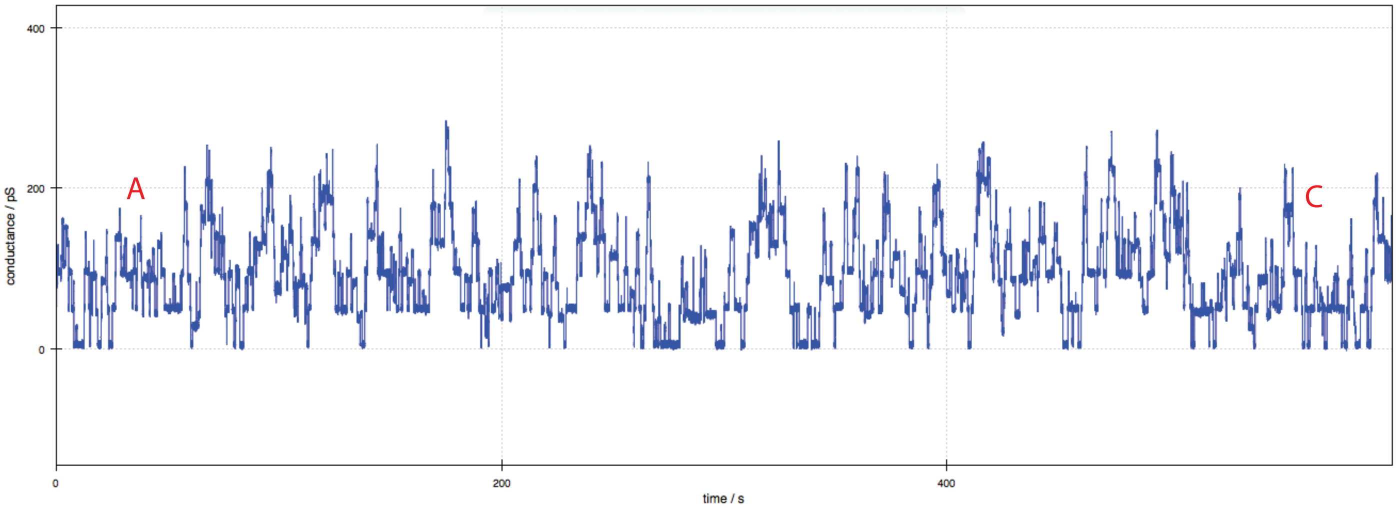

400
200
0
conductance / pS
A
C
0
200
400
time / s

A

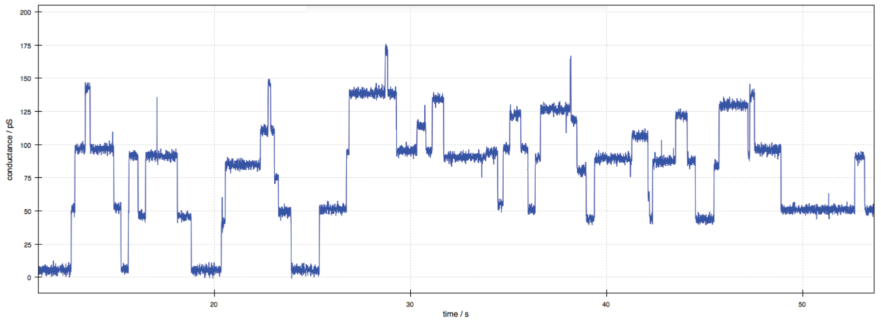

conductance / pS
time / s

B

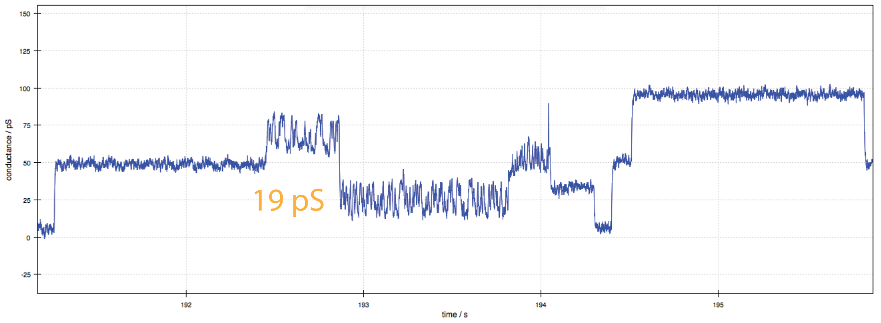

conductance / pS
19 pS
time / s

C

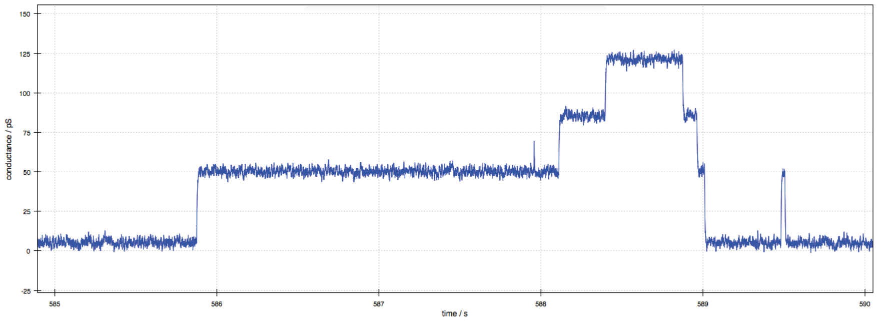

conductance / pS
time / s

98-0007

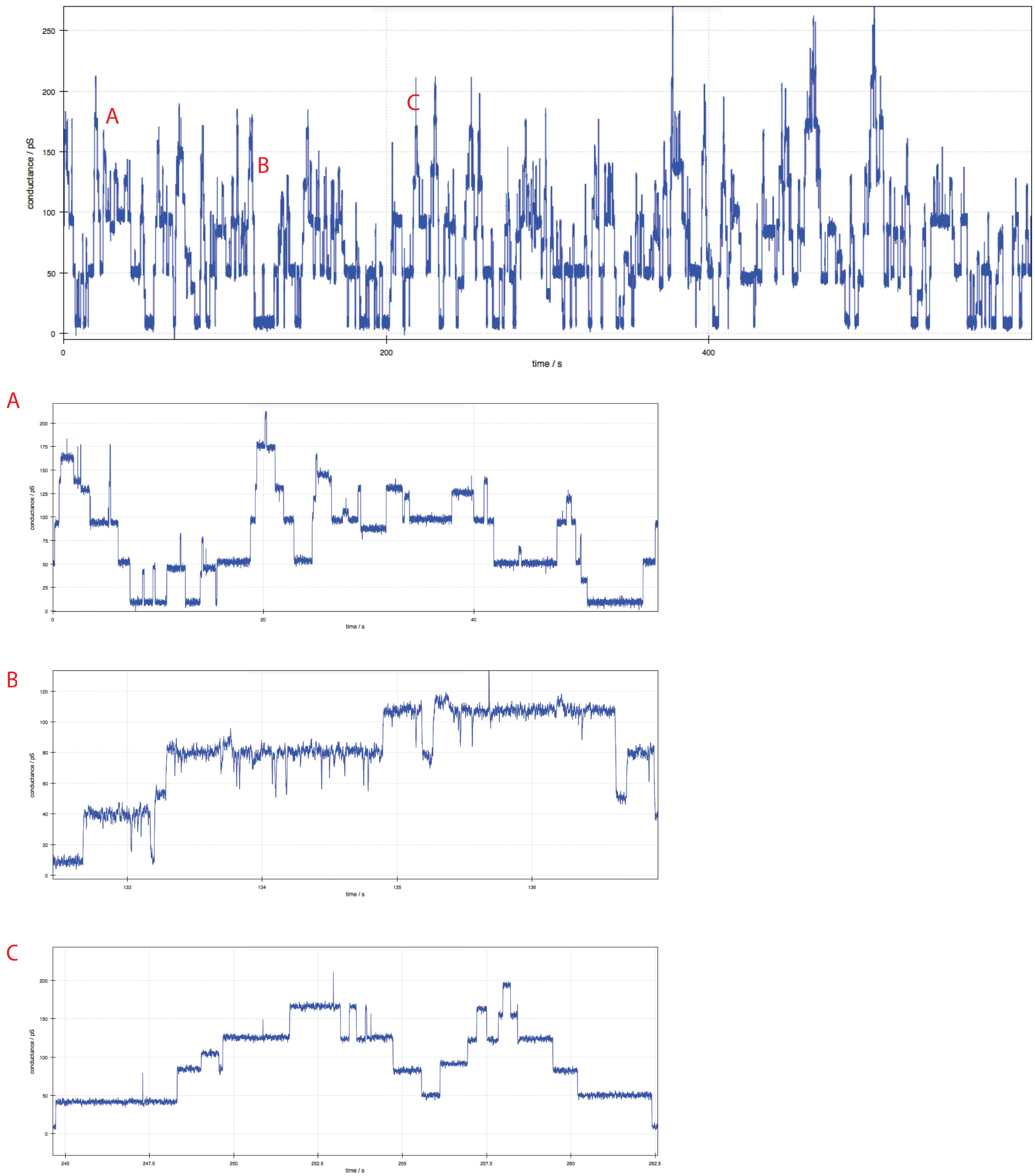

98-0008

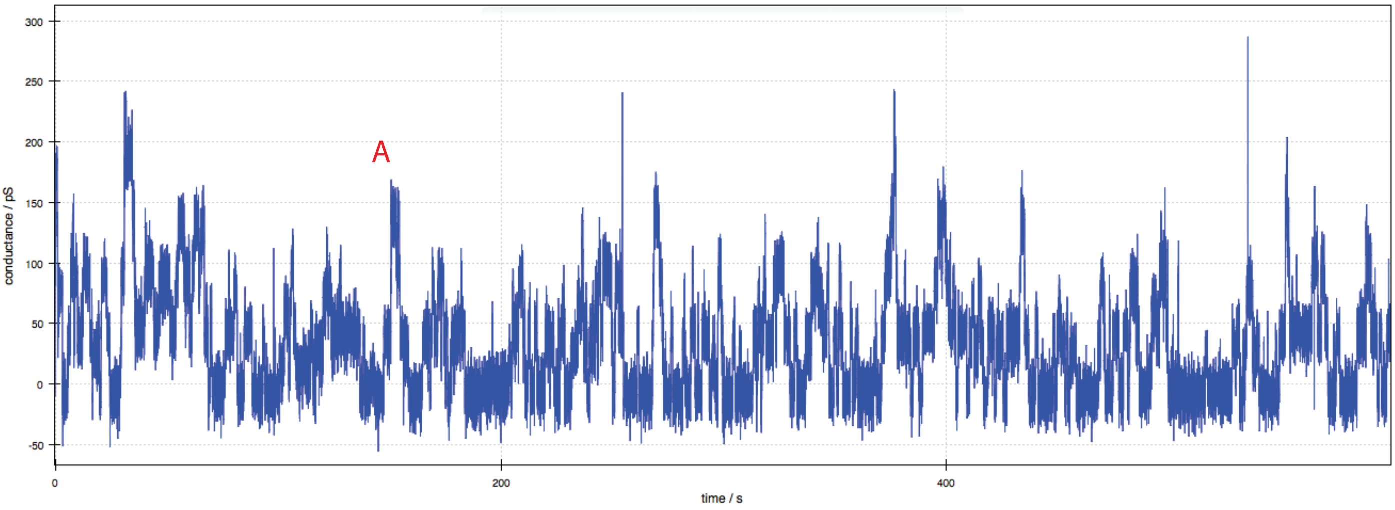

300
250
200
150
100
50
0
-50
conductance / pS
A
0
200
400
time / s

A

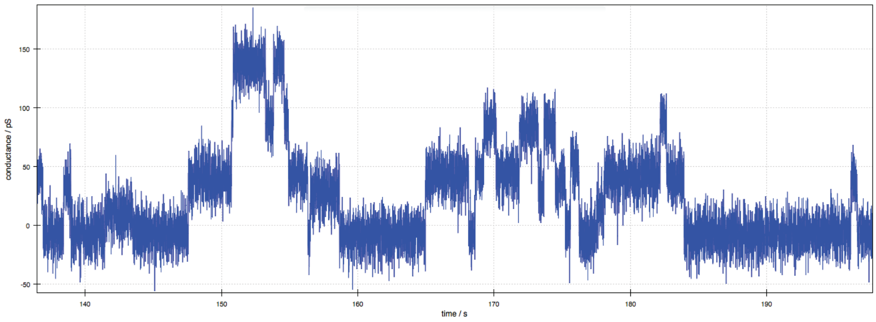

150
100
50
0
-50
conductance / pS
140
150
160
170
180
190
time / s

98-0009

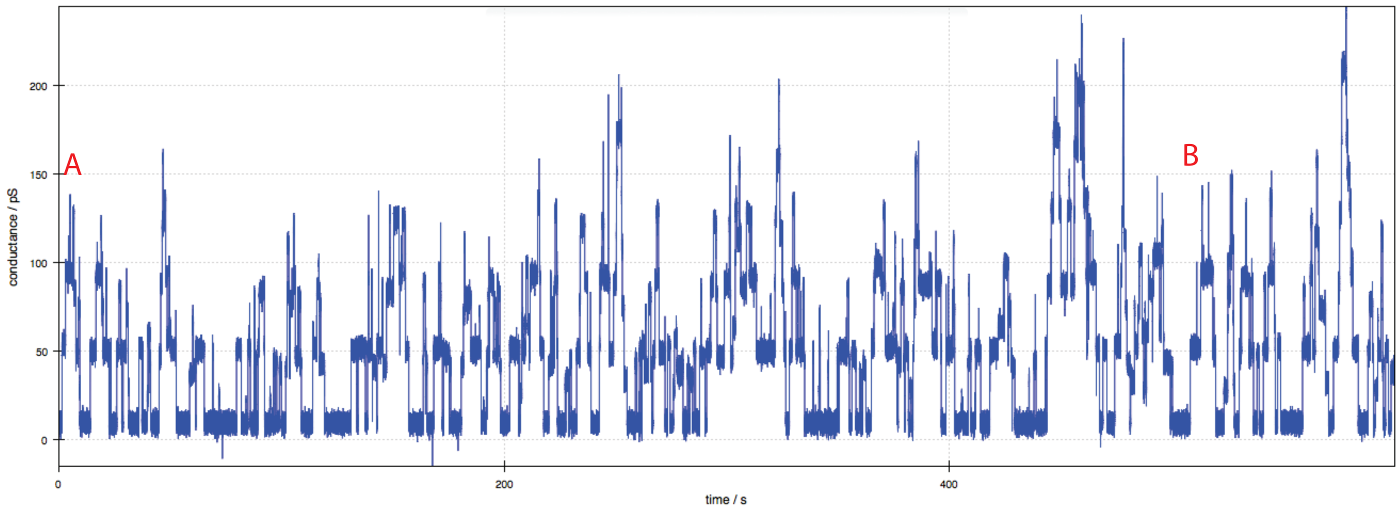
A
B
conductance / pS
200
150
100
50
0
0
200
400
time / s

A

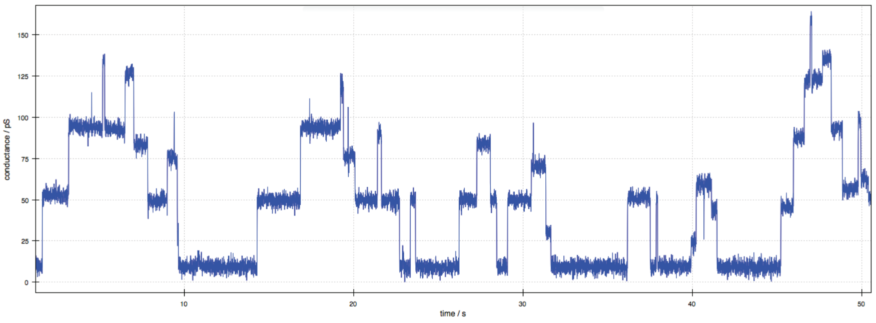
conductance / pS
150
125
100
75
50
25
0
10
20
30
40
50
time / s

B

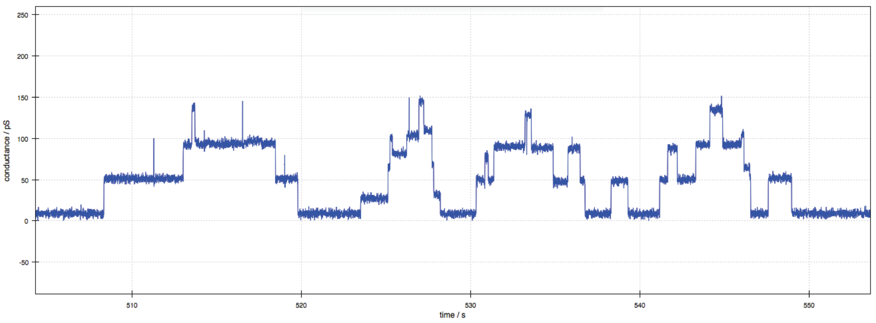
conductance / pS
250
200
150
100
50
0
-50
510
520
530
540
550
time / s

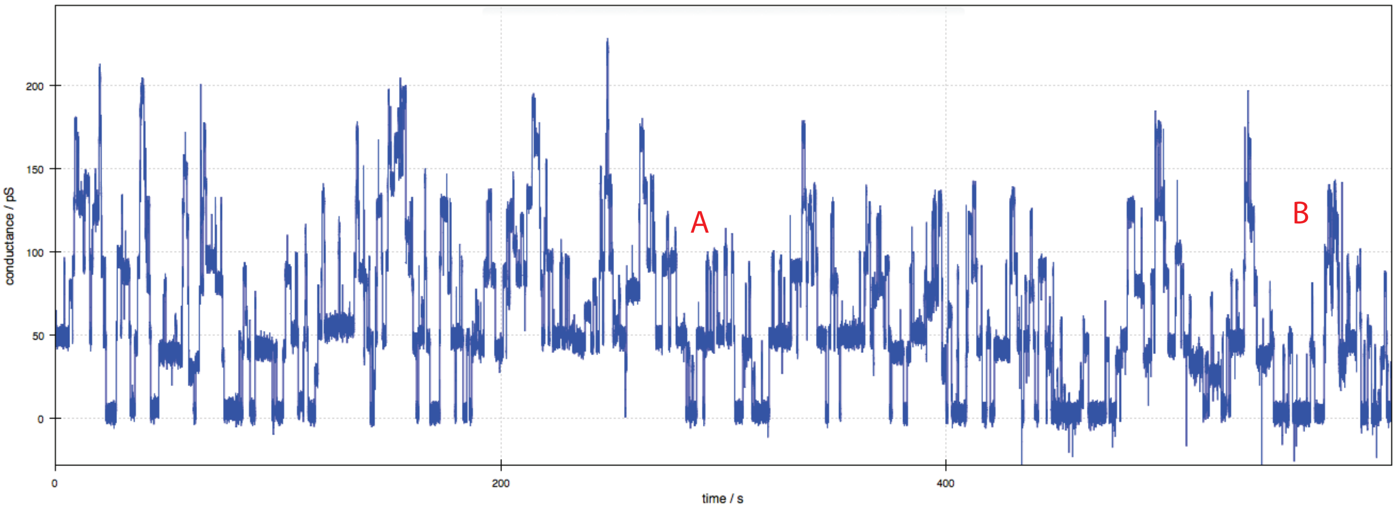
conductance / pS
0
50
100
150
200
0
200
400
time / s
A
B

A

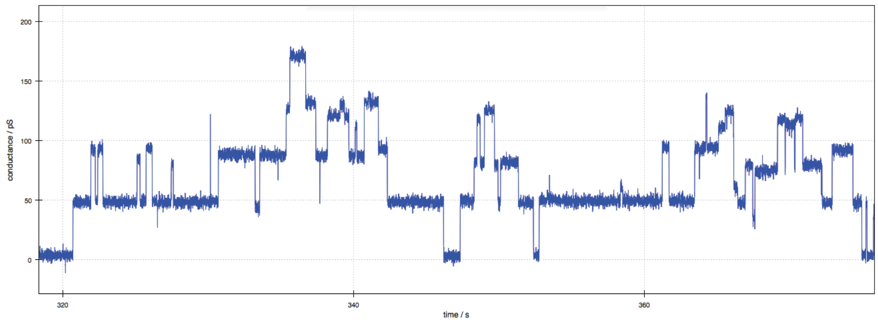
conductance / pS
0
50
100
150
200
320
340
360
time / s

B

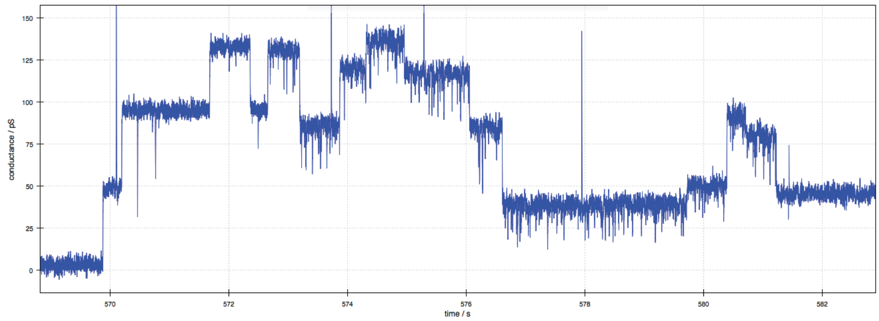
conductance / pS
0
25
50
75
100
125
150
570
572
574
576
578
580
582
time / s

98-0011

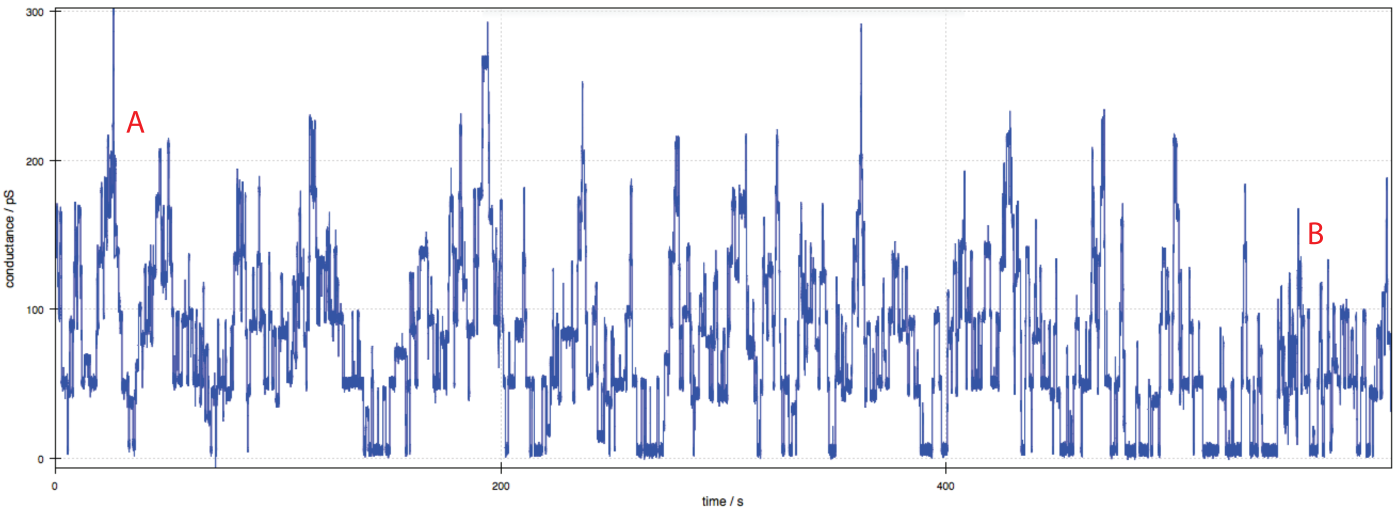

300
200
100
0
conductance / pS
A
B
0
200
400
time / s

A

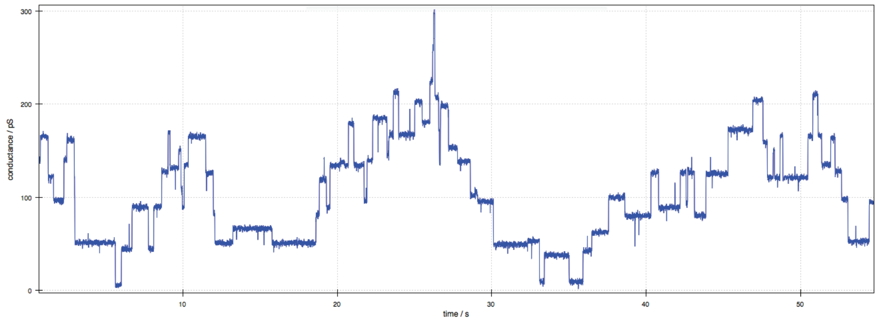

300
200
100
0
conductance / pS
10
20
30
40
50
time / s

B

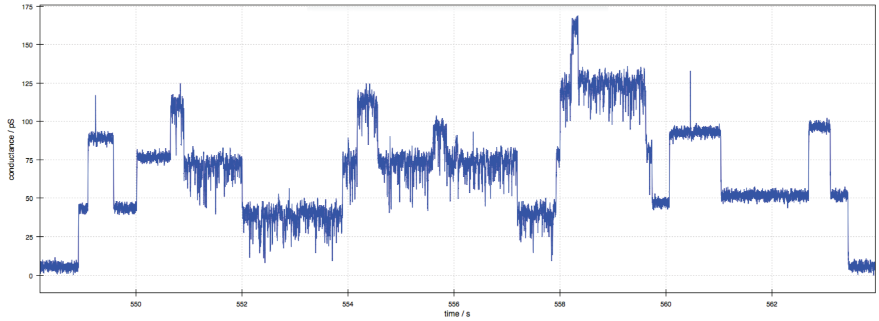

175
150
125
100
75
50
25
0
conductance / pS
550
552
554
556
558
560
562
time / s

98-0012

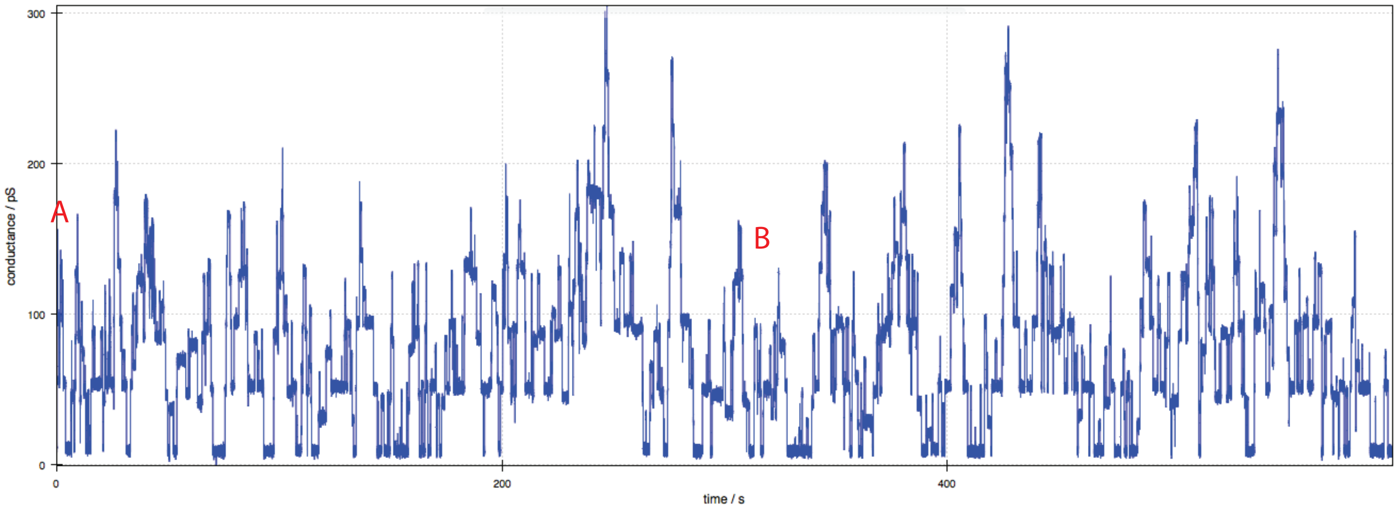

A

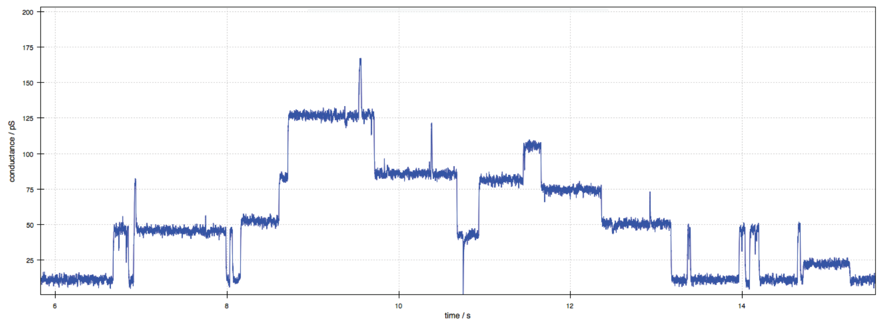

B

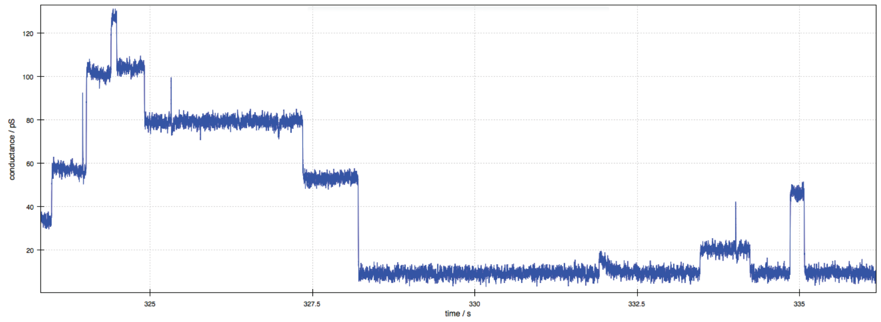

C

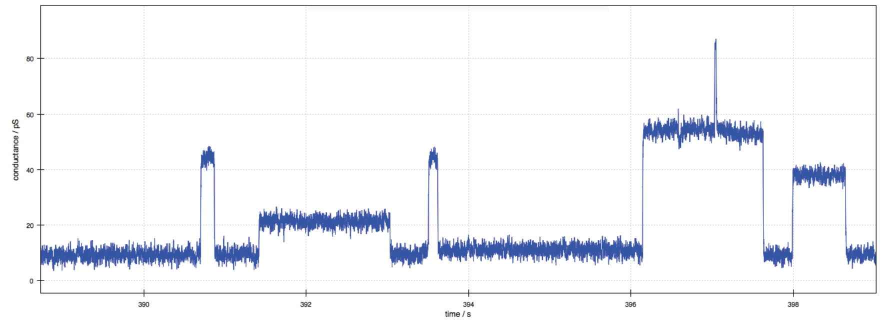

multiple populations of (small) openings

98-0013

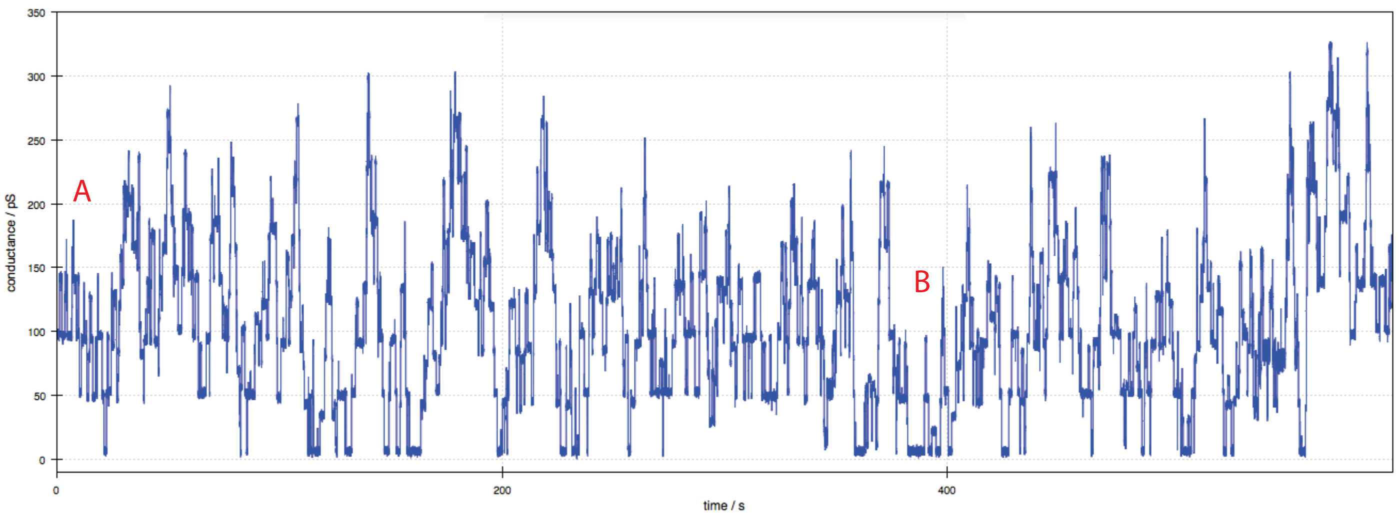
A
B
conductance / pS
time / s
0
50
100
150
200
250
300
350
0
200
400

A

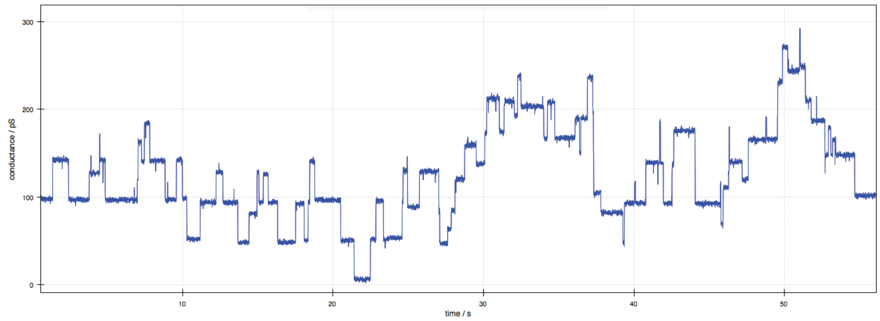
conductance / pS
time / s
0
100
200
300
10
20
30
40
50

B

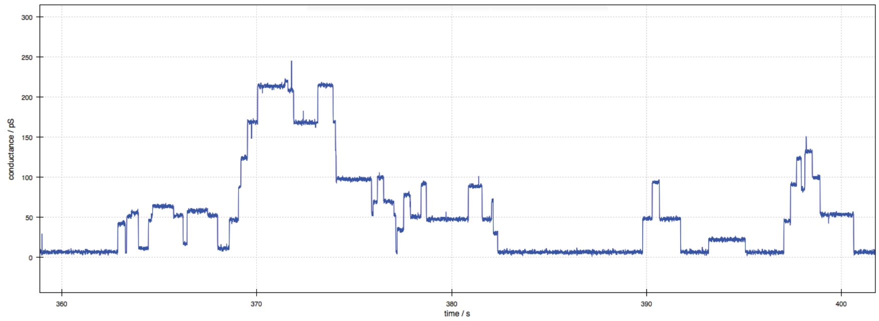
conductance / pS
time / s
0
50
100
150
200
250
300
360
370
380
390
400

C

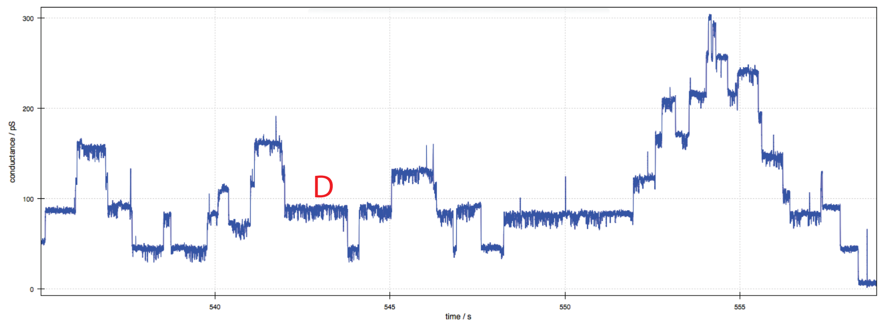
D
conductance / pS
time / s
0
100
200
300
540
545
550
555

D

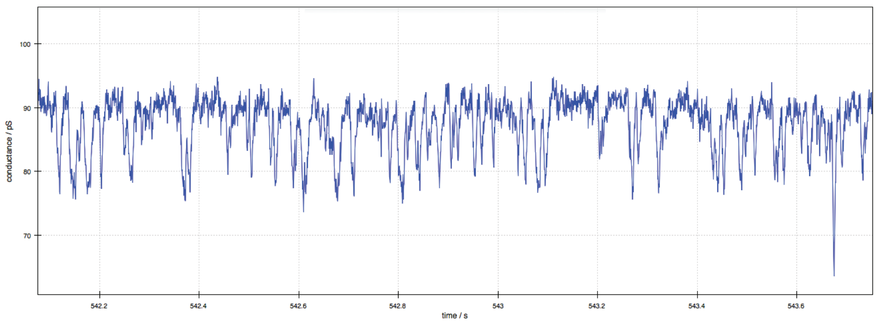
conductance / pS
time / s
70
80
90
100
542.2
542.4
542.6
542.8
543
543.2
543.4
543.6

98-0014

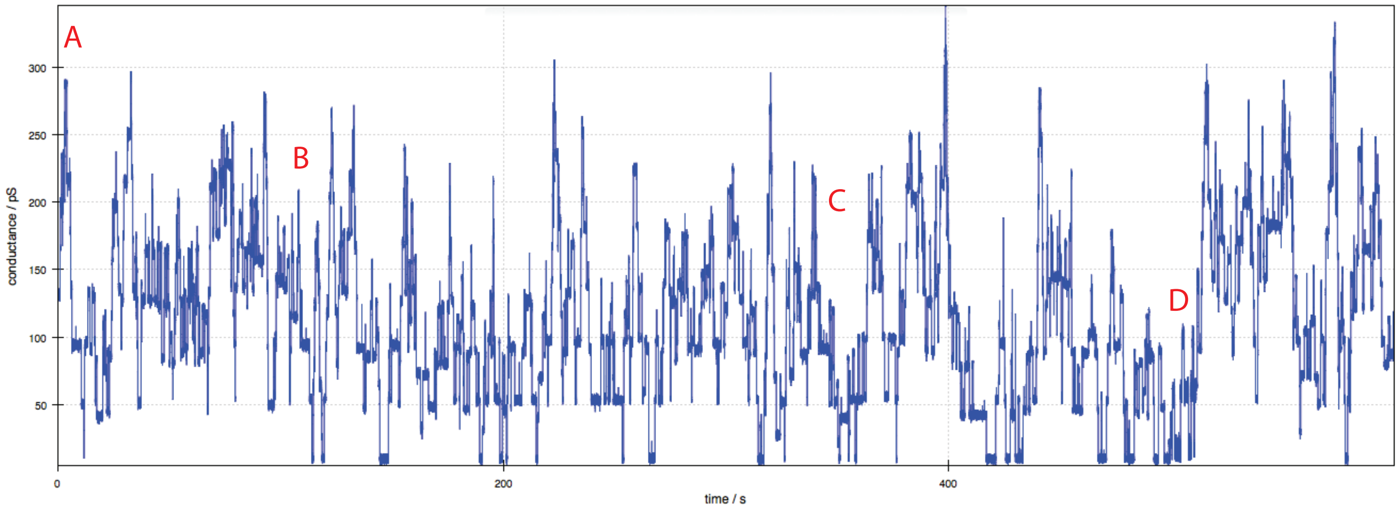

A
B
C
D
300
250
200
150
100
50
conductance / pS
0
200
400
time / s

A

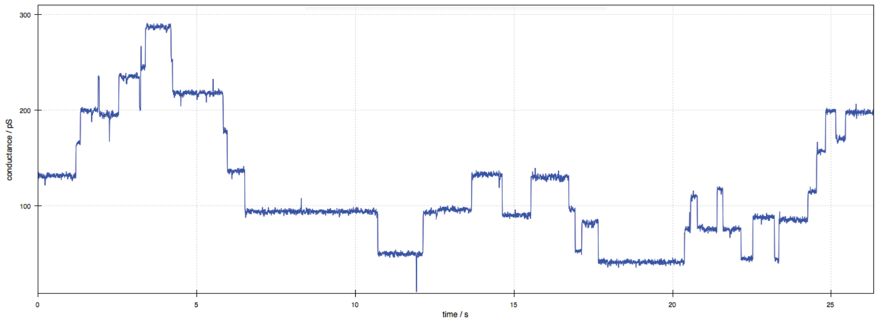

300
200
100
conductance / pS
0
5
10
15
20
25
time / s

B

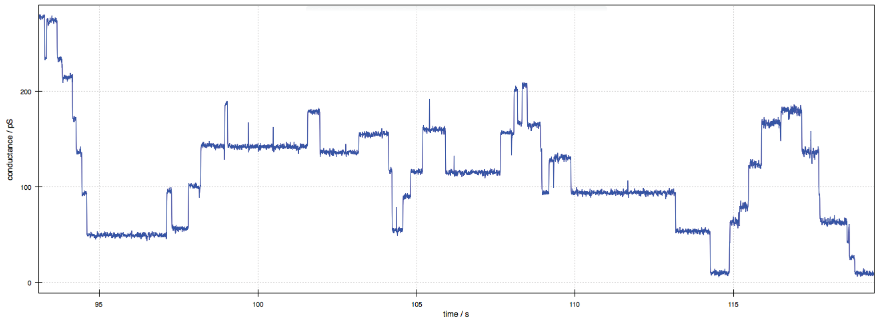

200
100
0
conductance / pS
95
100
105
110
115
time / s

C

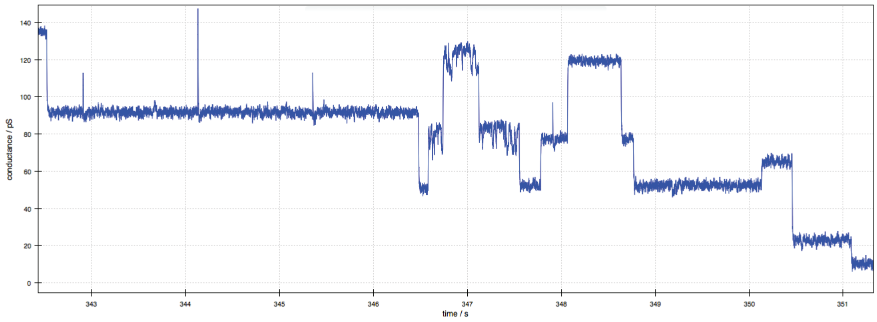

140
120
100
80
60
40
20
0
conductance / pS
343
344
345
346
347
348
349
350
351
time / s

D

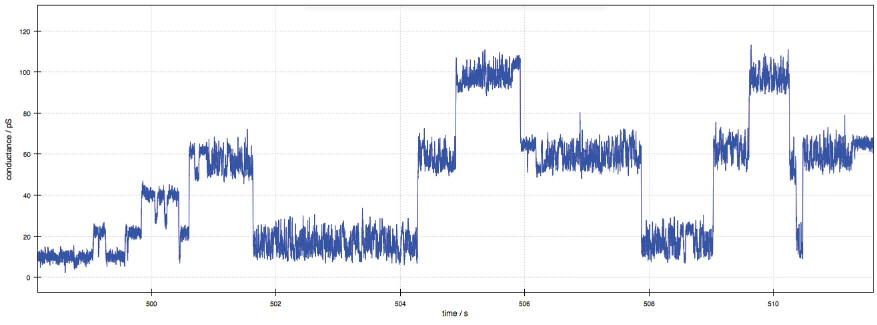

120
100
80
60
40
20
0
conductance / pS
500
502
504
506
508
510
time / s

98-0015

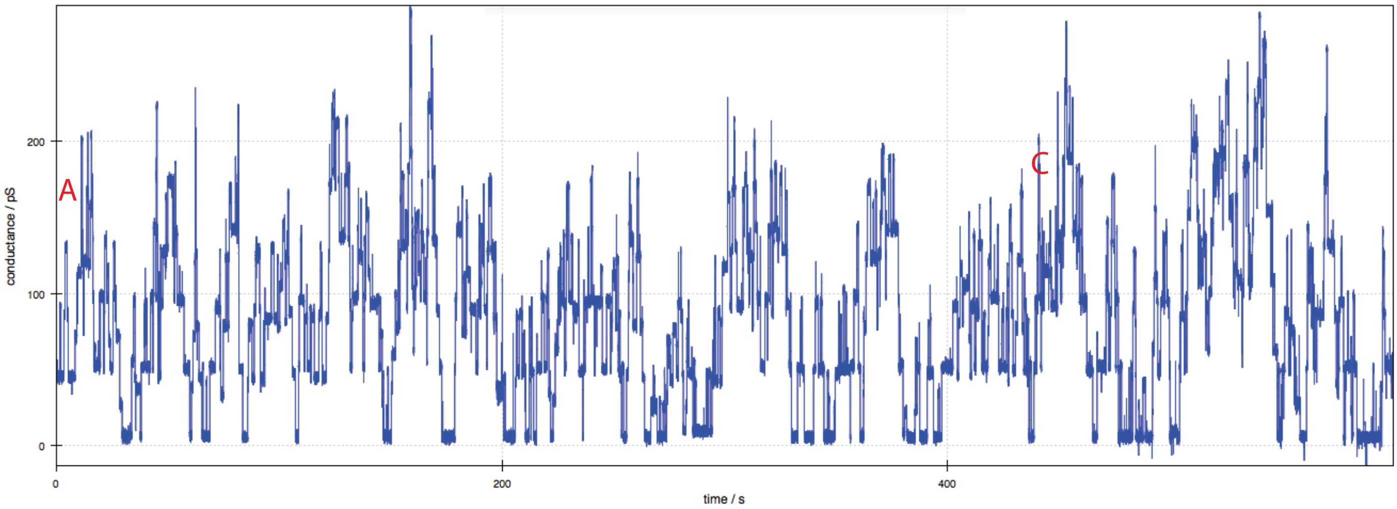

A
C
200
100
0
conductance / pS
0
200
400
time / s

A

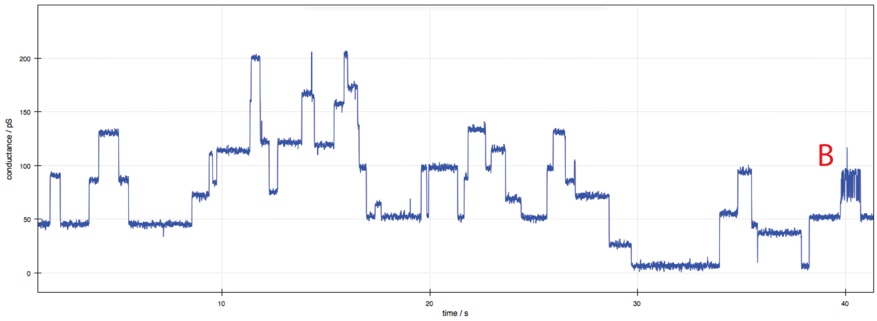

B
200
150
100
50
0
conductance / pS
10
20
30
40
time / s

B

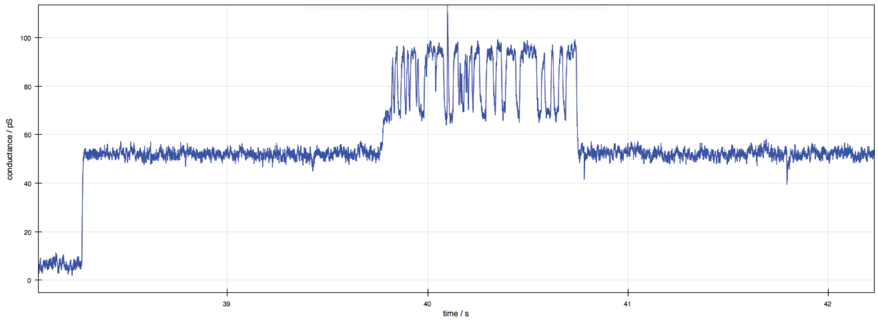

100
80
60
40
20
0
conductance / pS
39
40
41
42
time / s

C

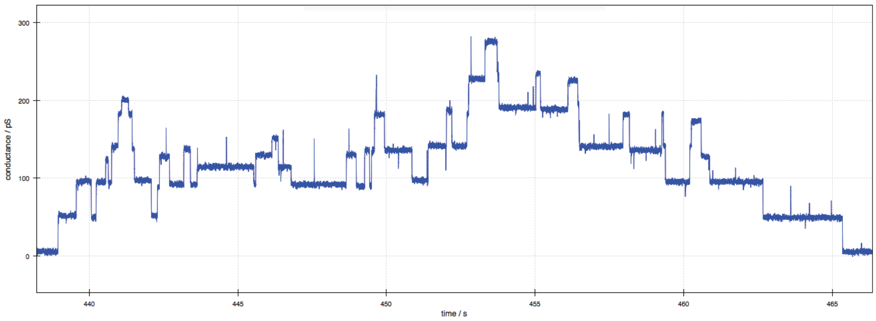

300
200
100
0
conductance / pS
440
445
450
455
460
465
time / s

98-0016

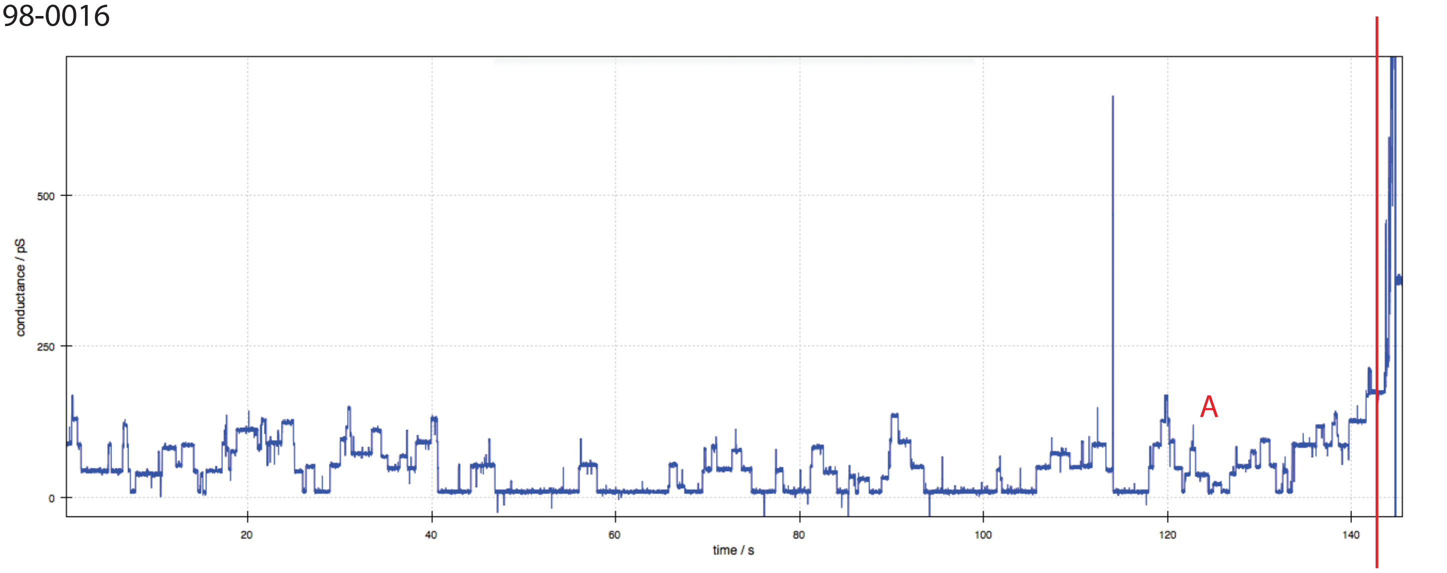

conductance / pS
500
250
0
20
40
60
80
100
120
140
time / s
A

A

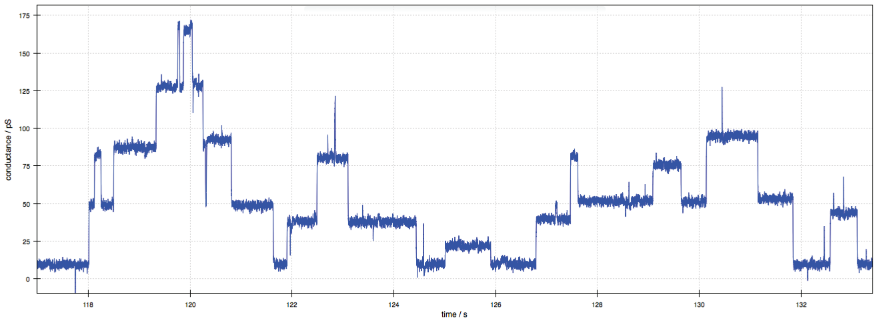

conductance / pS
175
150
125
100
75
50
25
0
118
120
122
124
126
128
130
132
time / s

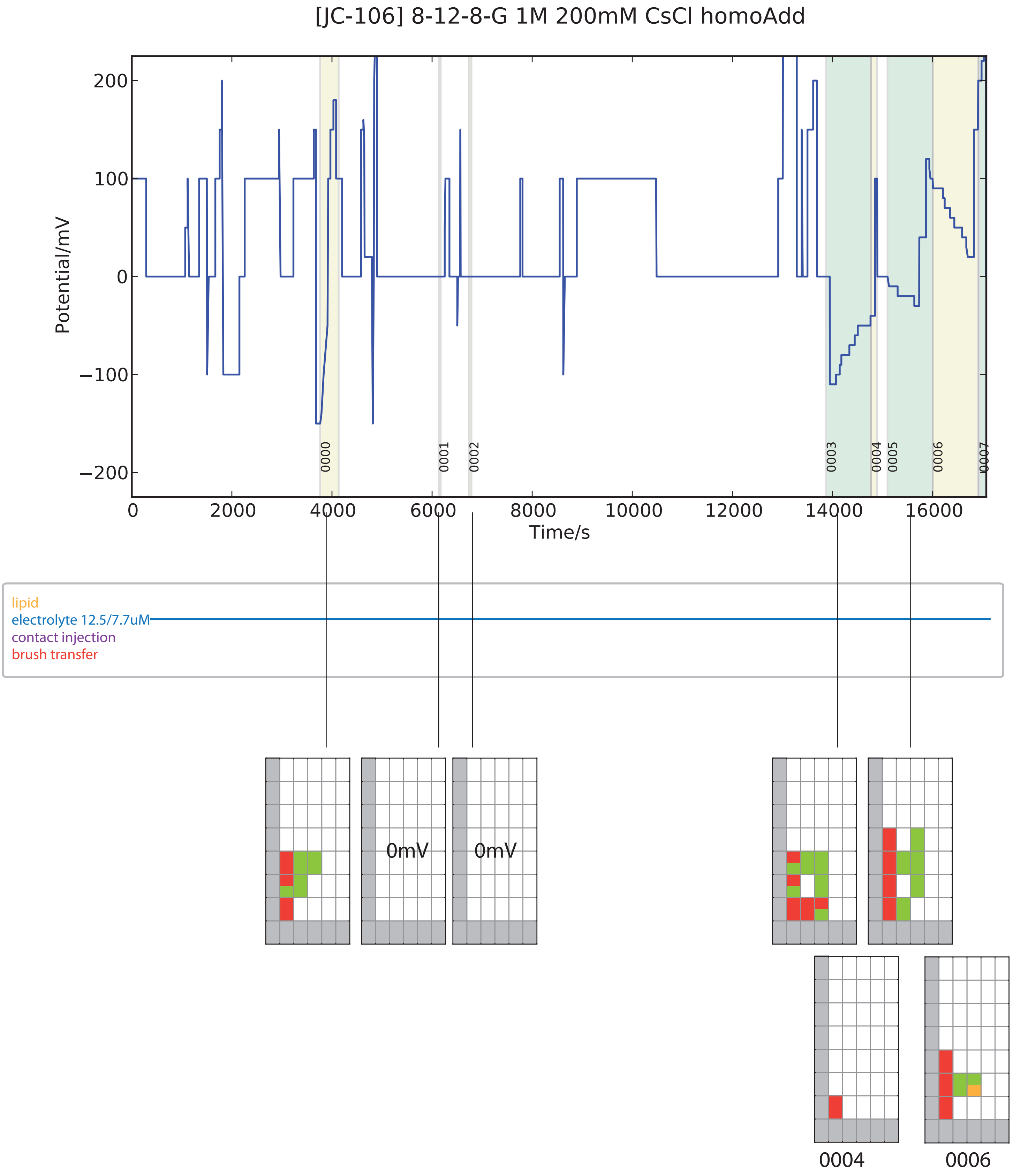
[JC-106] 8-12-8-G 1M 200mM CsCl homoAdd
Potential/mV
Time/s
0000
0001
0002
0003
0004
0005
0006
0007
lipid
electrolyte 12.5/7.7uM
contact injection
brush transfer
0mV
0mV
0004
0006

106-0000

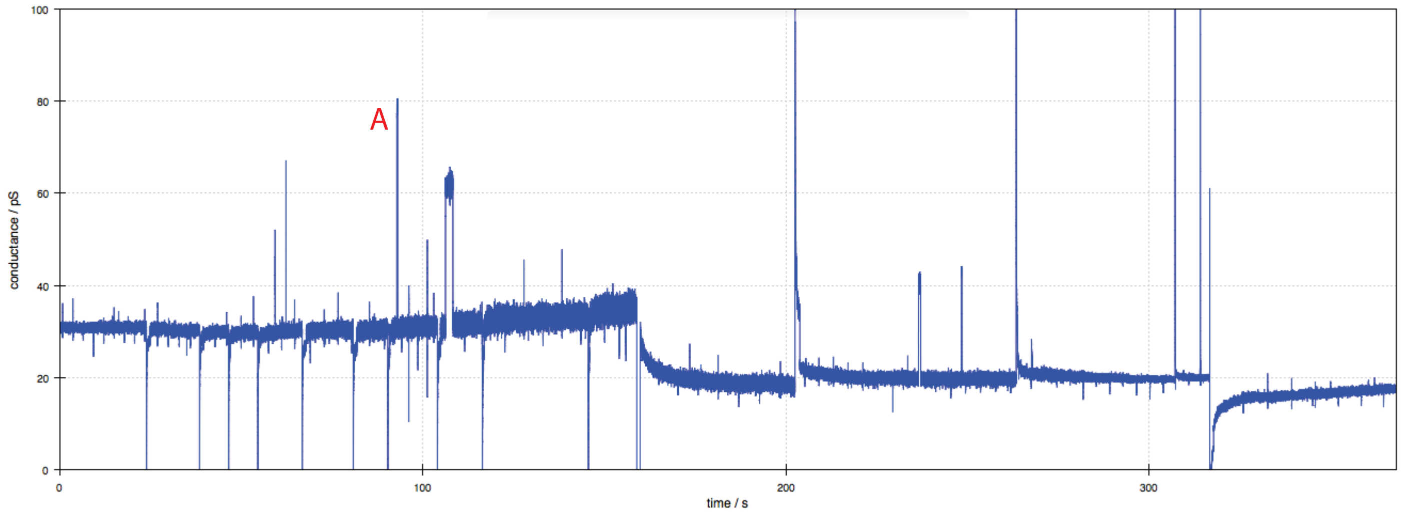
A
conductance / pS
time / s
0
20
40
60
80
100
0
100
200
300

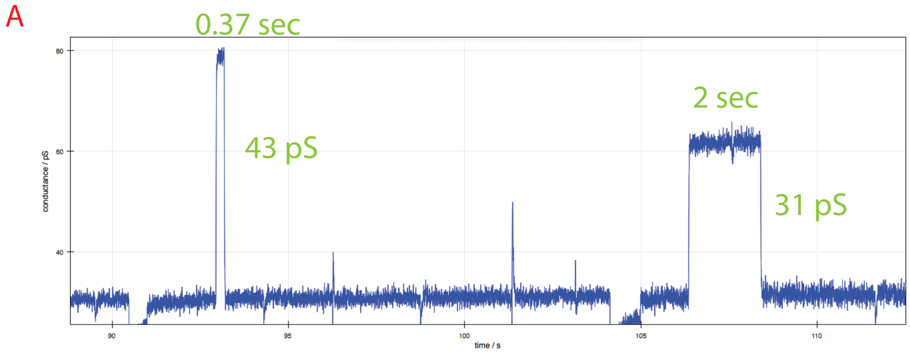
A
0.37 sec
43 pS
2 sec
31 pS
conductance / pS
time / s

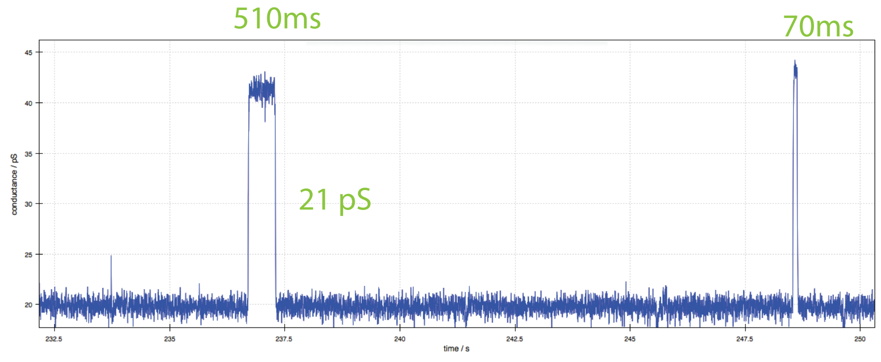
510ms
70ms
21 pS
conductance / pS
time / s

106-0003

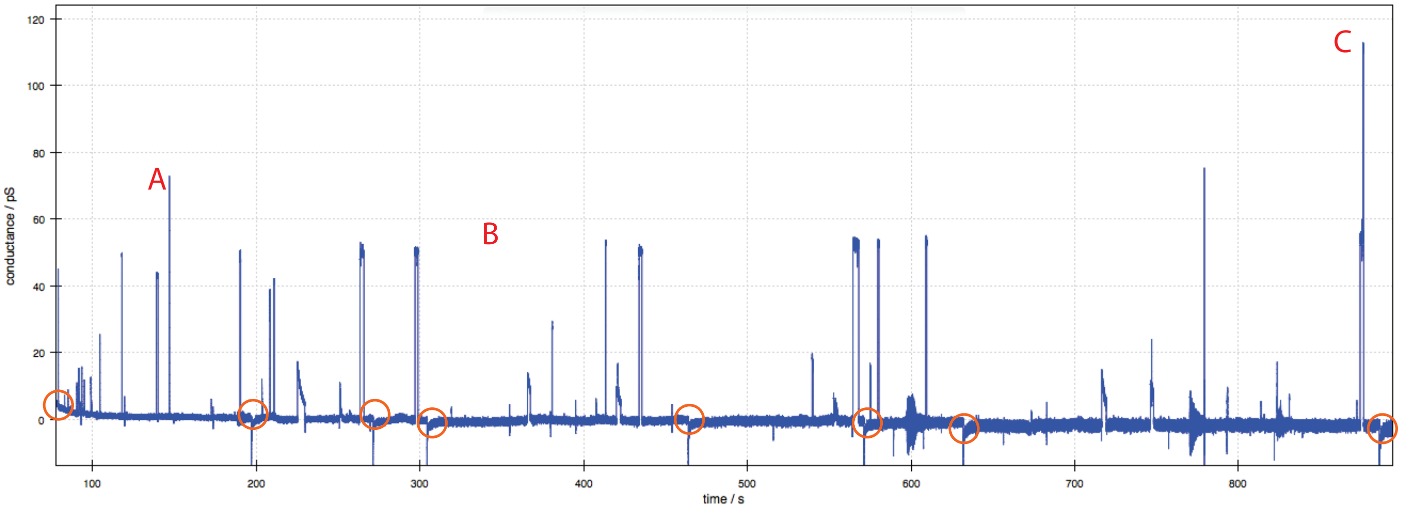

C
A
B
conductance / pS
time / s
120
100
80
60
40
20
0
100
200
300
400
500
600
700
800

A

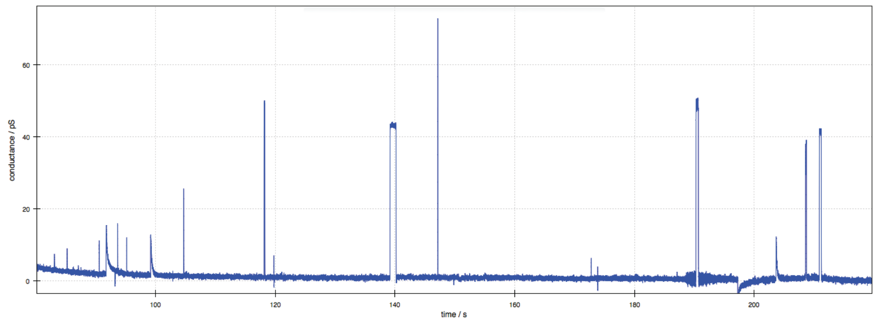

conductance / pS
time / s
60
40
20
0
100
120
140
160
180
200

B

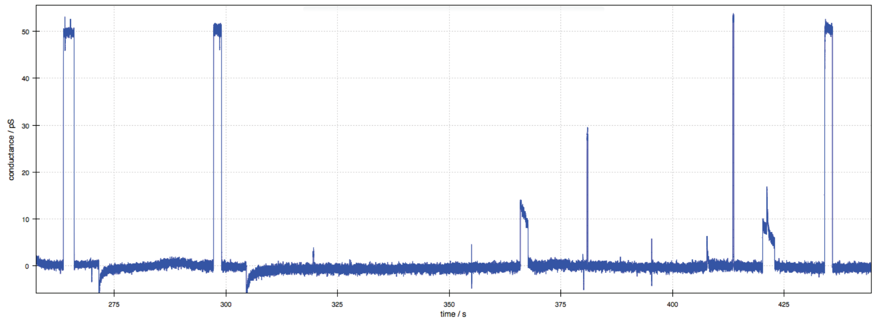

conductance / pS
time / s
50
40
30
20
10
0
275
300
325
350
375
400
425

C

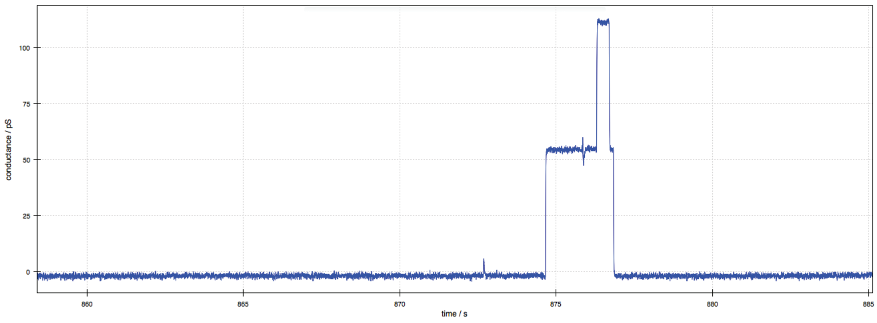

conductance / pS
time / s
100
75
50
25
0
860
865
870
875
880
885

106-0004

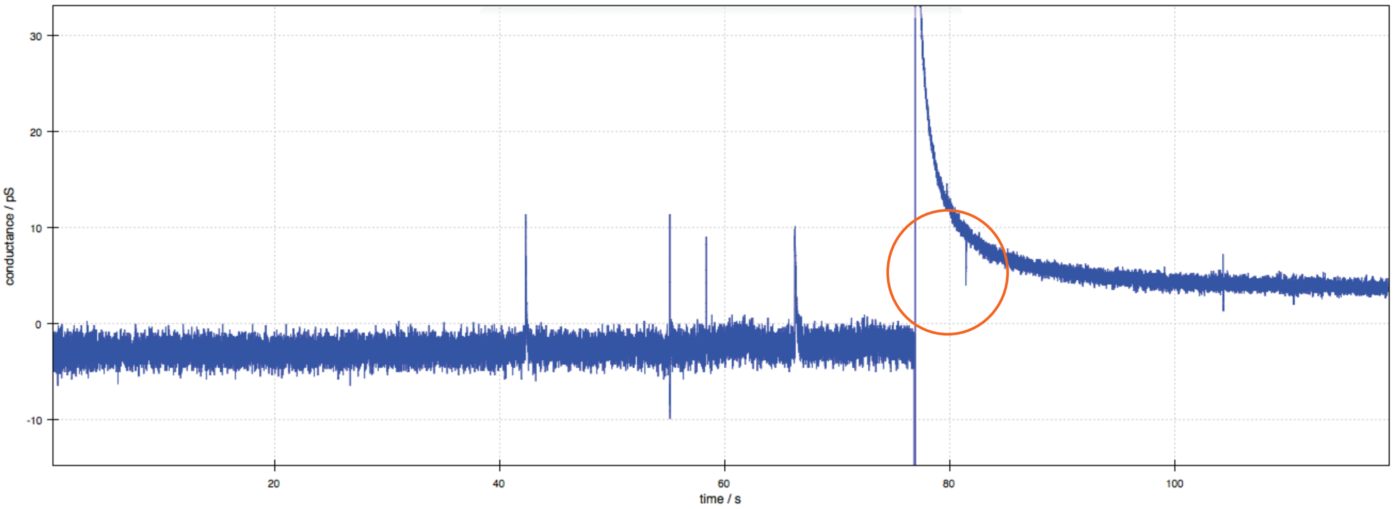

106-0005

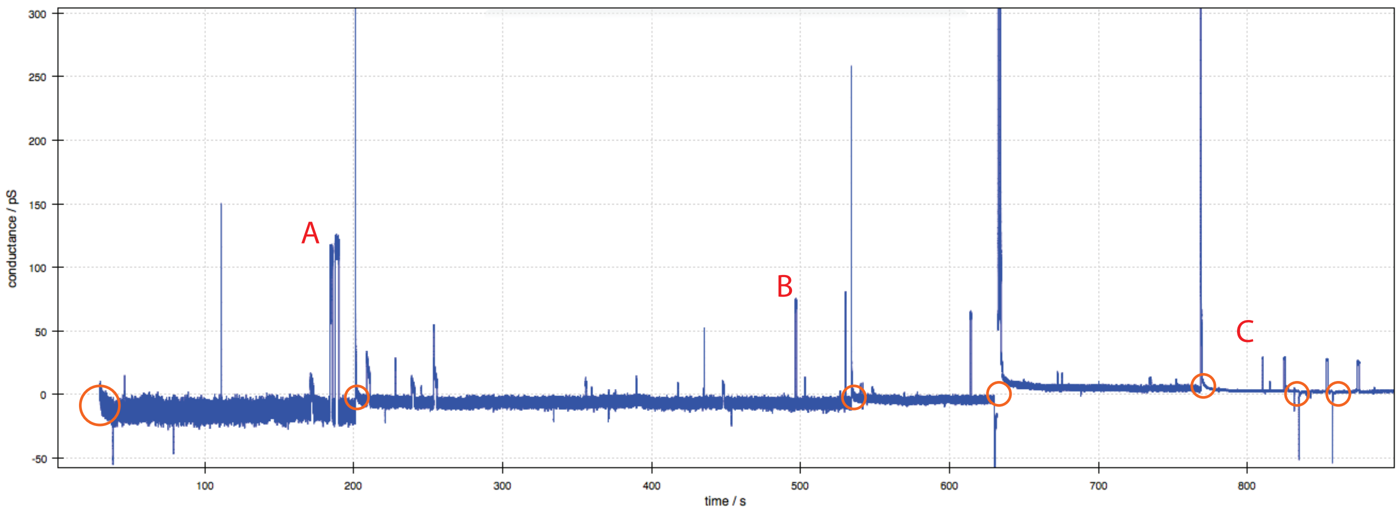

conductance / pS
time / s
A
B
C

A

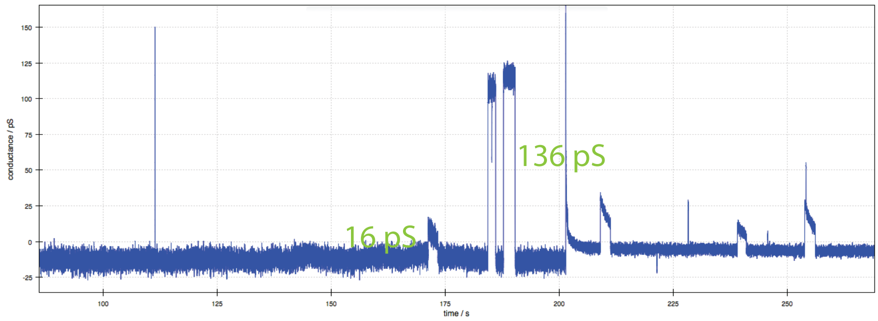

136 pS
16 pS
conductance / pS
time / s

B

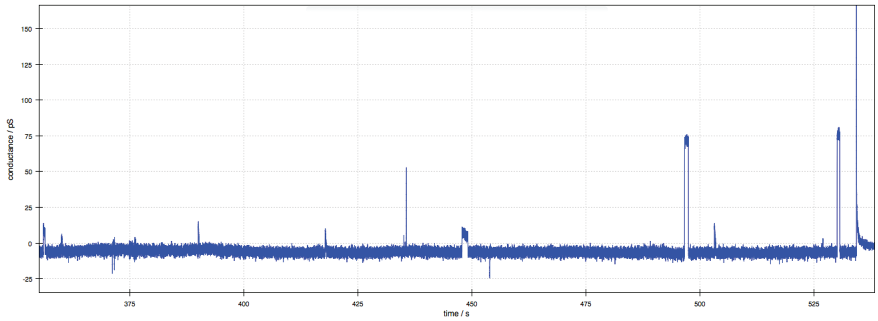

conductance / pS
time / s

C

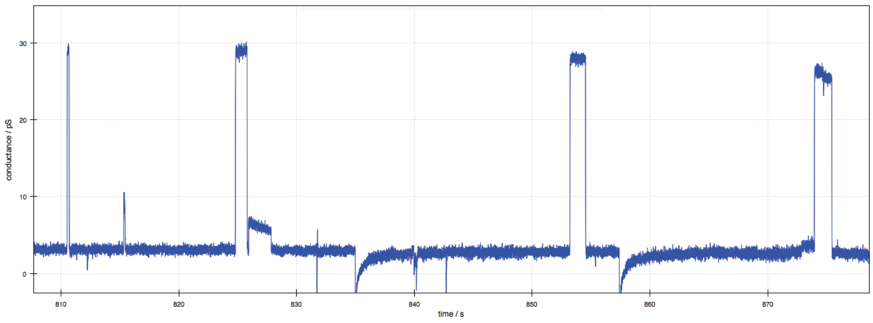

conductance / pS
time / s

106-0006

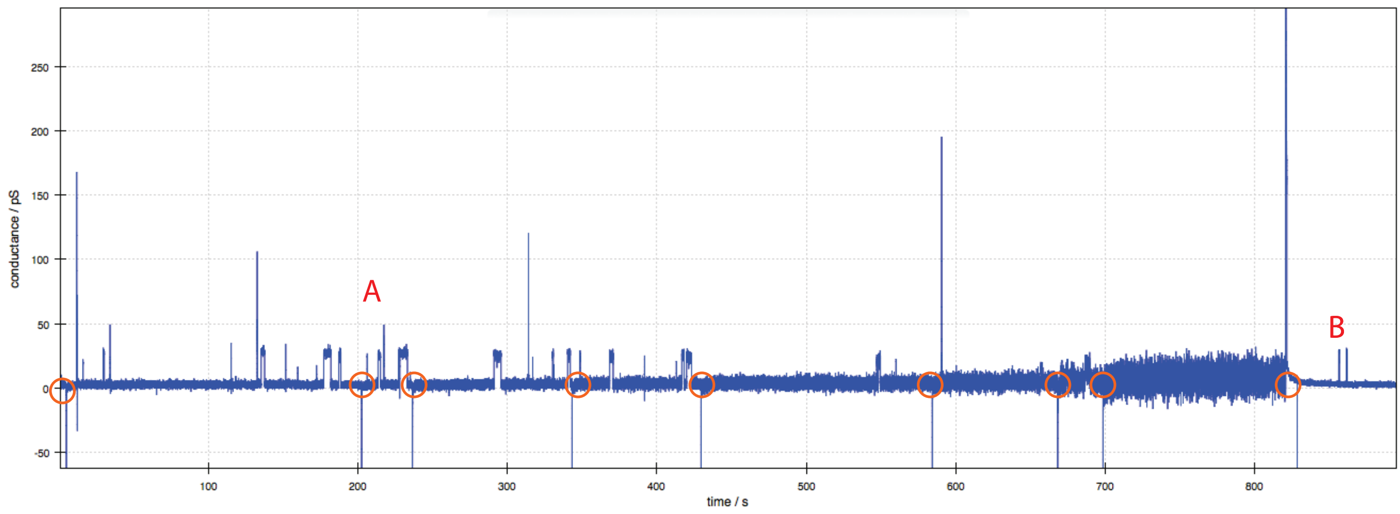
A
B
conductance / pS
time / s

A

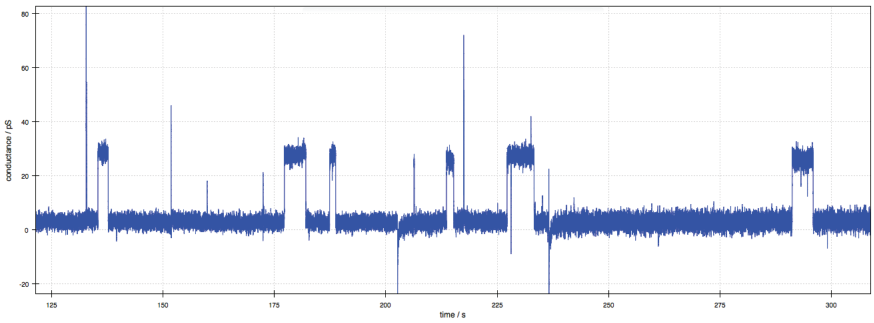
conductance / pS
time / s

B

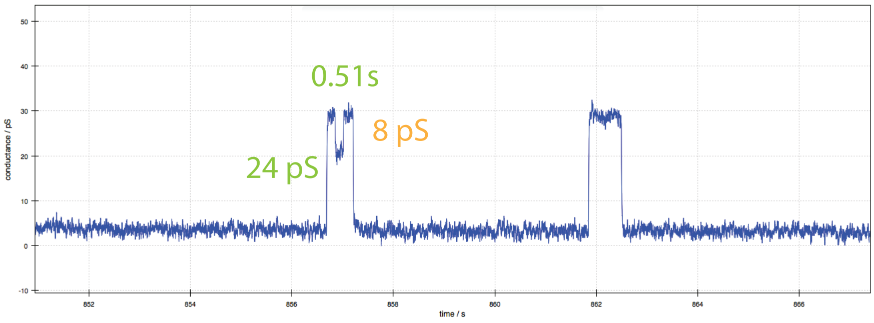
0.51s
8 pS
24 pS
conductance / pS
time / s

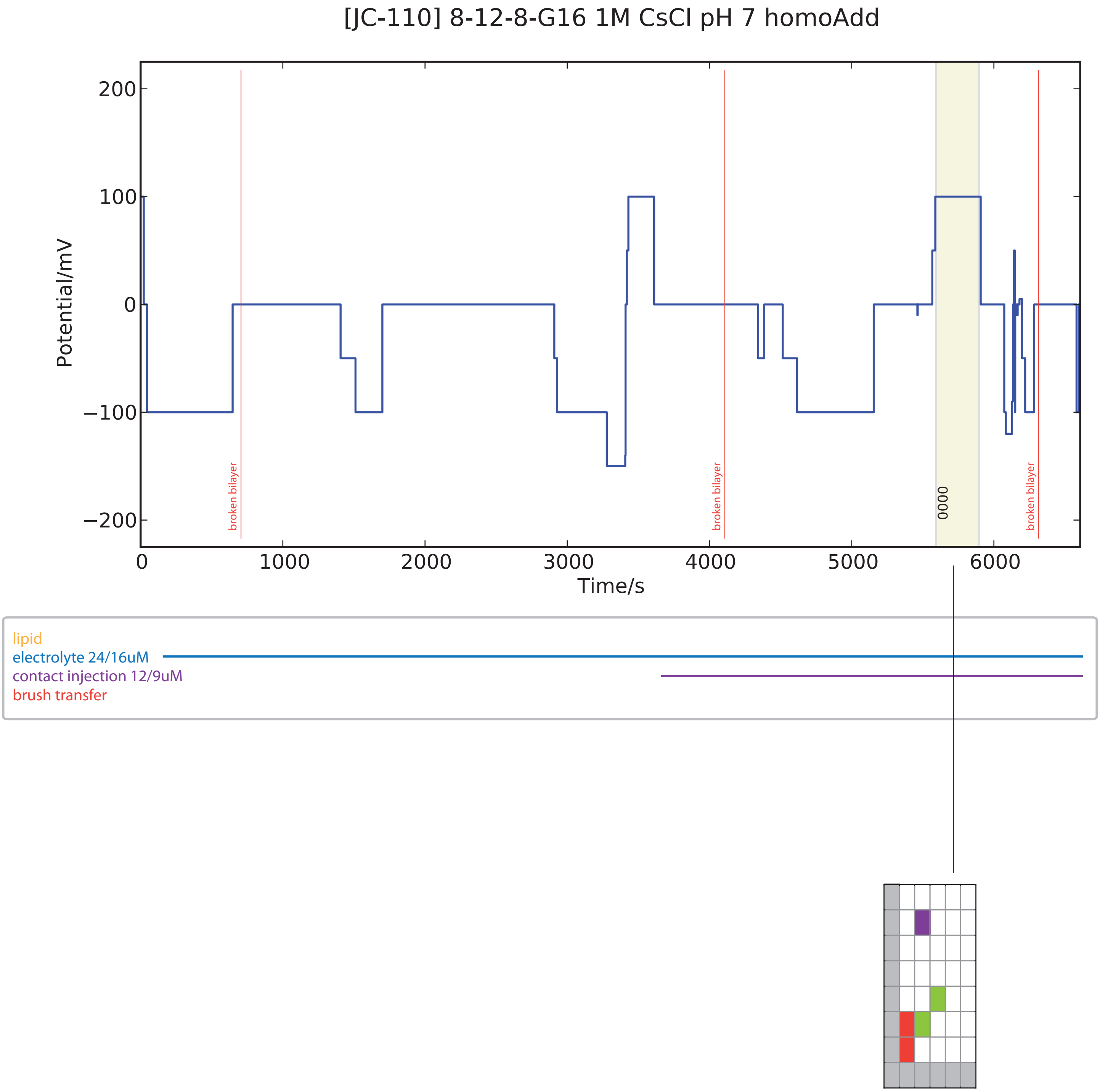
[JC-110] 8-12-8-G16 1M CsCl pH 7 homoAdd
Potential/mV
Time/s
200
100
0
−100
−200
0
1000
2000
3000
4000
5000
6000
broken bilayer
broken bilayer
0000
broken bilayer
lipid
electrolyte 24/16uM
contact injection 12/9uM
brush transfer

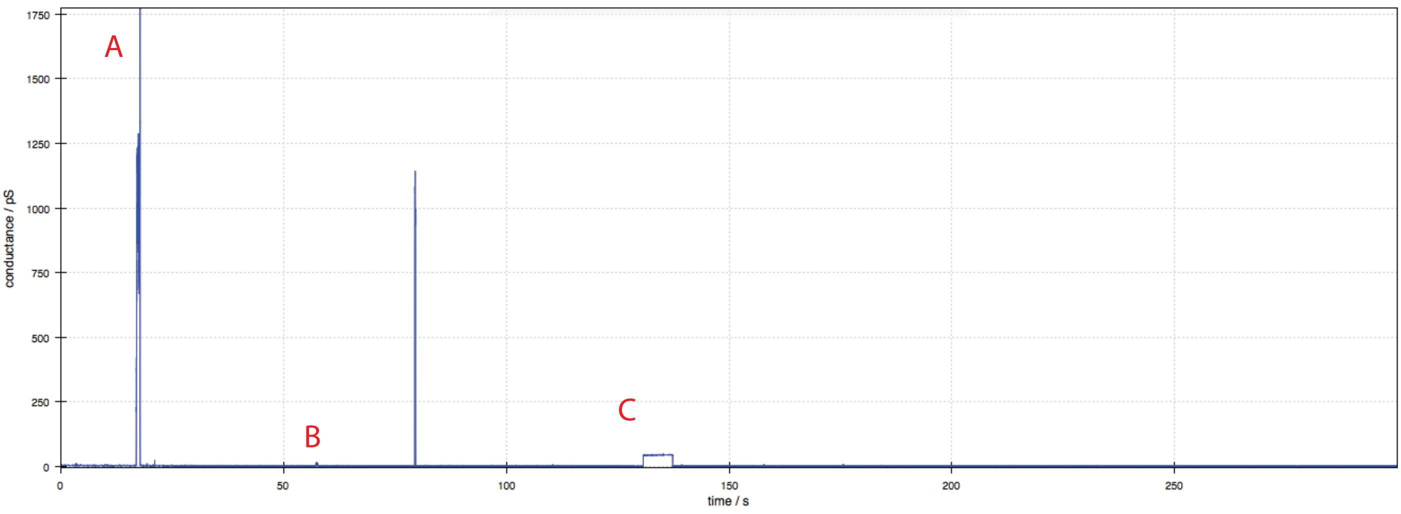
A
B
C
conductance / pS
1750
1500
1250
1000
750
500
250
0
0
50
100
150
200
250
time / s

A

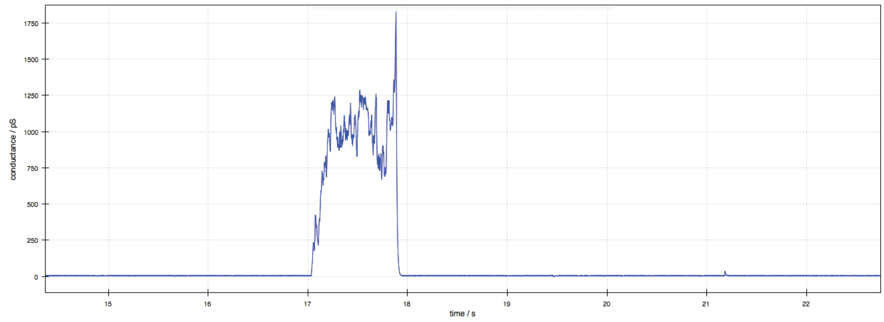
conductance / pS
time / s

B

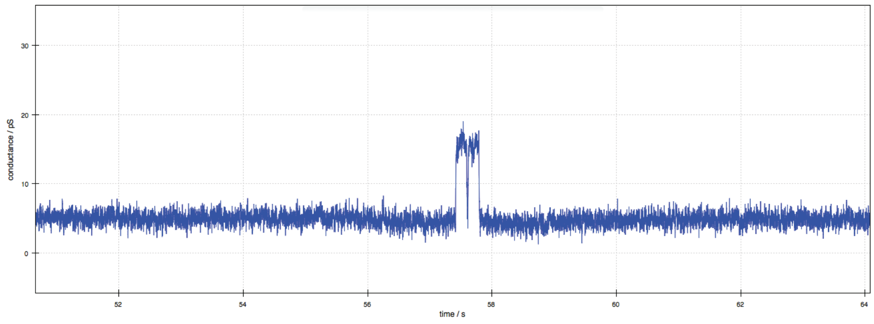
conductance / pS
time / s

C

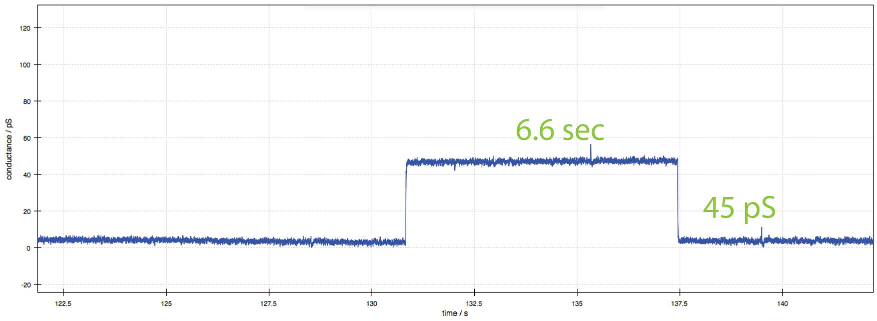
6.6 sec
45 pS
conductance / pS
time / s

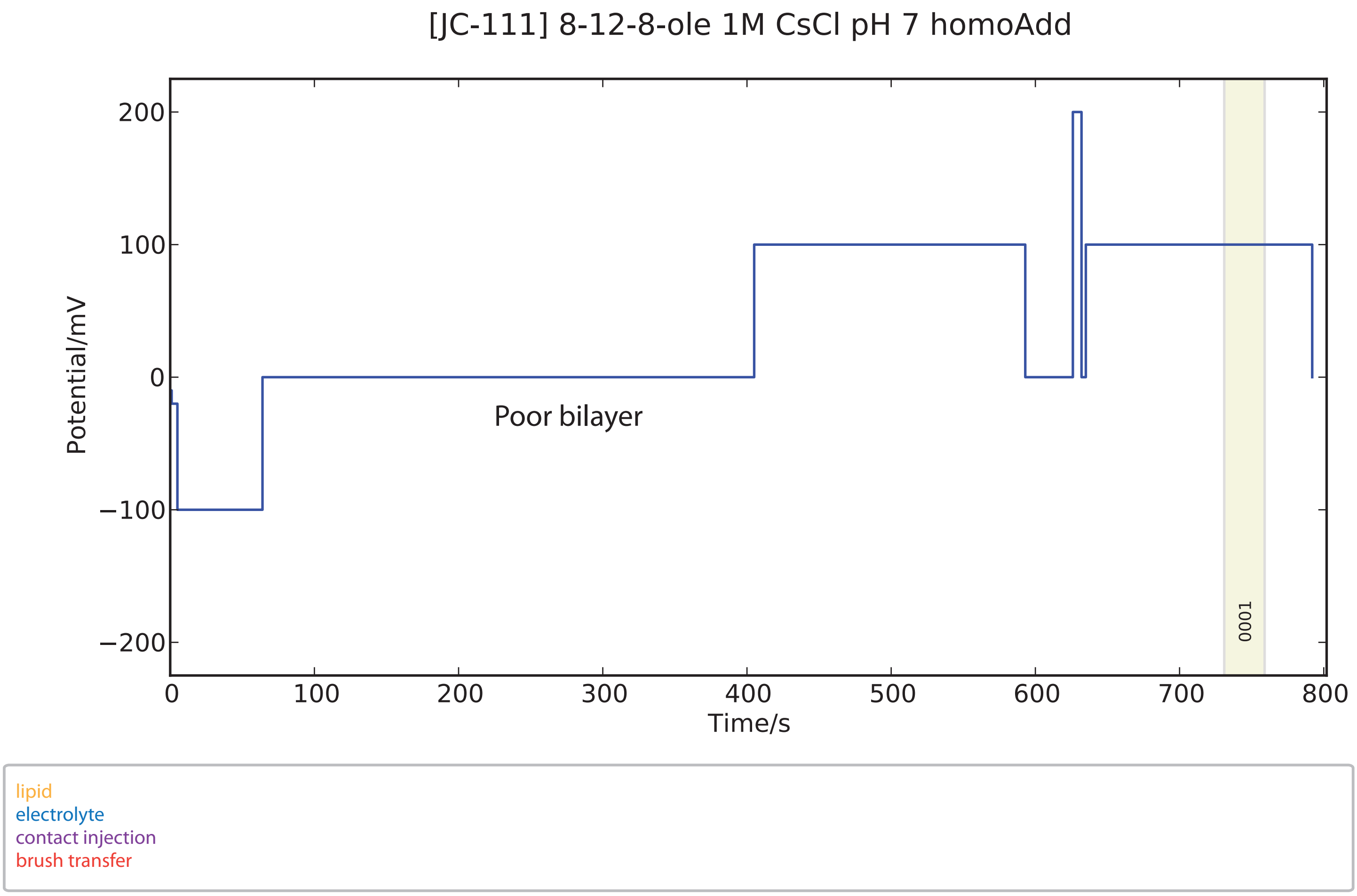
[JC-111] 8-12-8-ole 1M CsCl pH 7 homoAdd
Potential/mV
200
100
0
−100
−200
0
100
200
300
400
500
600
700
800
Time/s
Poor bilayer
0001
lipid
electrolyte
contact injection
brush transfer

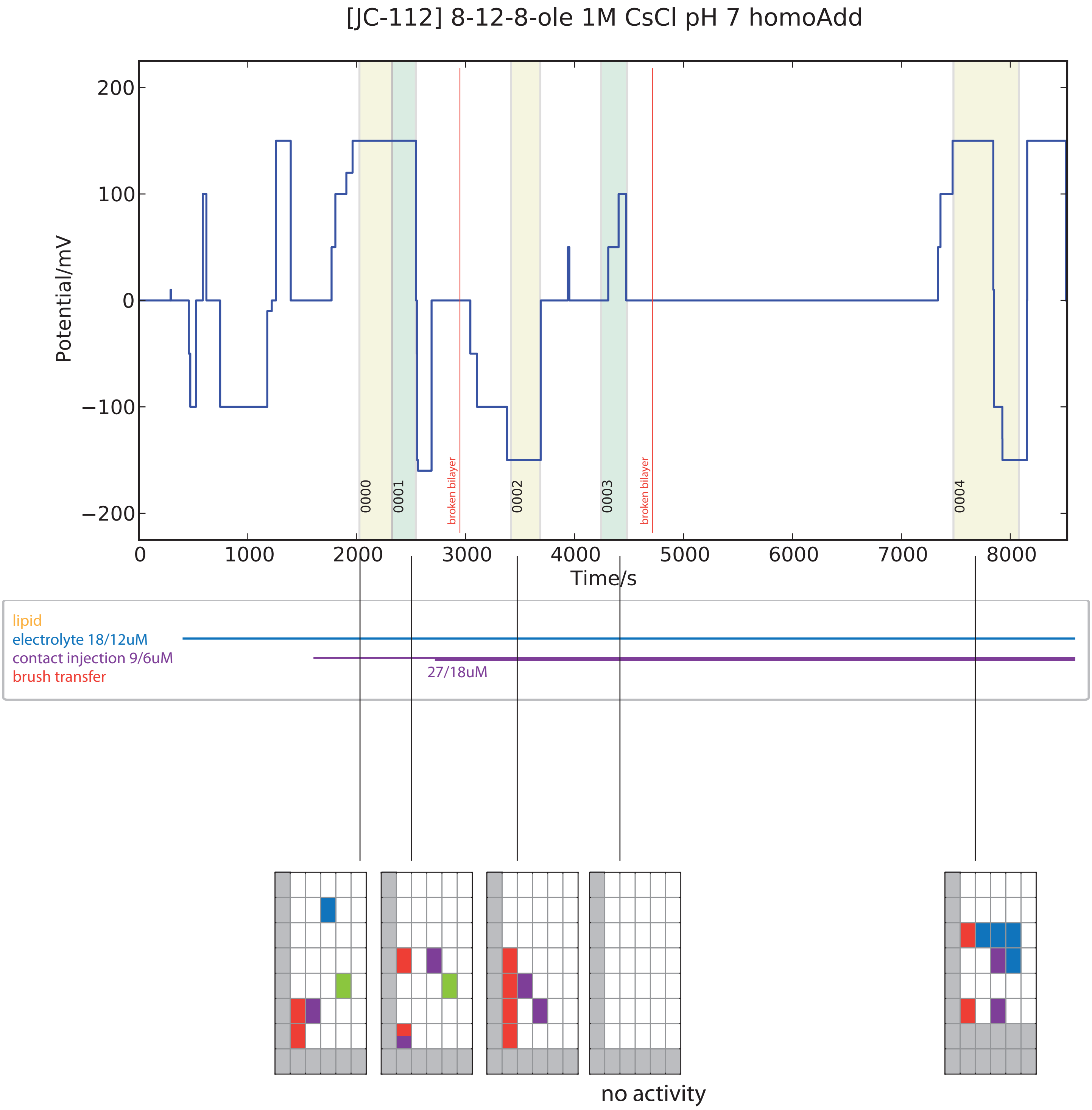

[JC-112] 8-12-8-ole 1M CsCl pH 7 homoAdd
Potential/mV
Time/s
0000
0001
broken bilayer
0002
0003
broken bilayer
0004
lipid
electrolyte 18/12uM
contact injection 9/6uM
brush transfer
27/18uM
no activity

112-0000

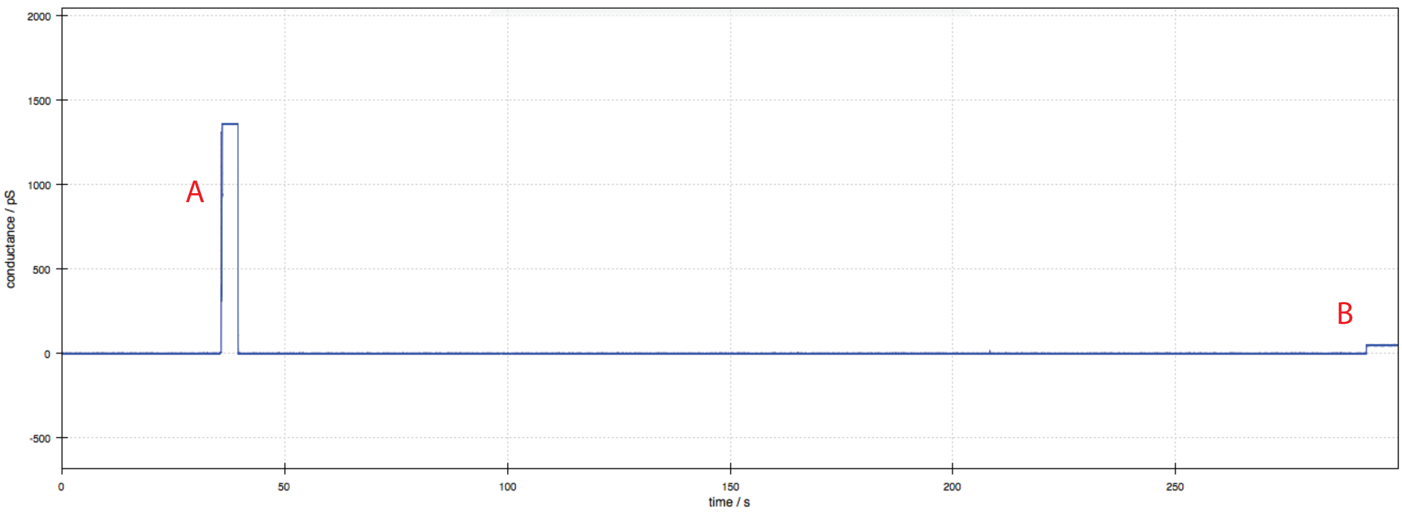

A
B
conductance / pS
time / s

A

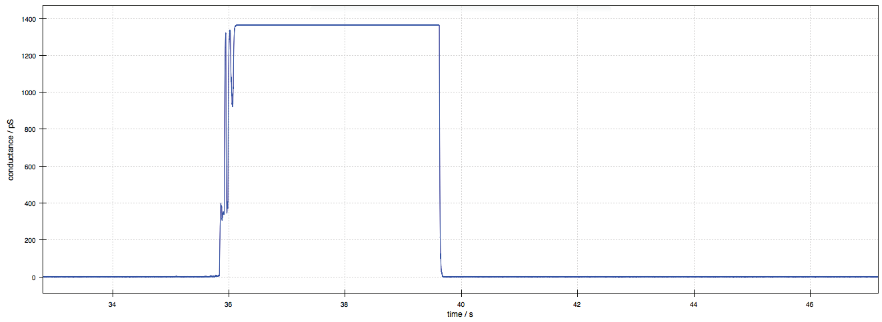

conductance / pS
time / s

B

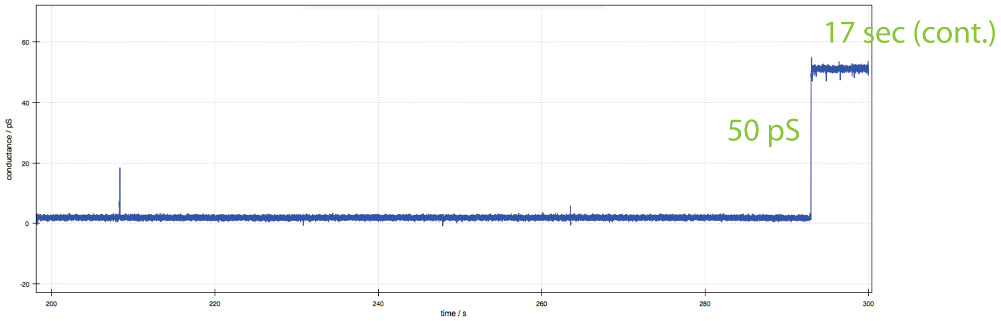

17 sec (cont.)
50 pS
conductance / pS
time / s

112-0001

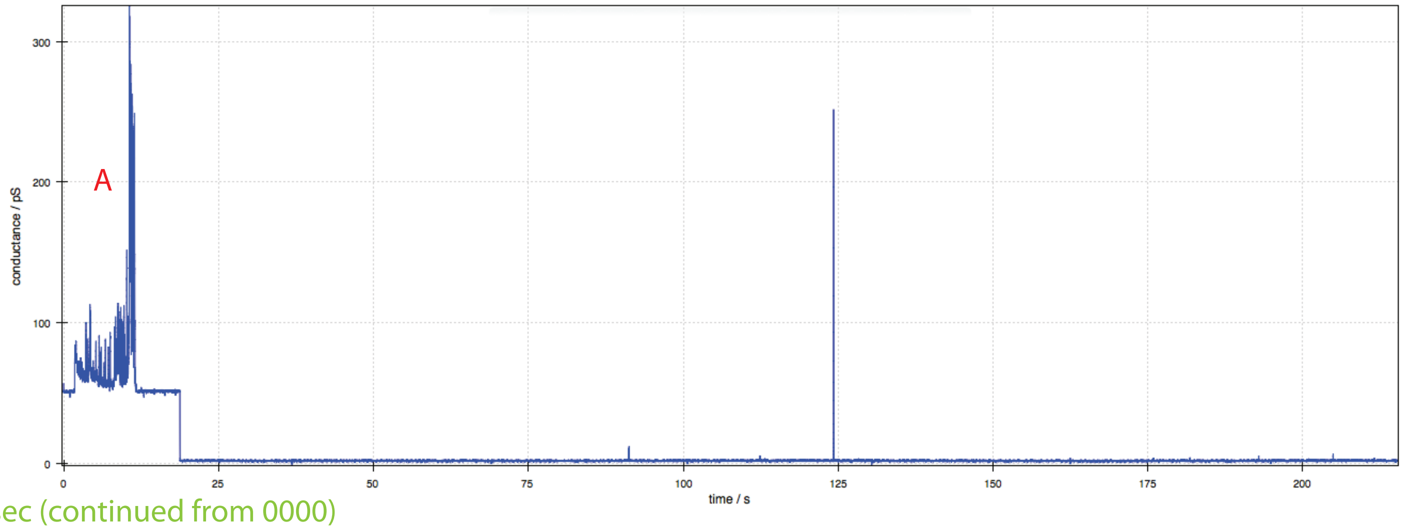

17 sec (continued from 0000)

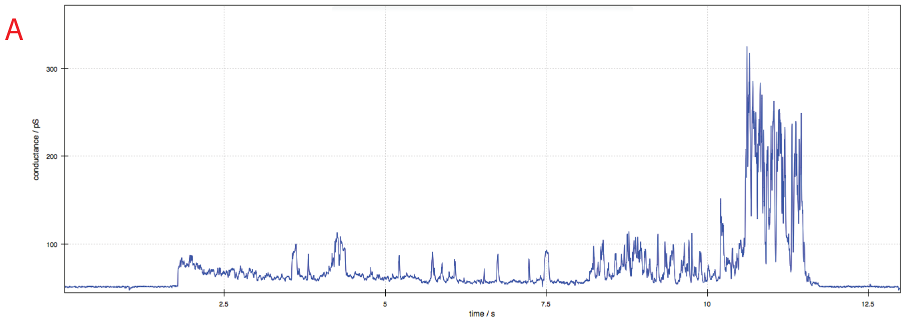

112-0003

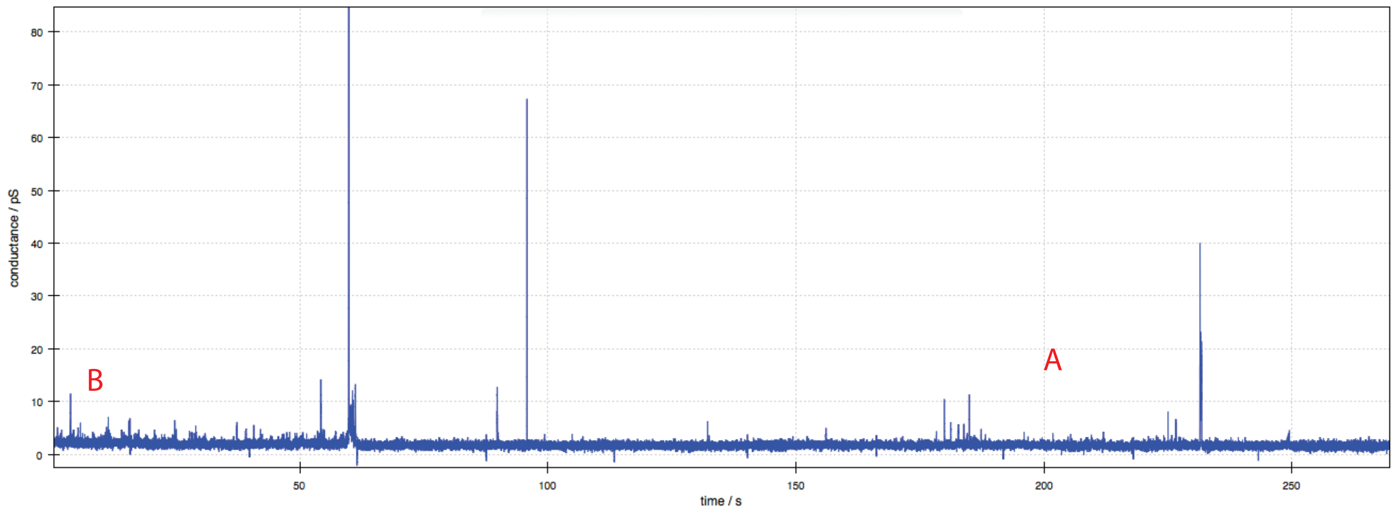

A

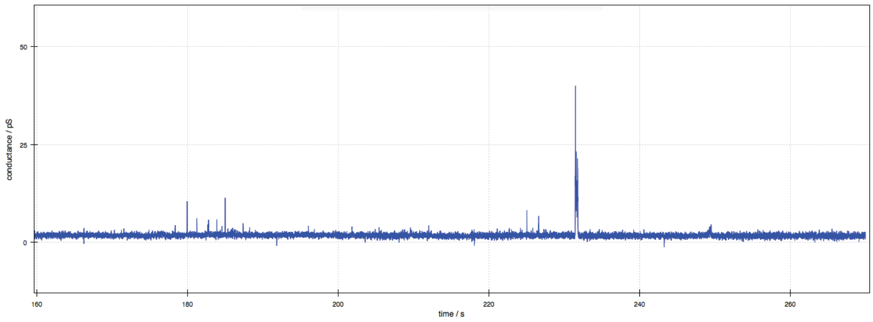

B

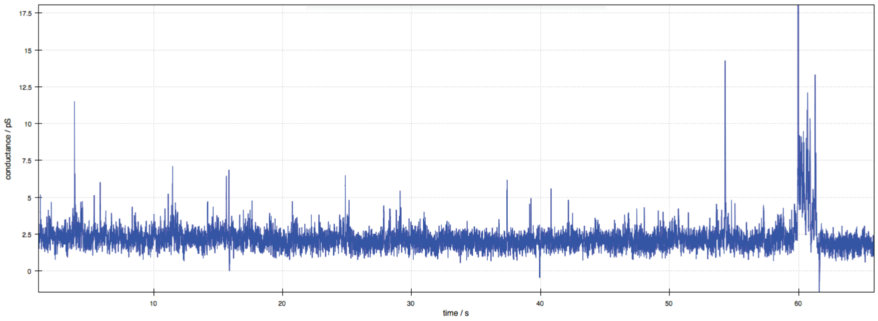

112-0003

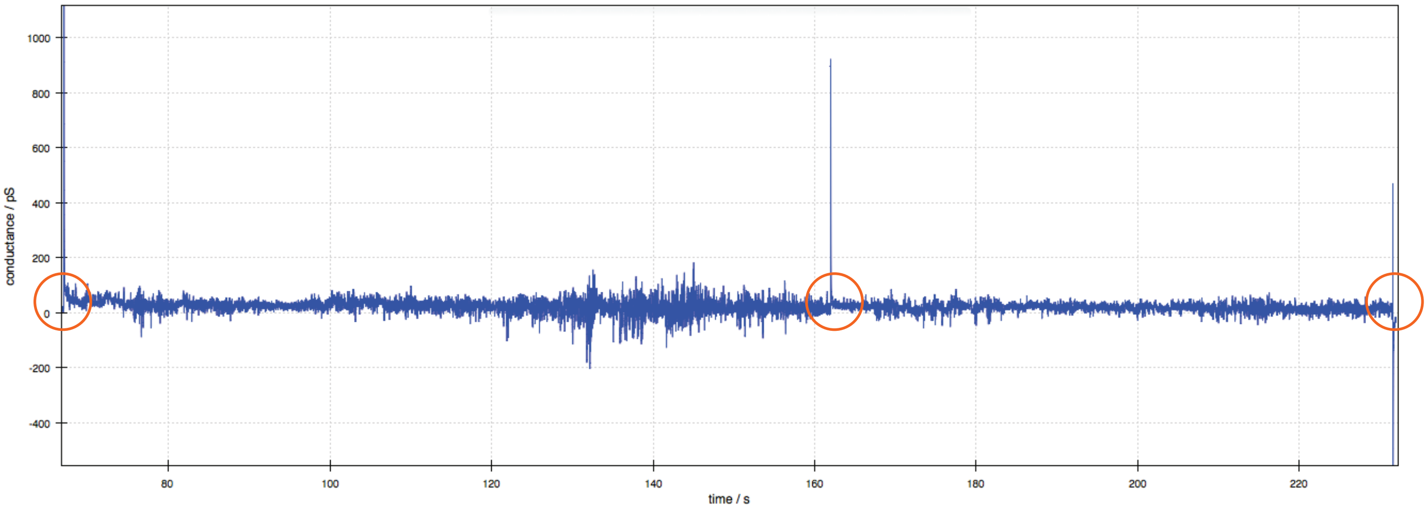

112-0004

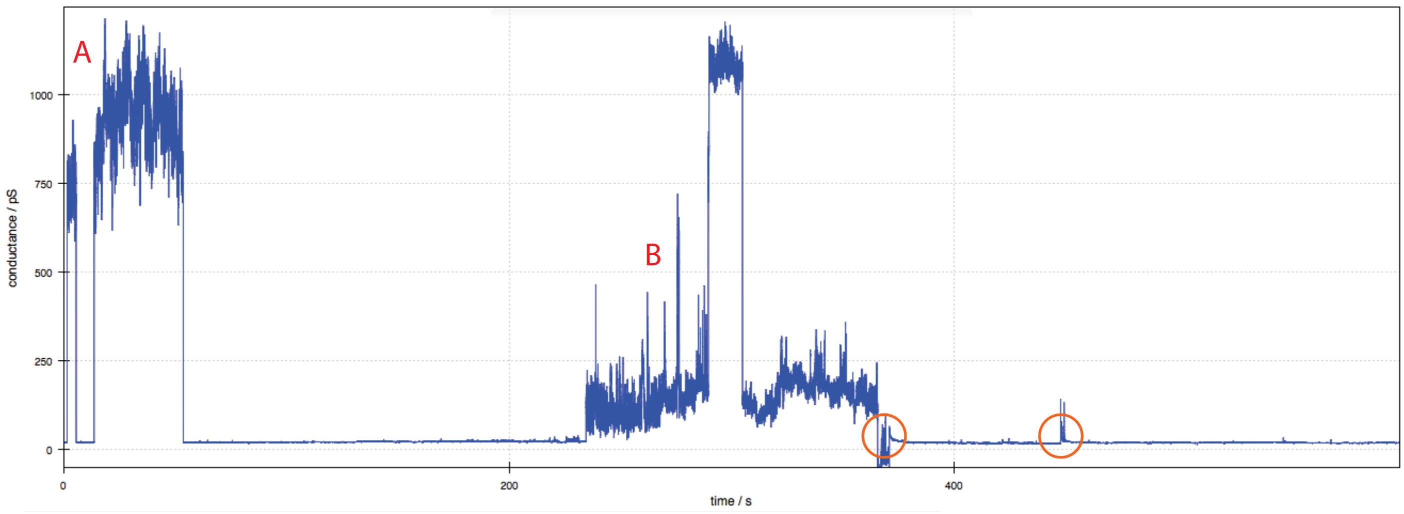
A
B
1000
750
500
250
0
conductance / pS
0
200
400
time / s

A

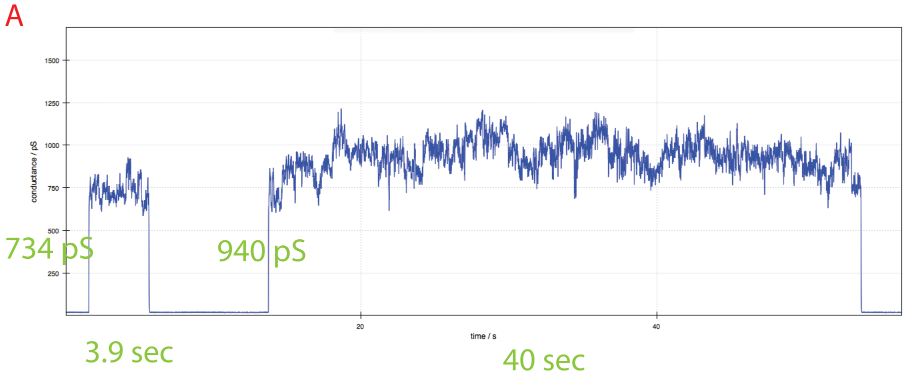
1500
1250
1000
750
500
250
conductance / pS
734 pS
940 pS
20
40
time / s
3.9 sec
40 sec

B

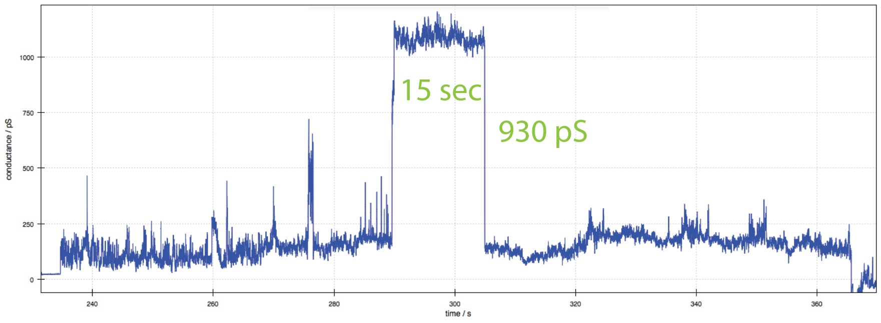
1000
750
500
250
0
conductance / pS
15 sec
930 pS
240
260
280
300
320
340
360
time / s

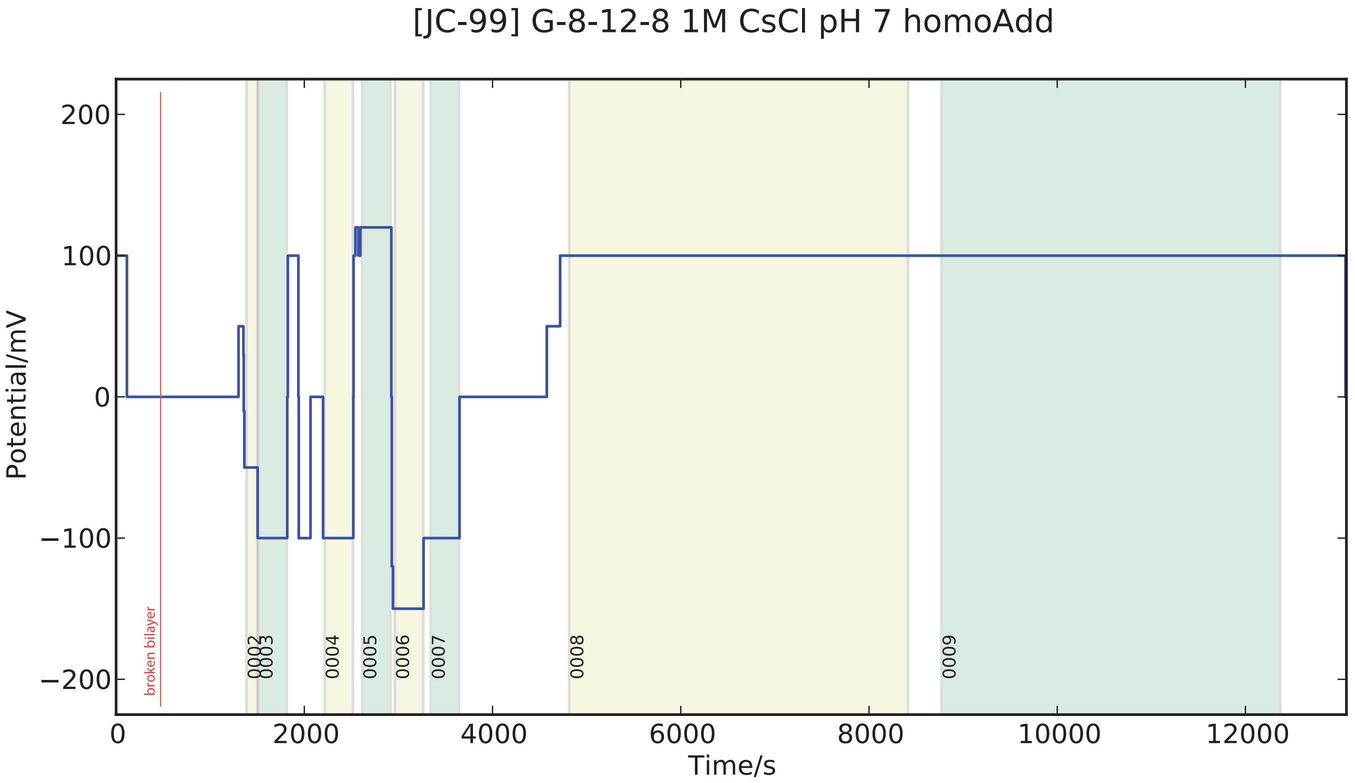

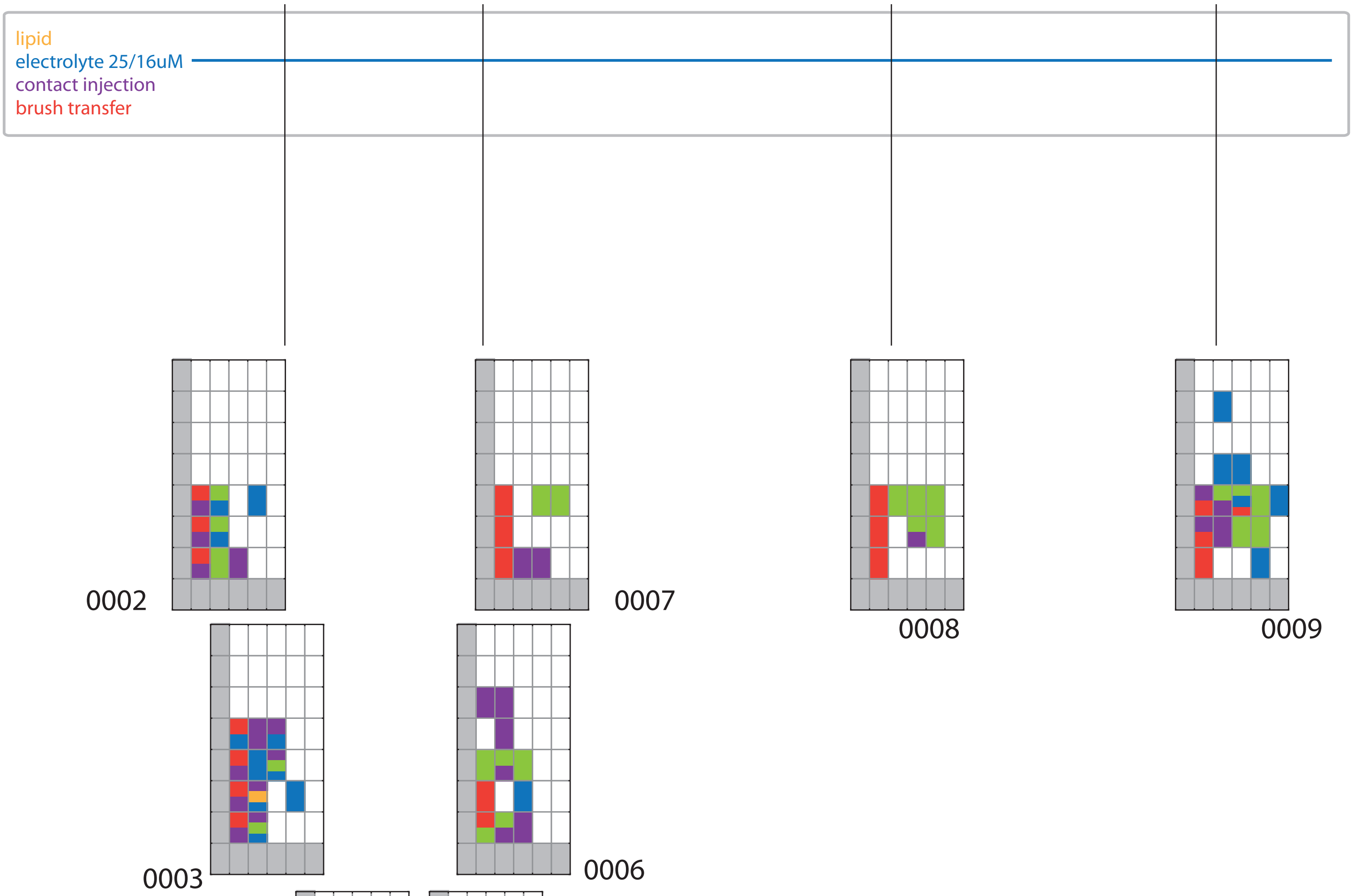

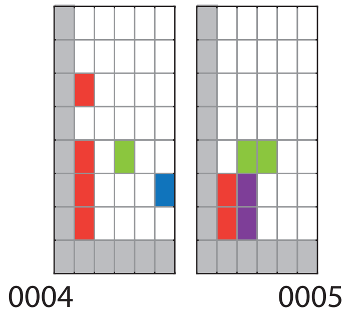

I think it is fair to conclude that:

- both 8-12-8-G(12) and G(12)-8-12-8 can form similar discrete channels,
- G(12)-8-12-8 has a variety of other membrane-active modes,
- and with weak evidence we'll say that G(12)-8-12-8 forms these discrete channels more readily.

99-0002

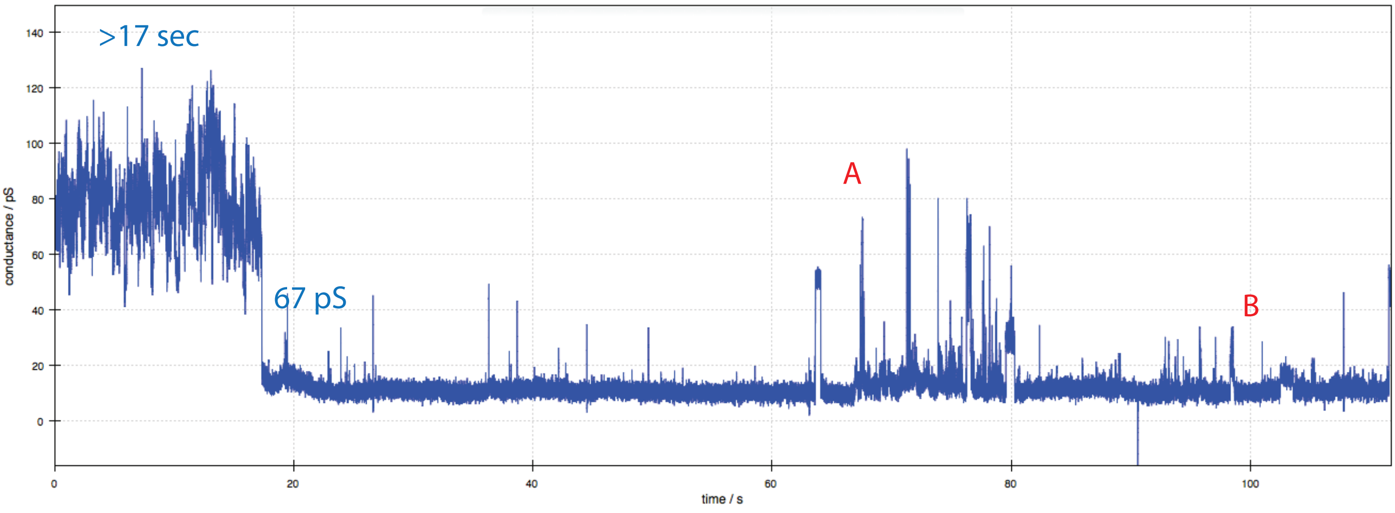
>17 sec
67 pS
A
B
conductance / pS
time / s
140
120
100
80
60
40
20
0
20
40
60
80
100

A

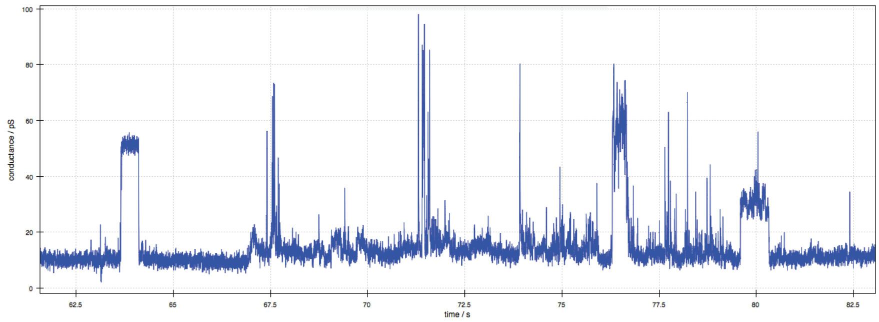
conductance / pS
time / s

B

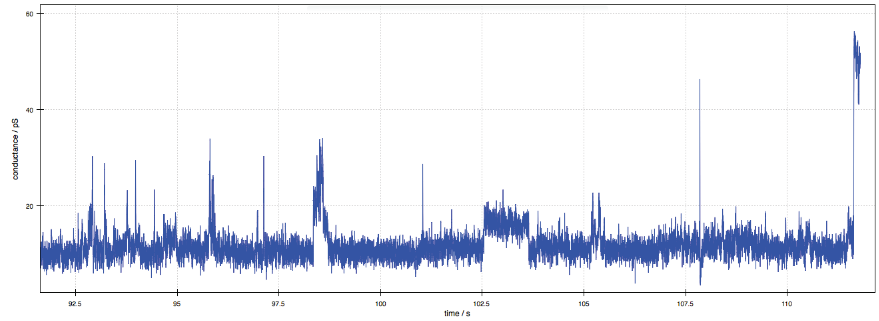
conductance / pS
time / s

99-0003

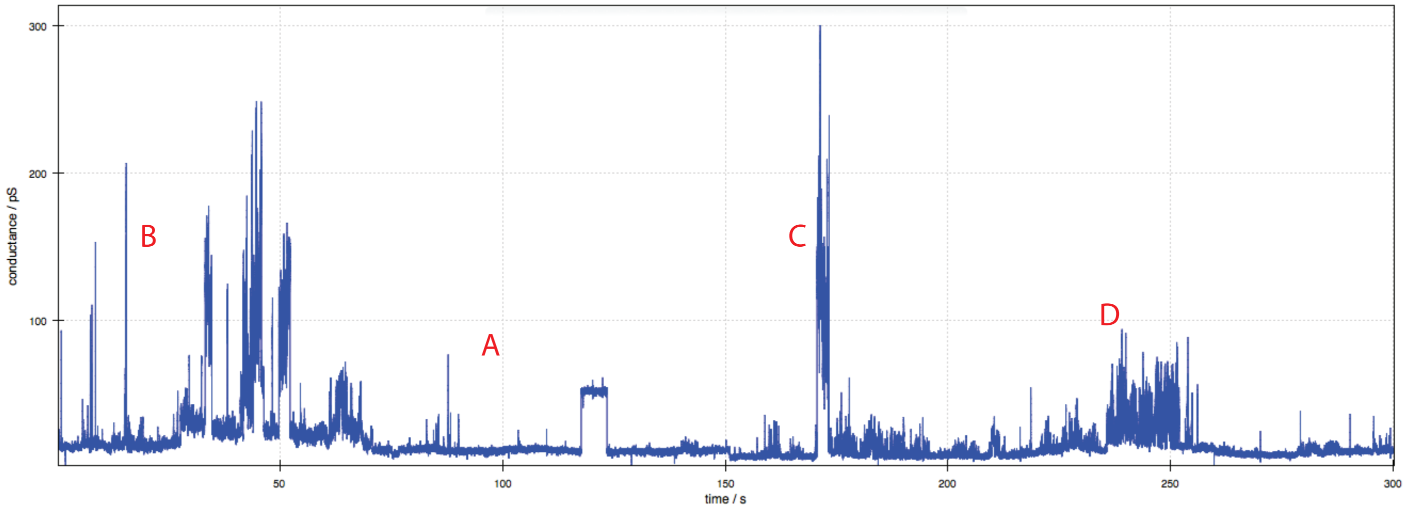
conductance / pS
time / s
B
A
C
D

A

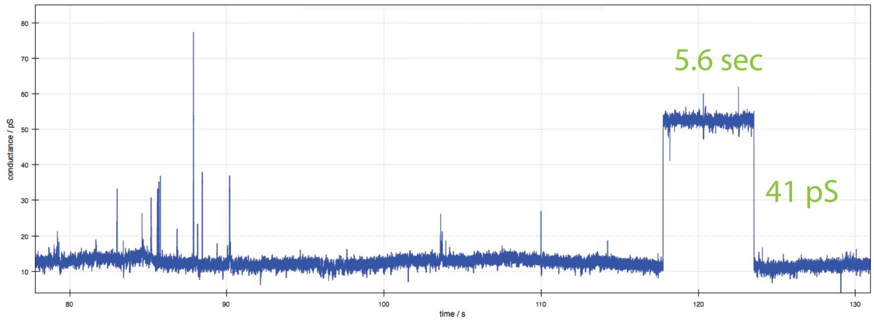
5.6 sec
41 pS
conductance / pS
time / s

B

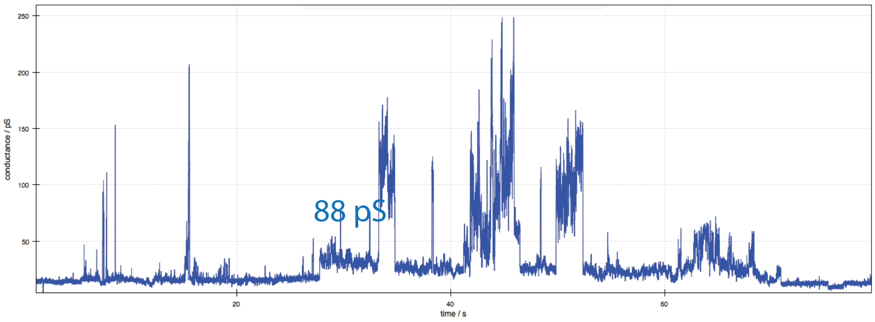
88 pS
conductance / pS
time / s

C

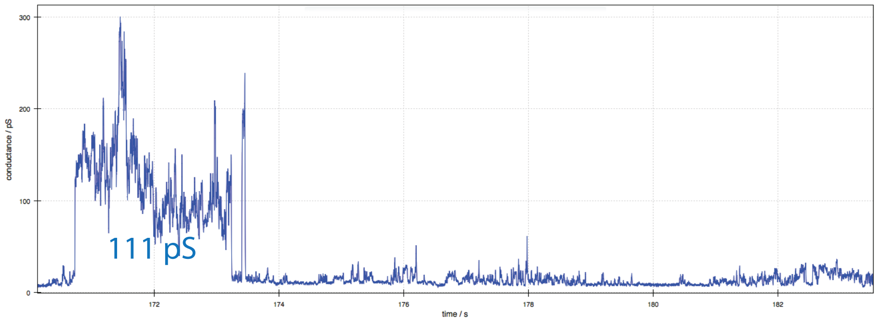
111 pS
conductance / pS
time / s

D

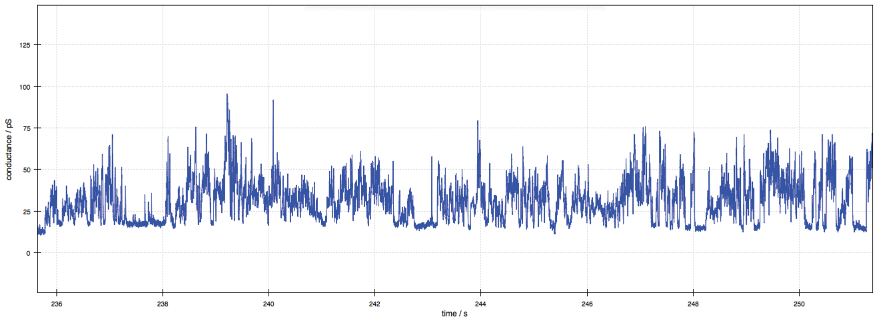
conductance / pS
time / s

99-0004

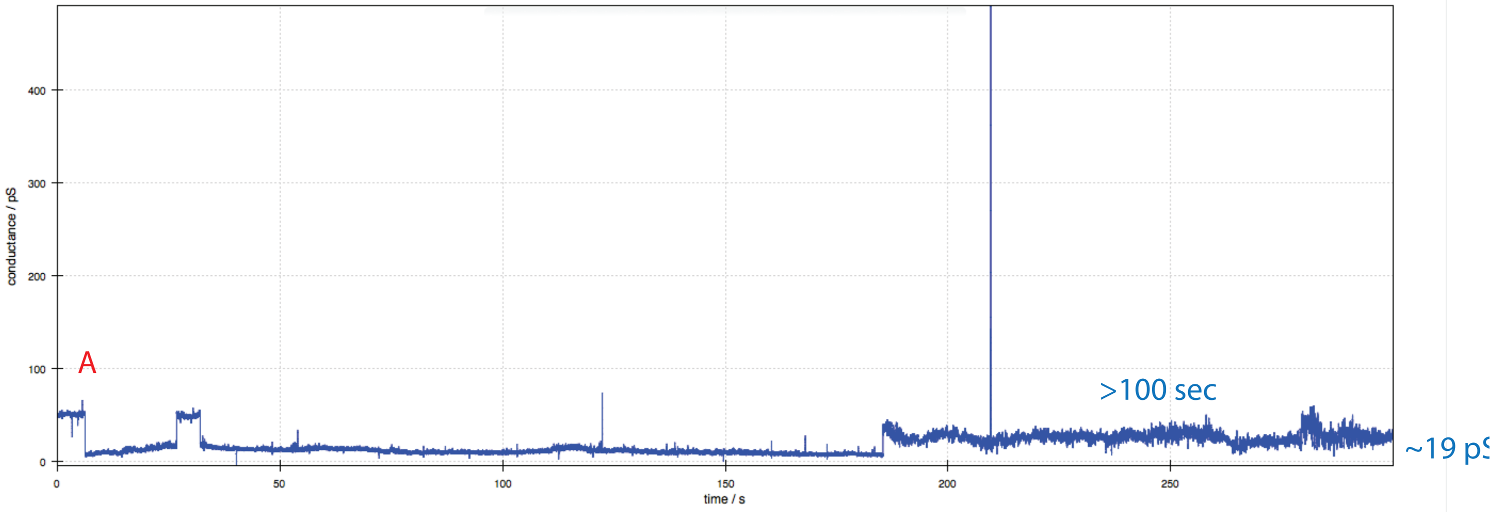

A

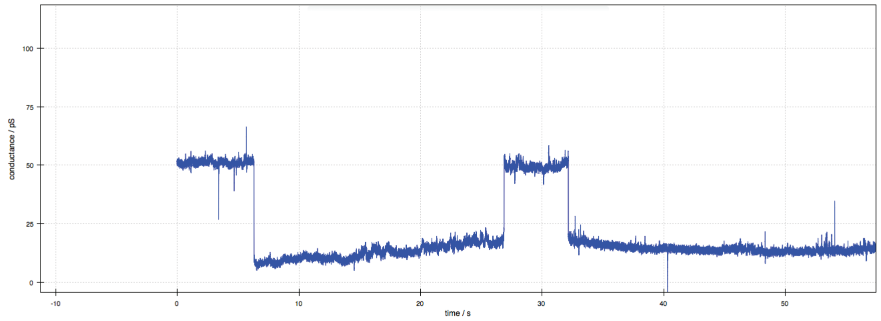

99-0005

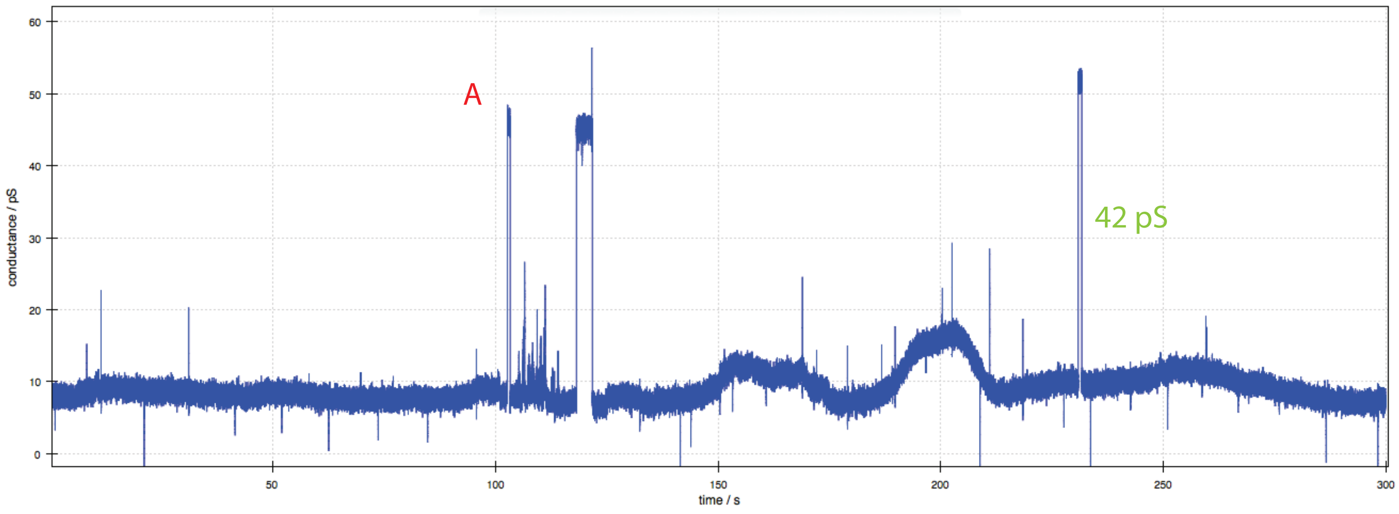

A

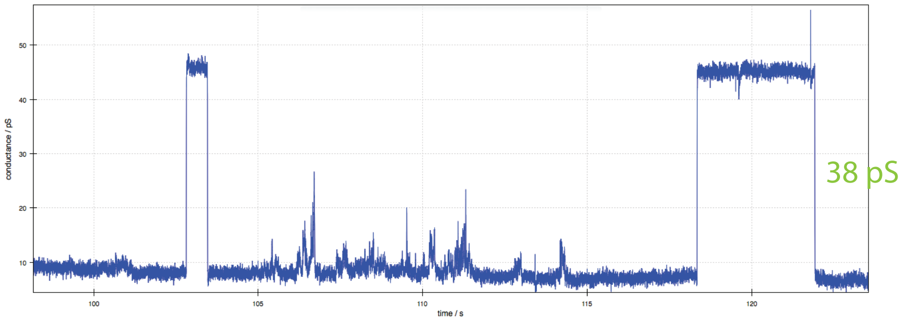

99-0006

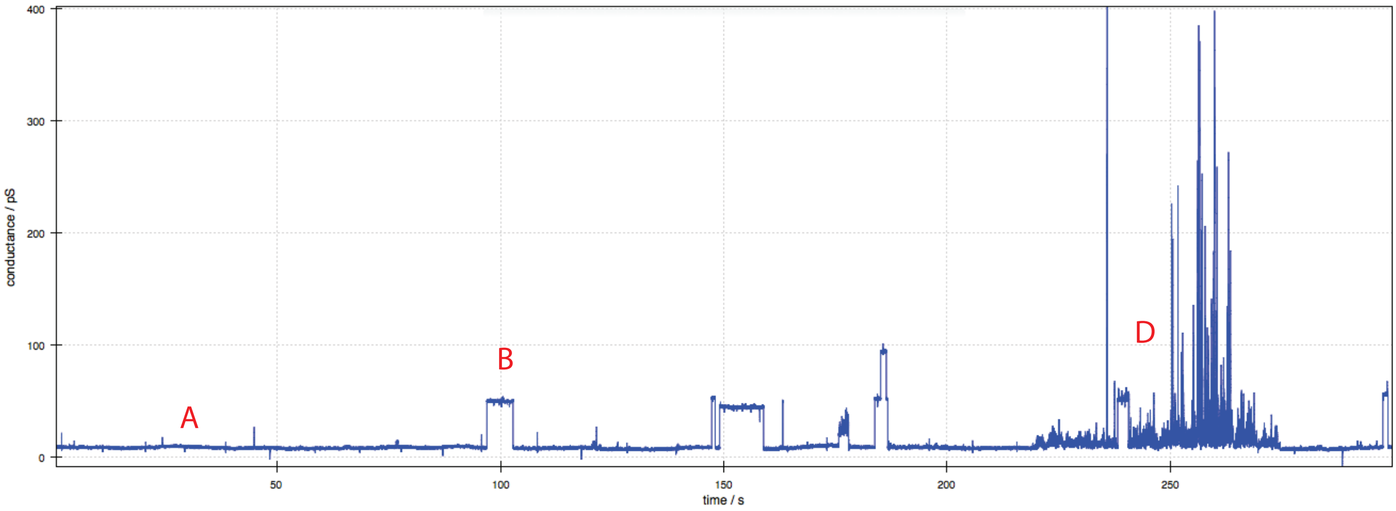

conductance / pS
time / s
A
B
D

A

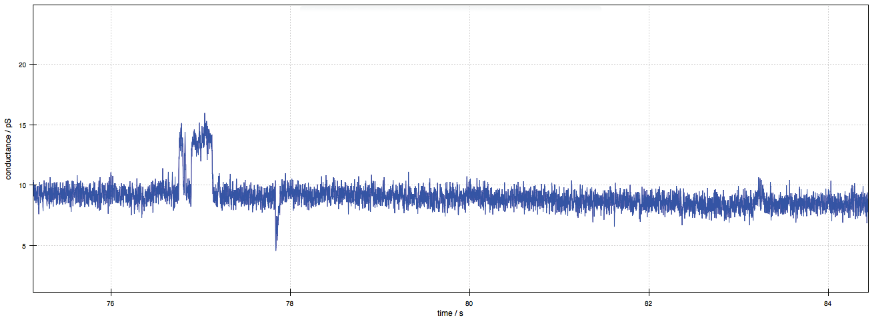

conductance / pS
time / s

B

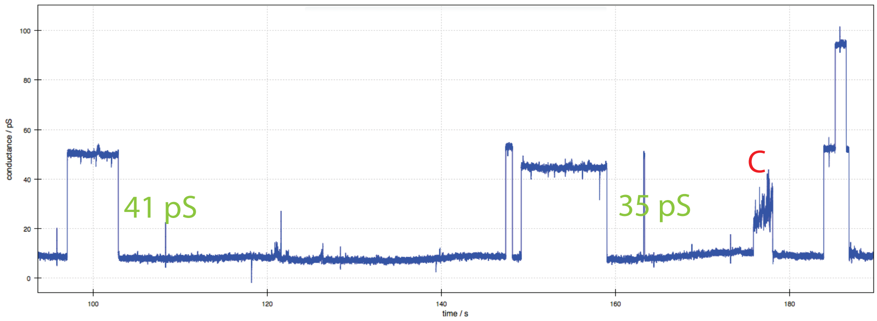

conductance / pS
time / s
41 pS
35 pS
C

C

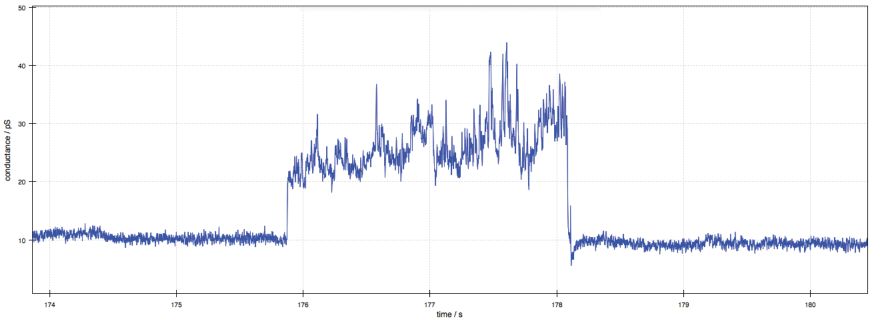

conductance / pS
time / s

D

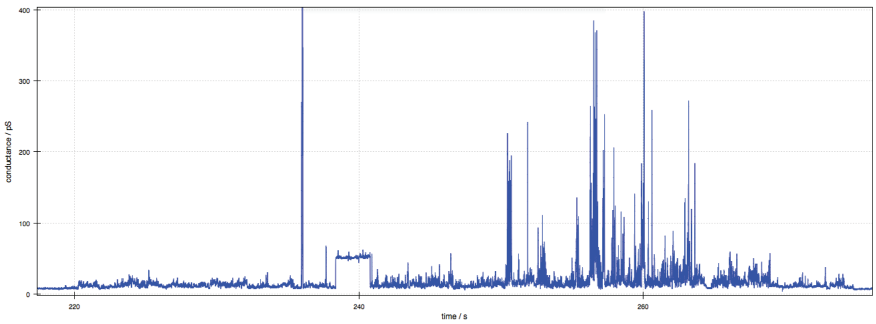

conductance / pS
time / s

99-0007

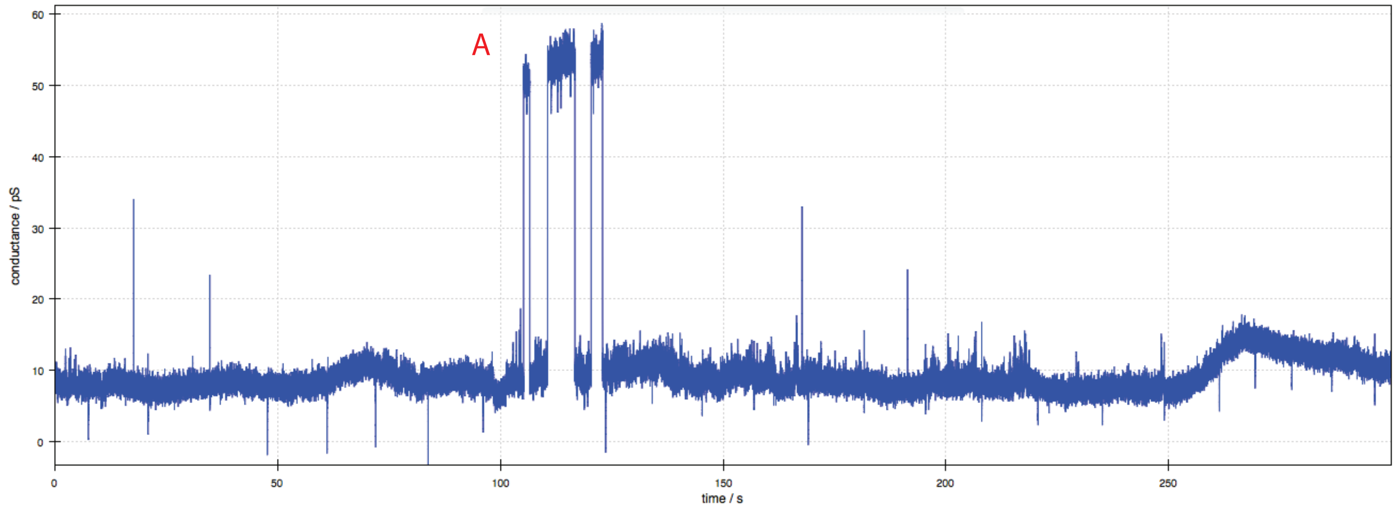
A
60
50
40
30
20
10
0
conductance / pS
0
50
100
150
200
250
time / s

A

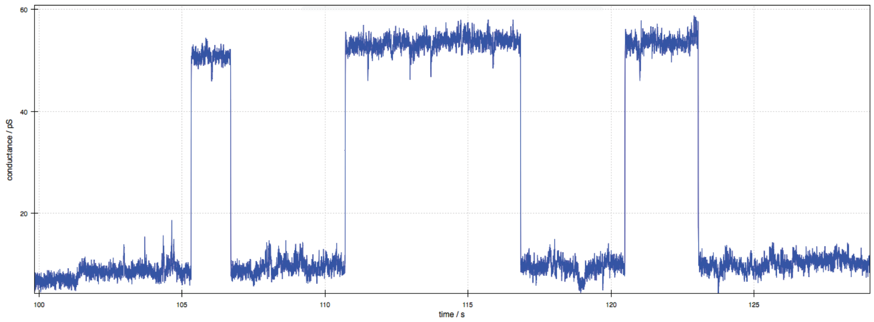
60
40
20
conductance / pS
100
105
110
115
120
125
time / s

99-0008

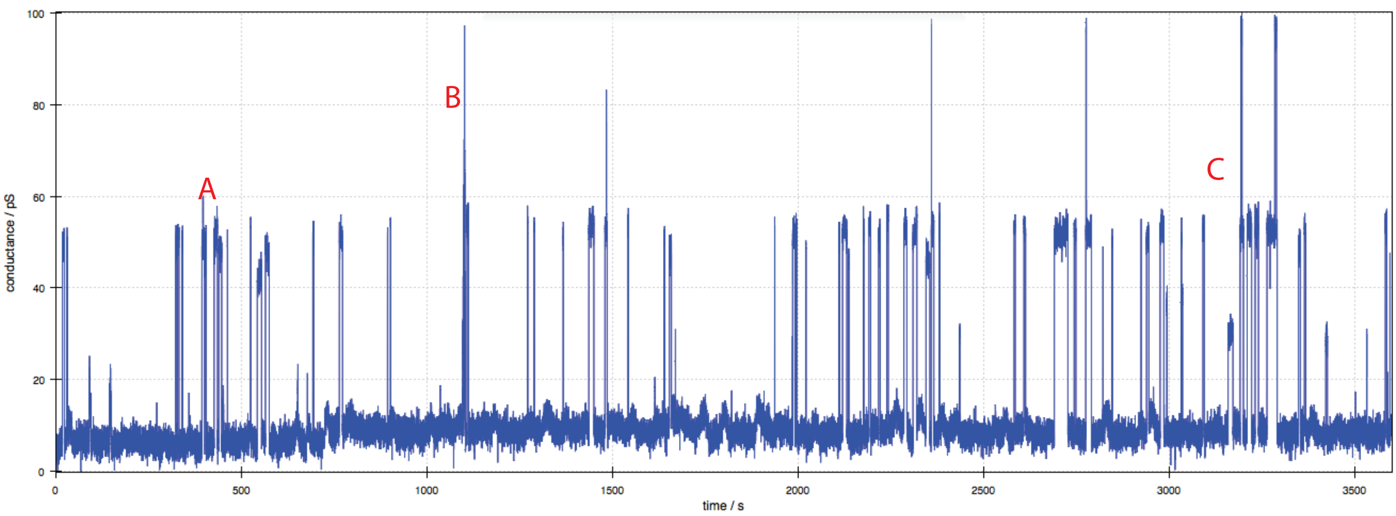

conductance / pS
time / s
0
20
40
60
80
100
0
500
1000
1500
2000
2500
3000
3500
A
B
C

A

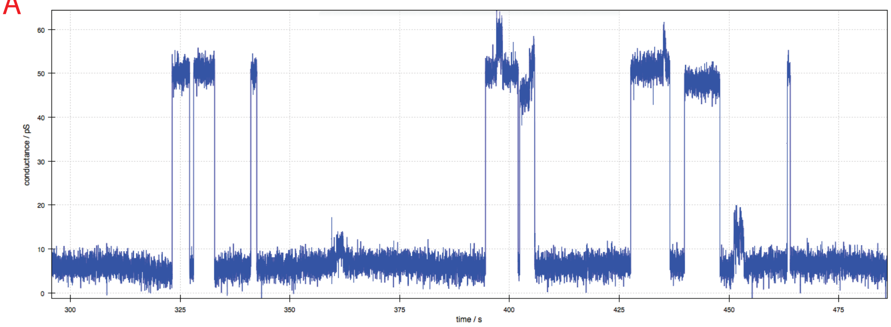

conductance / pS
time / s
0
10
20
30
40
50
60
300
325
350
375
400
425
450
475

B

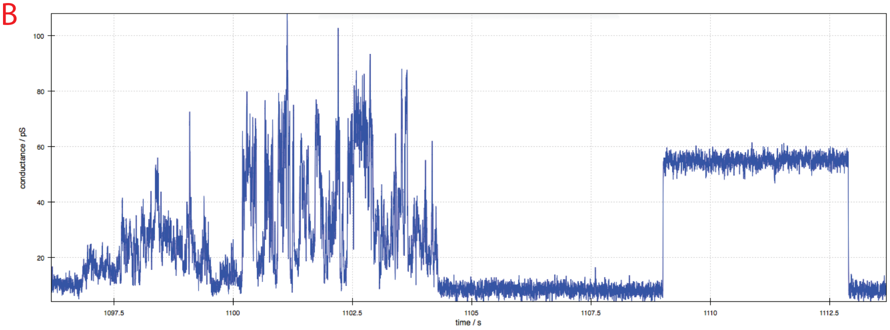

conductance / pS
time / s
20
40
60
80
100
1097.5
1100
1102.5
1105
1107.5
1110
1112.5

C

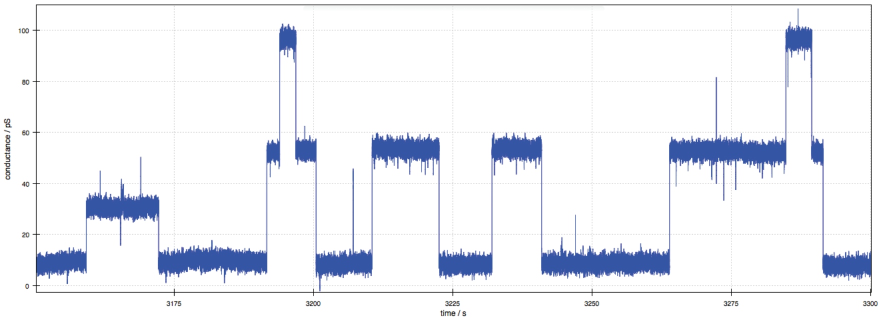

conductance / pS
time / s
0
20
40
60
80
100
3175
3200
3225
3250
3275
3300

99-0009

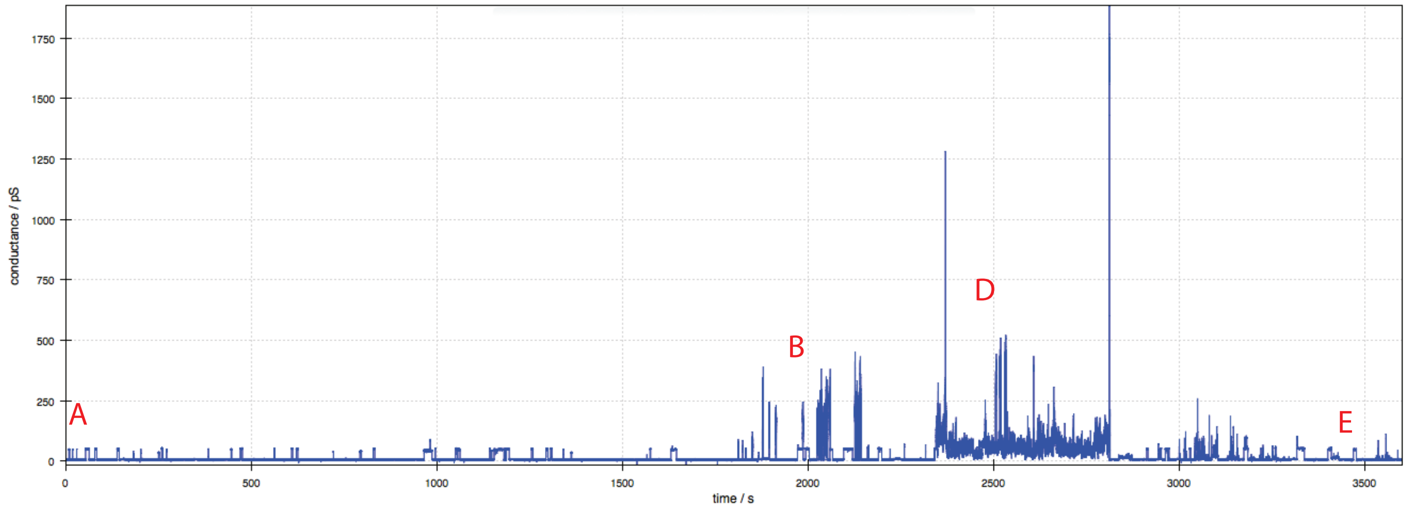
conductance / pS
time / s
A
B
D
E

A

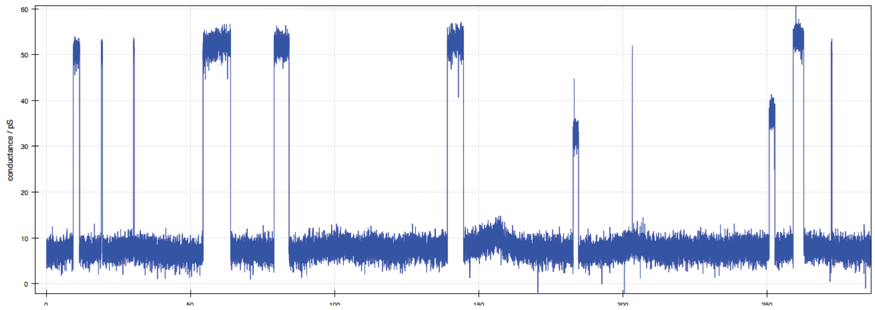
conductance / pS

B

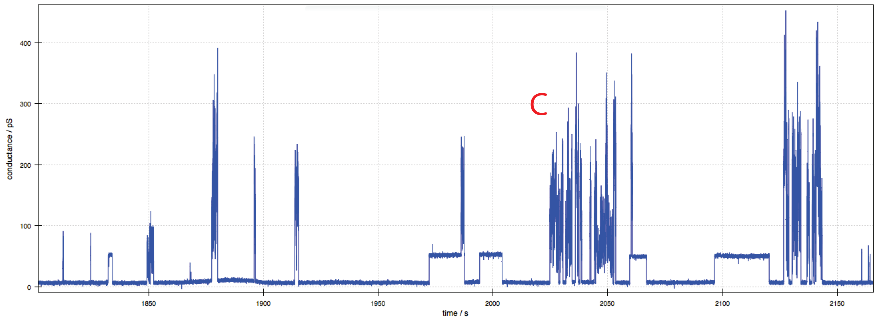
conductance / pS
time / s
C

C

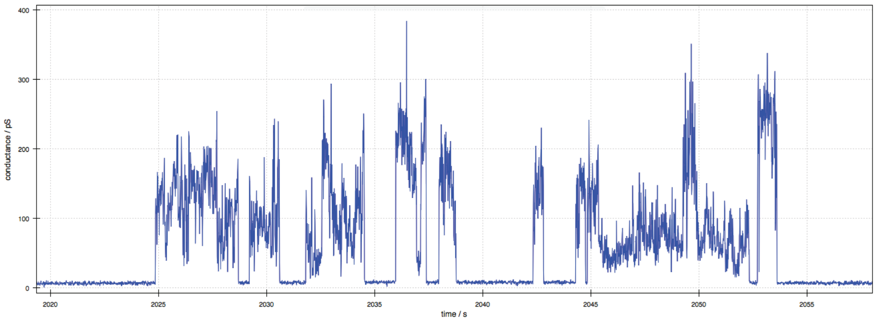
conductance / pS
time / s

D

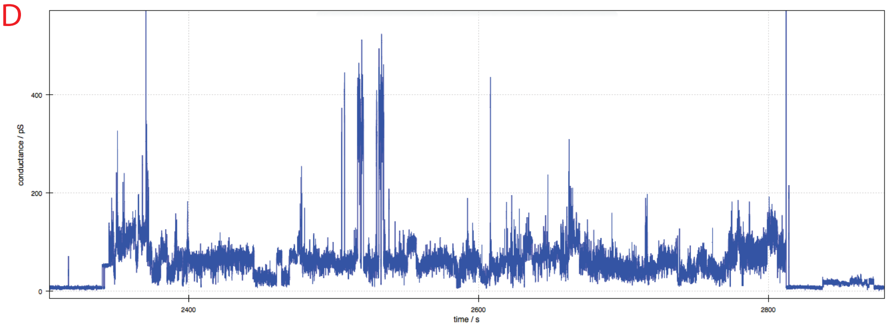

400
200
0
conductance / pS
2400
2600
2800
time / s

E

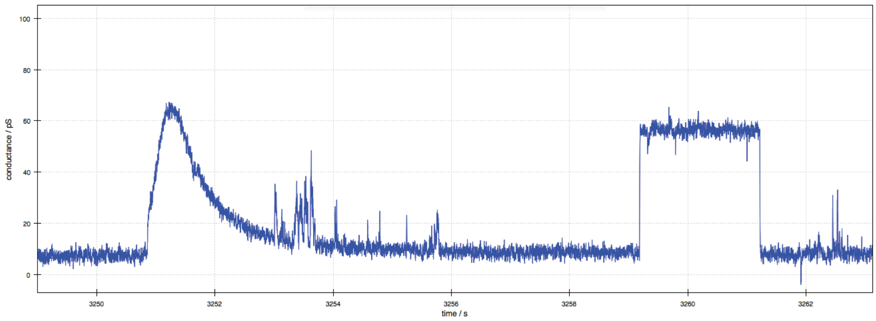

100
80
60
40
20
0
conductance / pS
3250
3252
3254
3256
3258
3260
3262
time / s

2 40-0001 1M CsCl inj., +100mV

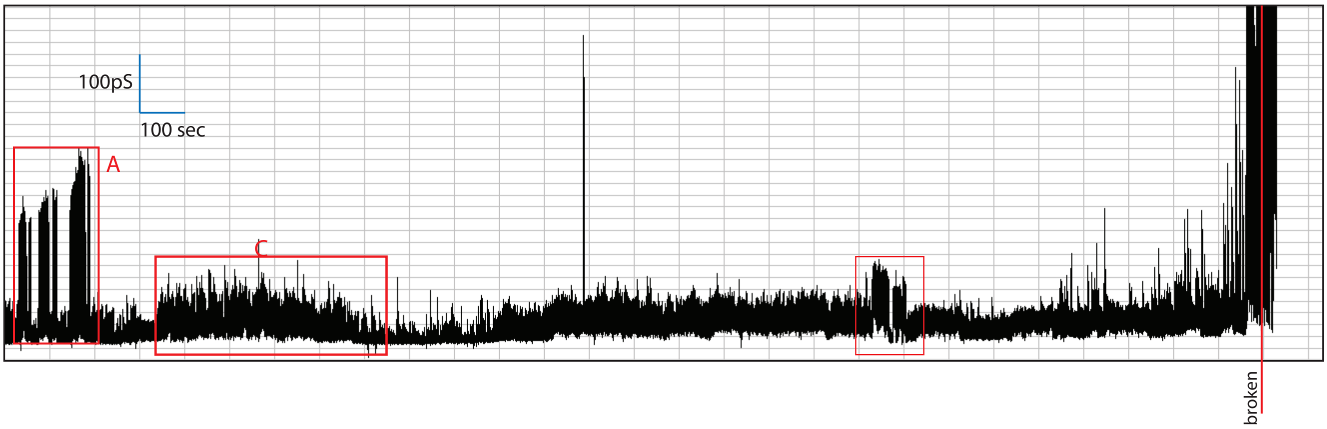

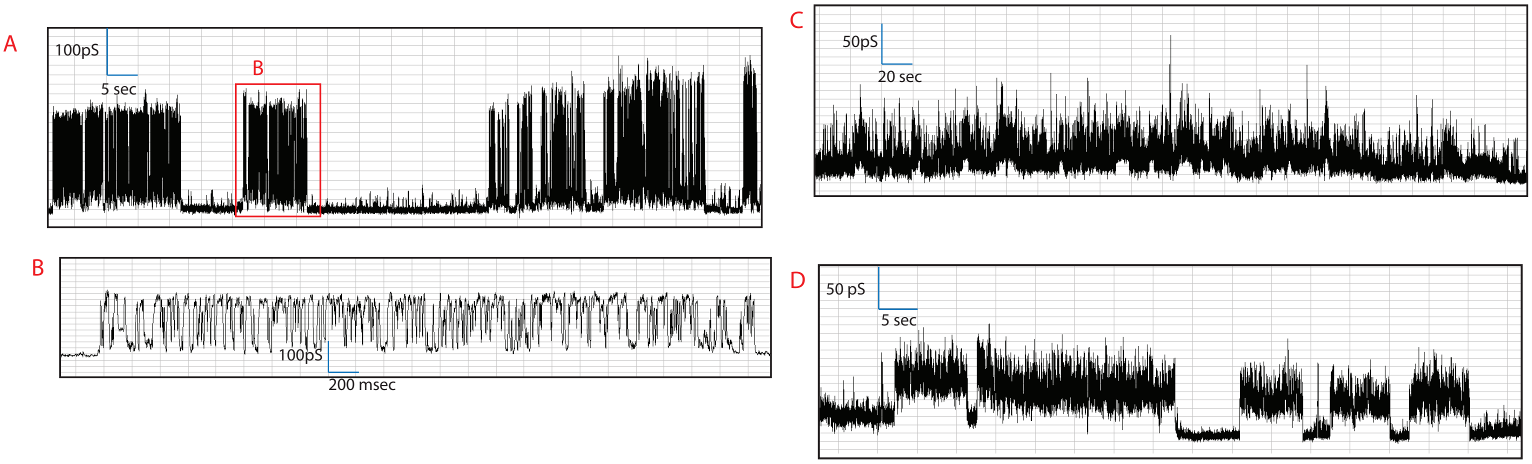

2 40-0002 1M CsCl inj., +120mV

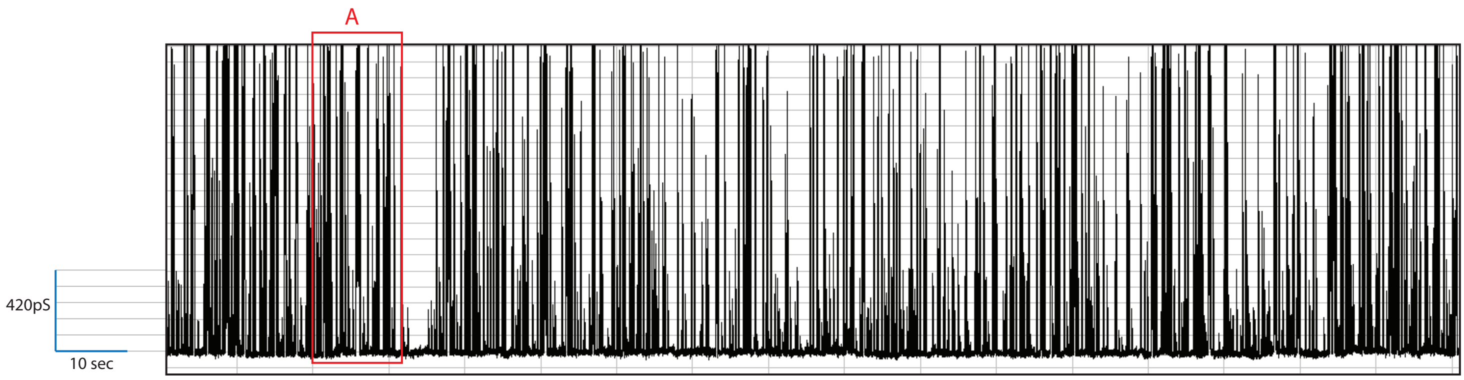

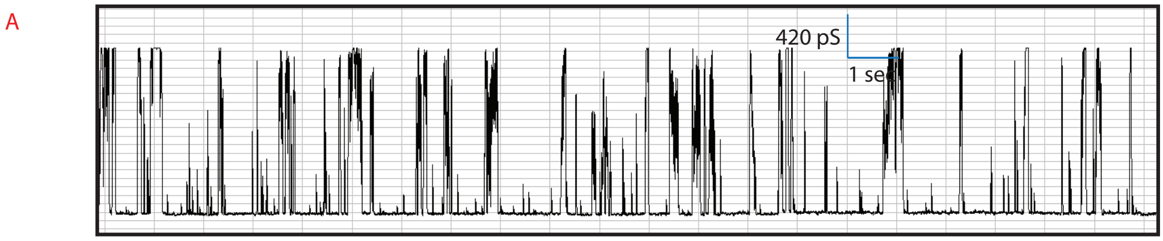

2 41-0007 1M CsCl 2uM in cmpd, +100mV

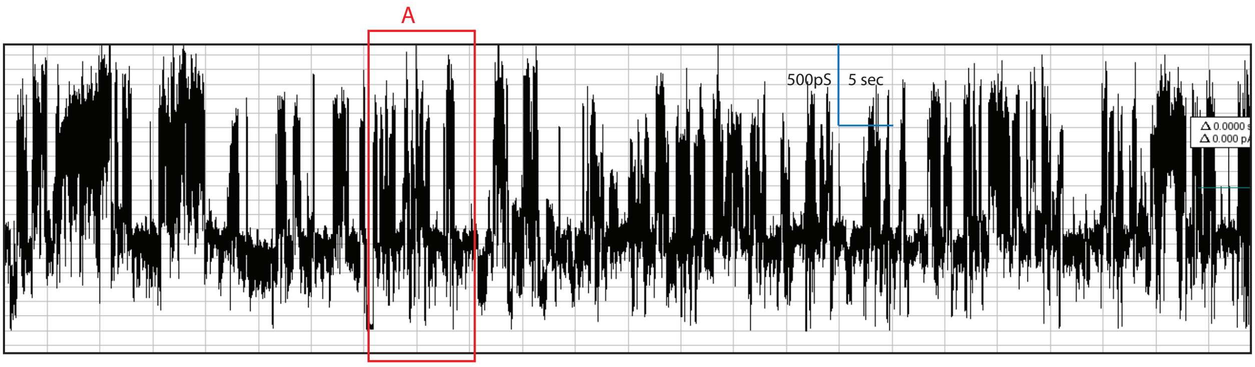

A

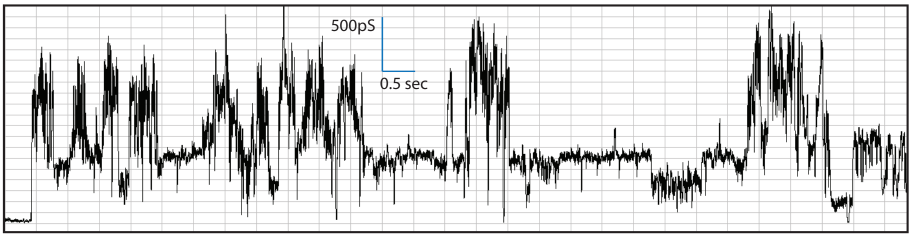

2 41-0011 1M CsCl 2uM in cmpd, -120mV

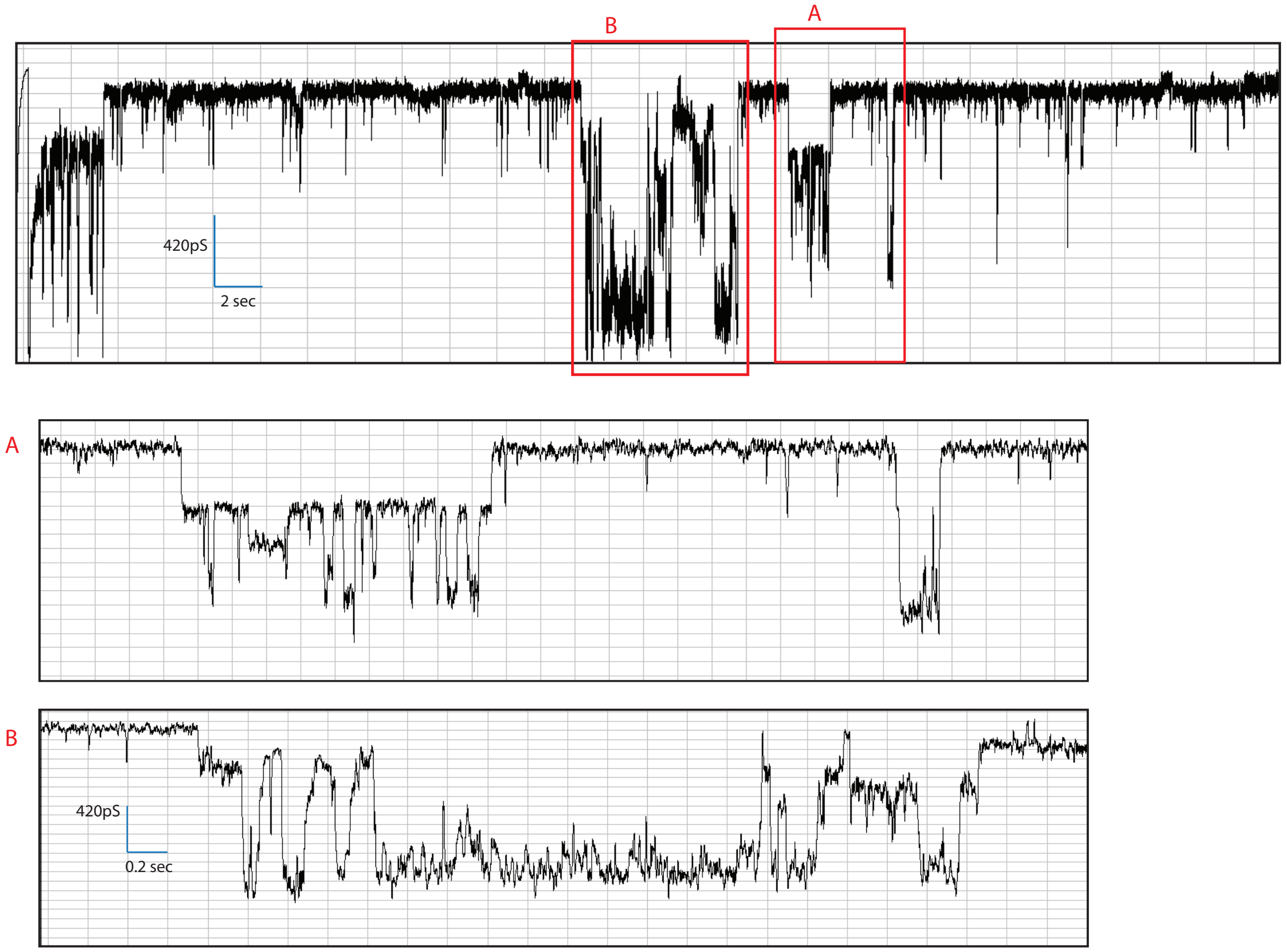

2 43-0008 1M CsCl pH7 2uM in cmpd, +120mV

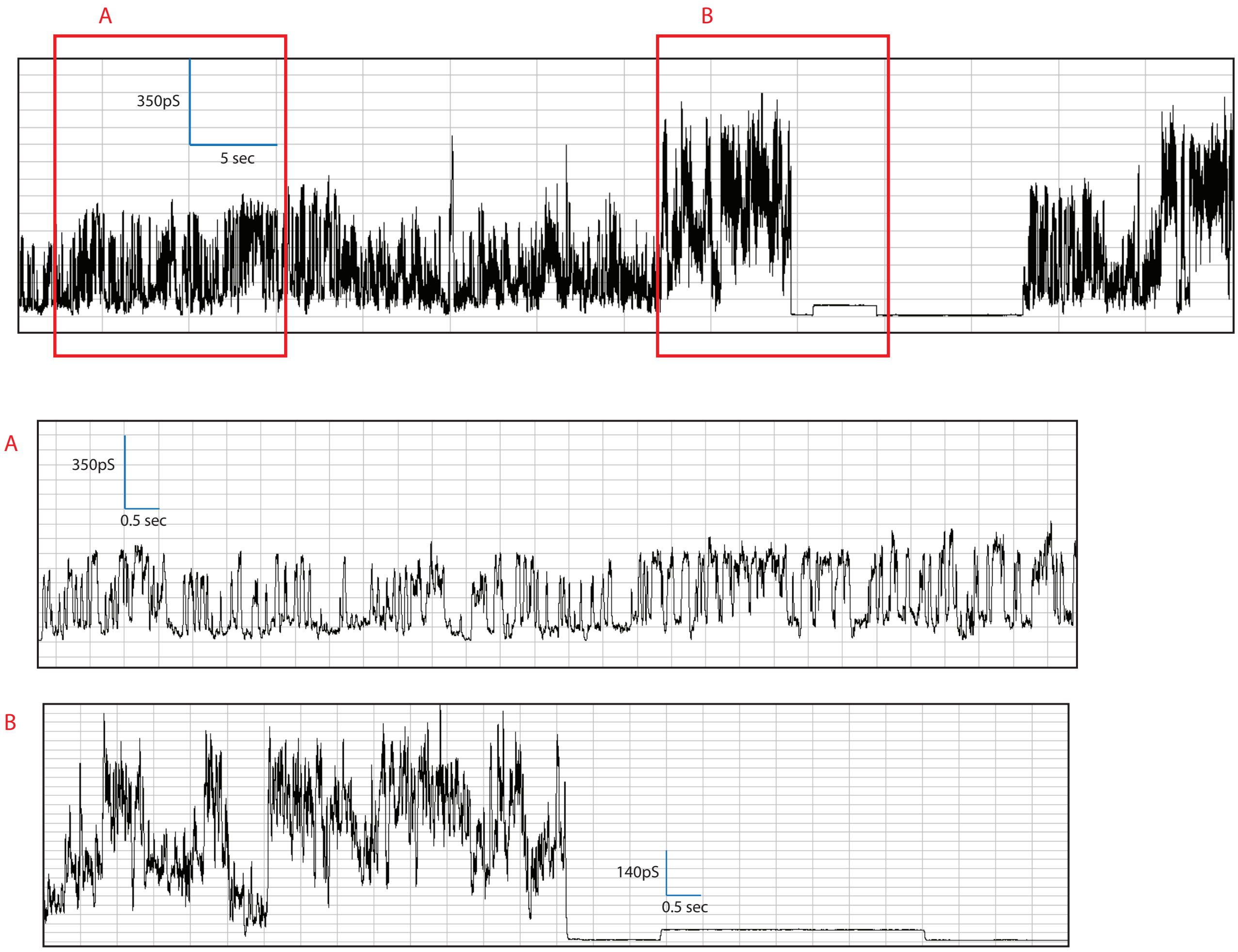

2 50-0003 1M CsCl pH 7 4uM in cmpd, +180mV

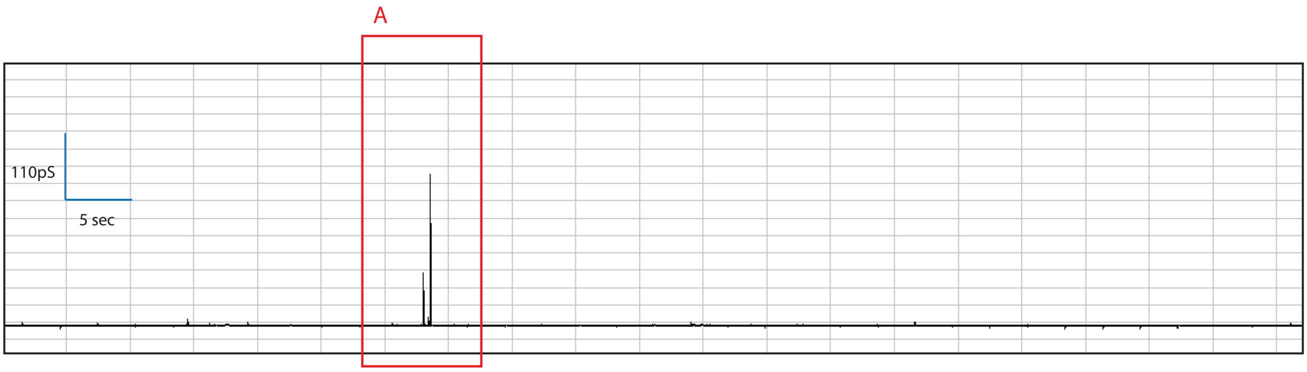

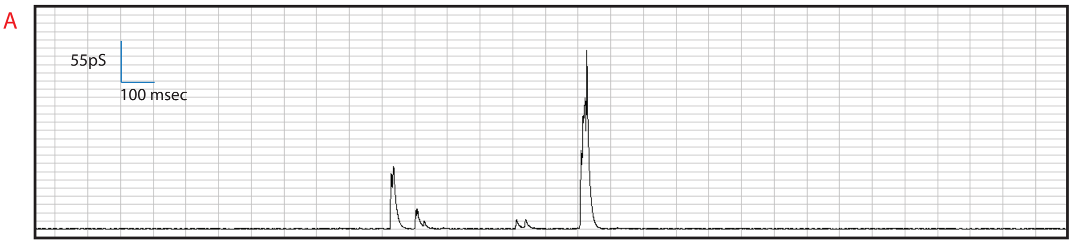

2 53-0001 1M CsCl pH 7 7.5uM in cmpd, +170mV

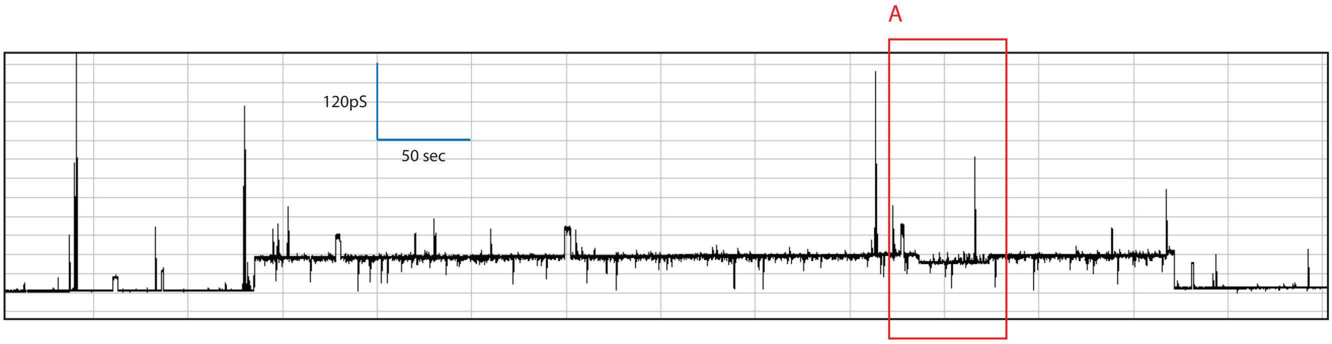

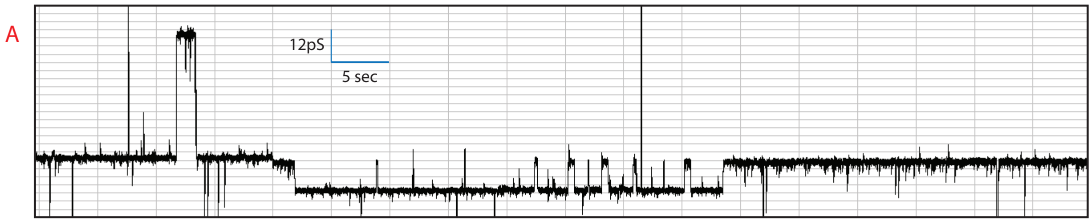

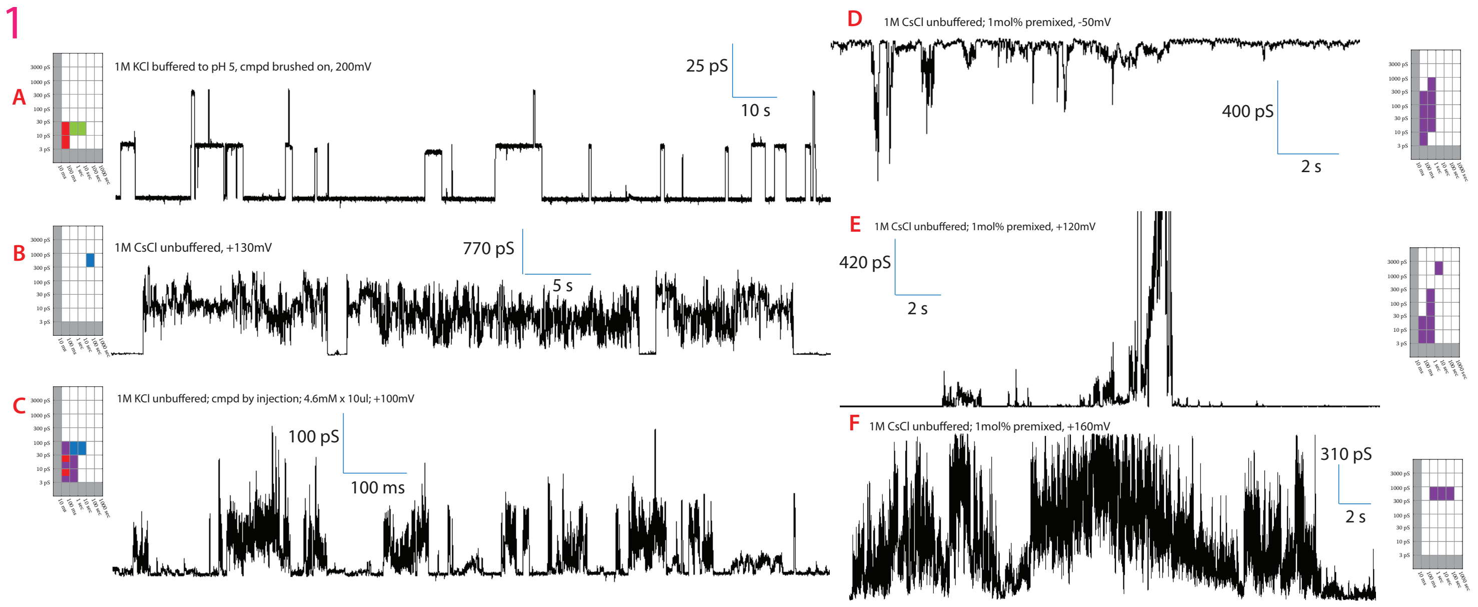
1
A
1M KCl buffered to pH 5, cmpd brushed on, 200mV
25 pS
10 s
B
1M CsCl unbuffered, +130mV
770 pS
5 s
C
1M KCl unbuffered; cmpd by injection; 4.6mM x 10ul; +100mV
100 pS
100 ms
D
1M CsCl unbuffered; 1mol% premixed, -50mV
400 pS
2 s
E
1M CsCl unbuffered; 1mol% premixed, +120mV
420 pS
2 s
F
1M CsCl unbuffered; 1mol% premixed, +160mV
310 pS
2 s

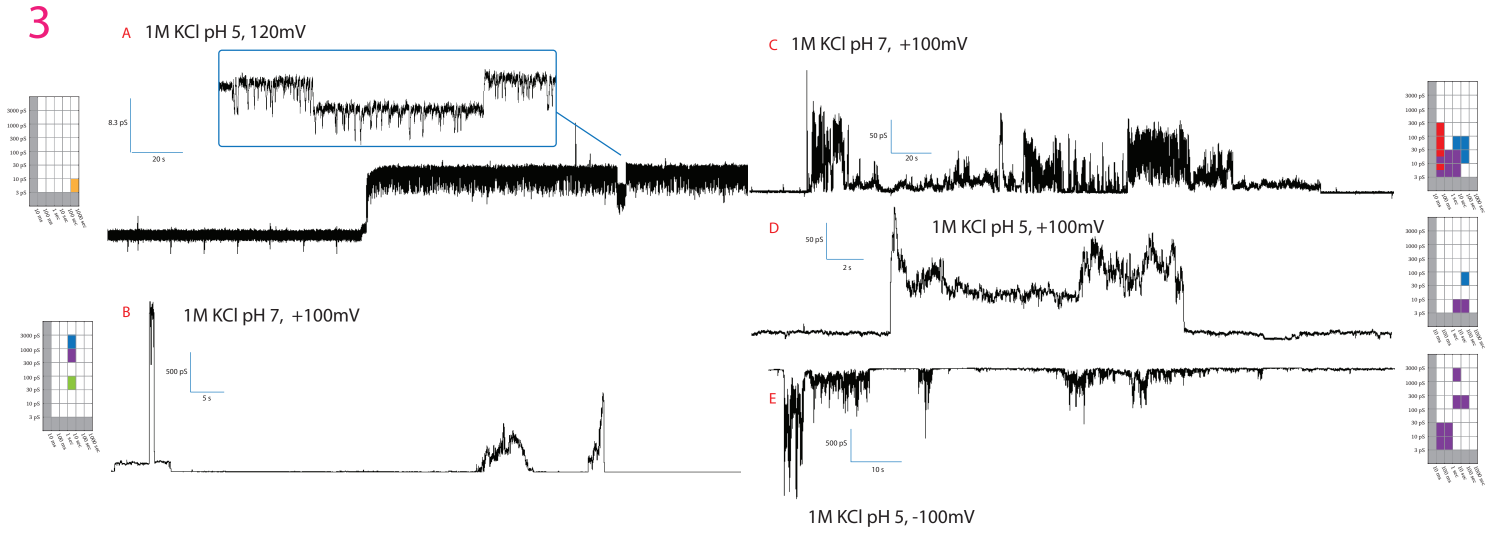
3
A
1M KCl pH 5, 120mV
8.3 pS
20 s
B
1M KCl pH 7, +100mV
500 pS
5 s
C
1M KCl pH 7, +100mV
50 pS
20 s
D
1M KCl pH 5, +100mV
50 pS
2 s
E
500 pS
10 s
1M KCl pH 5, -100mV
